# In silico characterization of microRNAs-like sequences in the genome of Paracoccidioides brasiliensis

**DOI:** 10.1590/1678-4685-GMB-2018-0014

**Published:** 2019-02-14

**Authors:** Juliana S. de Curcio, Mariana P. Batista, Juliano D. Paccez, Evandro Novaes, Célia Maria de Almeida Soares

**Affiliations:** 1 Universidade Federal de Goiás Universidade Federal de Goiás Instituto de Ciências Biológicas Laboratório de Biologia Molecular GoiâniaGO Brazil Laboratório de Biologia Molecular, Instituto de Ciências Biológicas, Universidade Federal de Goiás, Campus II Samambaia, Goiânia, GO, Brazil; 2 Universidade de Brasília Universidade de Brasília Faculdade de Medicina Programa de Pós-Graduação em Patologia Molecular BrasíliaDF Brazil Programa de Pós-Graduação em Patologia Molecular, Faculdade de Medicina, Universidade de Brasília, Brasília, DF, Brazil; 3 Universidade Federal de Lavras Universidade Federal de Lavras Departamento de Biologia Minas Gerais Brazil Departamento de Biologia, Universidade Federal de Lavras, Minas Gerais, Brazil

**Keywords:** Fungi, mycoses, Dicer, Argonaute, microRNAs

## Abstract

Eukaryotic cells have different mechanisms of post-transcriptional regulation. Among these mechanisms, microRNAs promote regulation of targets by cleavage or degradation of the mRNA. Fungi of the *Paracoccidioides* complex are the etiological agents of the main systemic mycosis of Latin America. These fungi present a plasticity to adapt and survive in different conditions, and the presence of microRNAs-like molecules could be part of the mechanisms that provide such plasticity. MicroRNAs produced by the host influence the progression of this mycosis in the lungs besides regulating targets involved in apoptosis in macrophage, activation of T and B cells and the production of cytokines. Therefore, this work analyzed the presence of regions in the genome of this fungus with a potential to encode microRNAs-like molecules. Here we show by analysis of sequence similarity the presence of 18 regions, putatively coding for microRNAs-like molecules in the *Paracoccidioides brasiliensis* genome. We also described the conservation of dicer and argonaut proteins and the cognate transcripts induced in the yeast parasitic phase. This work represents a starting point for the analysis of the presence of those molecules in the morphological stages of the fungus and their role in fungal development.

## Introduction

MicroRNAs were originally identified in *Caenorhabditis elegans* (Lee *et al.*, 1993), and since then have been described in animals, plants and algae (Zhao *et al.*, 2007; Grimson *et al.*, 2008). In fungi, the first identified miRNAs-like molecules were described in *Neurospora crassa* (Lee *et al.*, 2010). Subsequently, several studies demonstrated their presence in different species of fungi, such as *Metarhizium anisopliae*, *Sclerotinia sclerotiorum*, *Penicillium marneffei*, *Trichoderma reesei*, *Fusarium oxysporum, Aspergillus flavus* and *Penicillium chrysogenum* (Zhou *et al.*, 2012a,b Kang *et al.*, 2013; Lau *et al.*, 2013; Chen *et al.*, 2014; Bai *et al.*, 2015; Dahlmann and Kück, 2015)*.* In fungi such as *N. crassa,* different pathways are involved in miRNAs-like production. The production of milR-1 is dependent on the presence of of Dicer and the argonaut protein qde-2p, while the biogenesis of milR-2 is independent of Dicer, but catalytic activity of argonaut qde-2p is required for the production of the pre-milRNA and the mature milR-2 (Lee *et al.*, 2010).

MicroRNAs-like molecules influence the process of host-pathogen interaction (Zhou *et al.*, 2012a; Lau *et al.*, 2013). During mycosis development, several host microRNAs are regulated in response to the presence of these pathogens. Some of the regulated processes are macrophage polarization, chemokine expression, granulocyte production, inhibition of the Th1 immune response, and negative regulation of monocyte differentiation (reviewed in Croston *et al.*, 2018). Analysis of the expression pattern of microRNAs produced by dendritic cells (DCs) after contact with *A. fumigatus* and *Candida albicans* revealed a specific response of microRNAs to the infection caused by these fungi. Some of the microRNAs induced after contact with these pathogens were miR-212 and miR-132. These microRNAs are able to promote fine-tuning of mRNA expression involved in the immune response as, for example, *BTN3A2* a gene associated with the stimulation of the adaptive immune response in DCs, as well as of *FKBP1B,* which is associated with T cell proliferation in mice, and of *KLF4* that is involved in the response of DCs to fungal infections (Dix *et al.*, 2017). During the process of germination of *A. fumigatus* conidia in lungs, different levels of microRNA and mRNA expression, were observed in the host. Repressed microRNAs in lungs infected with conidia included miR-29a-3p, miR-30c-5p, and targets of these microRNAs were genes involved in the inflammatory response, such as *Clec7, SMAD2/3* and *TGF-*β*.* The gene *Clec7a* elicits an inflammatory response due to the recognition of β-glucan on the hypha cell wall. The TGF-β signaling pathway includes the transcription factors from SMAD family, and microRNAs that regulate these transcripts were seen repressed in lung tissue. This demonstrated that conidia of *A. fumigatus* repress host microRNAs, and doing so, allow the expression of host mRNAs involved in the inflammatory response (Croston *et al.*, 2016).

Paracoccidioidomycosis (PCM) is an endemic systemic mycosis caused by fungi of the genus *Paracoccidioide*s. This genus comprises five species, *P. brasiliensis, P. lutzii, P. americana, P. restrepiensis and P. venezuelensis* (Restrepo, 1985, Turissini *et al.*, 2017). It is estimated that approximately 80% of the patients who acquire this disease are Brazilians, the remainder of the cases occur mainly in Colombia, Venezuela, Argentina and Ecuador. The lethality rate of this disease is 3% to 5%, and the number of cases in Brazil per year varies from 3360 to 5600 (Martinez, 2017). In systemic infections, the presence of a pathogen often induces a significant change in the expression profile of circulating microRNAs in the host. In this context, Singulani *et al.* (2017) analyzed the expression profiles of microRNAs produced by patients with PCM and compared these to those of healthy individuals. The data indicated the induction of expression of eight microRNAs among the individuals with PCM. Apoptosis of macrophages, activation of T and B cells, and fungal adhesion to host cells are processes that are putatively regulated by differentially expressed microRNAs, thus suggesting an influence of these molecules on the pathogen-host interaction process. Also, histopathological changes observed in the lungs of mice infected with *P. brasiliensis* at 28 and 56 days post-infection, correlated with the level of expression of the microRNAs produced by this organ. Within 28 days of infection, the lungs showed the presence of granulomas, with infiltrates of giant cells bordering the fungal cells, and the presence of phagocytic cells. At 56 days post-infection, the lungs presented a low number of yeast cells, characteristic of the resolution of the infection. Additionally, microRNAs induced at 56 days post-infection may act to reduce damage to lung tissues; for example the miRNA-26b-5p, which negatively regulates the expression of IL-6, could influence the amount of T cells at 56 days post- infection (Marioto *et al.*, 2017).

Although miRNAs-like molecules have already been described in pathogenic microorganisms, as described above and microRNAs produced by the host are modulated in response to fungal infections, the presence of microRNAs-like molecules in the genus *Paracoccidioides* is not described. In this work, we sought to identify proteins in the genomes of members of the *Paracoccidioides* genus that are involved in the post-transcriptional gene silencing pathway mediated by miRNAs. We evaluated the level of expression of transcripts encoding such proteins in the parasitic phase of *P. brasiliensis*. Using miRNA-like sequences identified in the fungi *N, crassa*, *C. neoformans* and *P. marneffei*, we performed an *in silico* identification of miRNAs-like sequences in the genome of *P. brasiliensis*. We identified dicer and argonaut proteins that are conserved in species of the *Paracoccidioides* complex, as well as sequences in the genome of *P. brasiliensis* that are predicted as microRNAs-like genes. To our knowledge, this is the first in-depth in *silico analysis* complemented with experimental results reporting the presence of microRNA-like coding regions in this important human pathogen.

## Material and Methods

### In *silico analysis* of protein homologs in *Paracoccidioides* spp. involved in the post-transcriptional gene silencing pathway

The sequences of dicer and argonaute proteins present in the UNIPROT database were used for searching for homologous proteins in *Paracoccidioides* spp. We compared these sequences with those described for other fungal species: *N. crassa, C. neoformans* H99, *Histoplasma capsulatum*, *Aspergillus nidulans* and *A. fumigates* (Galagan *et al.*, 2003; Nakayashiki *et al.*, 2006; Janbon *et al.*, 2010). Prediction of similarity and identity among homologous proteins was performed using the BLASTp tool implemented in the NCBI database. Sequence alignment and protein domain prediction were performed using CLUSTALX2 and the PFAM databases, respectively (Larkin *et al.*, 2007; Finn *et al.*, 2015).

### Phylogenetic analysis of RNA-induced silencing complex proteins in fungi

The RNA-induced silencing complex proteins dicer and argonaute from *Paracoccidioides* spp., *N. crassa, C. neoformans* H99, *H. capsulatum*, *A. nidulans*, *A. fumigatus* were used for phylogenetic analysis. A phylogenetic tree was constructed by multiple sequence alignments using CLUSTALX2. The tree was generated by the neighbor-joining method and visualized by TreeView software (Saitou and Nei 1987; Thompson *et al.*, 1997). Branch robustness was estimated using 10,000 bootstrapped replicates. The accession numbers for each protein used in this comparison are described in Table S1.

### Culture and maintenance of *P. brasiliensis* and RNA extraction

*P. brasiliensis* (ATCC 32069) yeast cells were cultivated in Sabouraud medium at 36 ºC (Sharma *et al.*, 2010). After five days of growth in solid medium, yeast cells were inoculated in liquid medium and cultivated for 18 h at 36 ºC at 150 rpm. RNA from yeast cells cultured on this condition was extracted using the Trizol method (Silva-Bailão *et al.*, 2014).

### Detection of transcripts from the RNA-induced silencing complex in *P. brasiliensis*

The detection of transcripts involved in the post-transcriptional gene silencing pathway was performed by qRT- PCR. The primer sequences for the amplification of Dicer 1 (*dcr 1*), Dicer 2 (*dcr 2*), Argonaute 1 (*ago-1*)*,* Argonaute 2 (*ago-2*) and Actin (*act*) are described in Table S2. In brief, total RNA extracted from *P. brasiliensis* was treated with DNase (RQ1 RNase-free DNase, Promega) and subjected to reverse transcription (SuperScript III First-Strand Synthesis SuperMix; Invitrogen, Life Technologies) following the manufacturer’s recommendation. SYBR green PCR master mix (Applied Biosystems, Foster City, CA) was used in the qRT-PCR assays performed in a Step OnePlus system (Applied Biosystems. Normalization of the Ct values was done using the gene encoding an actin protein (GenBank XP_010761942). Standard curves were generated by 1:5 dilution of the cDNA, and the relative expression levels of the transcripts were calculated using the standard curve method for relative quantification (Bookout *et al.*, 2006).

### *In silico* prediction of miRNAs-like molecules in the *P. brasiliensis* genome

For *in silico* prediction of miRNAs-like molecules in the genome of *P. brasiliensis*, a literature search was performed to obtain sequences of mature miRNAs-like described in other fungi, including *F. oxysporum*, *P. marneffei*, *A. flavus*, *T. reesei*, *M. anisopliae*, *C. neoformans*, *N. crassa,* and in extracellular vesicles of *C. neoformans*, *C. albicans*, *Saccharomyces cerevisiae,* and *P. brasiliensis* (Lee *et al.*, 2010; Jiang *et al.*, 2012; Zhou *et al.*, 2012a and b; Lau *et al.*, 2013; Kang *et al.*, 2013; Chen *et al.*, 2014; Peres da Silva *et al.*, 2015; Bai *et al.*, 2015).

The sequences of such miRNA-like molecules were subjected to Blastn comparison against the *P. brasiliensis Pb*18 genome, using the task blastn-short option of the Blastn program (Camacho *et al.*, 2009). Using a threshold E-value < 0.10, alignments were obtained with at least 16 bp, and identities above 95%. The results were converted into GFF files by means of a custom script written in Perl. The GFF files contain information about the position of genes, or of any other element in the reference genome. The InrsectBED tool from the BEDTools package (Quinlan and Hall 2010) was employed for identifying the position of the BLAST hits in relation to the annotation of the *P. brasiliensis* genome. Only the sequences of mature miRNAs-like that were not recorded in gene regions were considered for further analysis. After these steps, a Fasta file containing the sequences of the mature miRNAs-like molecules that aligned to the genome of *Pb*18 in non-gene regions was generated. The data in Fasta file format was considered 35 bp or 50 bp above or below the alignment region.

These sequences were analyzed for secondary structure in the RNAfold database, seeking to identify those that form hairpins similar to those of known pre-miRNAs. Potential miRNA sequences were manually revised using the following parameters: (A) the minimum size of the pre-miRNA could not be less than 45 nucleotides; (B) the pre-miRNA folded into a perfect stem-loop hairpin secondary structure; (C) the values of the minimum free energy (MFE) of folding should be at least -7 kcal/mol; (D) maximum mismatch of six between the miRNA/miRNA*duplex, where (*) means the similar-sized fragment derived from the opposing arm of the pre-microRNA. These criteria were adopted to predict true microRNAs-like candidates (Dehury *et al.*, 2013). As pre-miRNA size is variable and unknown for some cases in our study, we adjusted the size of the retrieved regions upstream and downstream (35-50 bp) of the alignment to search for a typical miRNA hairpin structure with RNAfold database (Lorenz *et al.*, 2011, 2016). Manual checking of real pre-microRNAs has already been used in other bioinformatics studies (Dehury *et al.*, 2013). All MFE values were expressed as negative kcal/mol and adjusted MFE (AMFE) represented the MFE of 100 nucleotides, calculated by (MFE length of RNA sequence) x100. The minimal folding free energy index (MFEI) was calculated by the equation: MFEI = AMFE/(G+C)% (Zhang *et al.*, 2006).

### Polyadenylation, reverse transcription and poly(T) adaptor RT-PCR

After the *in silico* prediction of microRNAs-like in the genome of *P. brasiliensis,* five potential candidates were chosen for validation experiments, based on the highest MFE values: *Pb*-milR-11/Supercontig_2.3:1128222-1128358(-); *Pb*-milR-7/ Supercontig_2.6:955763_955879(+); *Pb*-milR-6/Supercontig_2.5:587199-587317(+) and *Pb*-milR-1/Supercontig_2.14:171583_171670(+). We also chose a candidate with the lowest MFE value *Pb*-milR-4/Supercontig_2.12:21200_21275(-). Validation experiments of microRNAs-like candidates employed the polyA tail method (Balcells *et al.*, 2011). Initially, yeast cells and mycelium of *Pb*18 were cultured in Sabouraud medium (Sharma *et al.*, 2010) for 18 h, and the RNA of both conditions was extracted using TRIzol reagent (Sigma). Total extracted RNA was treated with DNase (RQ1 RNase-free DNase, Promega). The treated total RNA (3 μg) was polyadenylated with ATP by poly(A) polymerase (Biolabs) using 4 μl of 10 x polyA buffer, 4 μl of 10 mM ATP, 0.4 μl of polyA polymerase corresponding to 2 units, and 3 μg of RNA. Water was added to complete the reaction to a final volume of 20 μl. The polyadenylation reaction was performed at 37 ºC for 75 min and 65 ºC for 25 min, according to the manufacturer’s instructions. After this step, the polyadenylated RNAs were submitted to reverse transcription with SuperScrip II Reverse Transcriptase (Invitrogen). cDNA synthesis was done using 0.8 μl of 20 nM poly(T) adapter, 4 μl of 10 mM dNTPS, 8 μl of 5 x buffer, 1.5 μl of 25 mM MgCl_2_, 0.5 μl of RNAse inhibitors, 1.25 μl of reverse transcriptase, and 5 μl of polyadenylated RNA. The conditions for cDNA synthesis were: 42 ºC for 135 min and 85 ºC for 10 min. Qualitative RT-PCR assays were performed at 94 °C for 2 min, followed by 40 cycles of 95°C for 15 s, 57°C or 57.6ºC for 5 s, and 72°C for 20 s. and a final elongation step at 72°C for 5 min. The amplification products were visualized on 3% (w/v) agarose gels, and product size the potential microRNAs-like molecules was calculated by linear regression. The primers used for the amplification of microRNAs-like are listed in Table S2.

## Results

### *In silico* analysis indicates the presence of proteins involved in post-transcriptional gene silencing in *Paracoccidioides* spp.

A flowchart of the steps for *in silico* prediction of proteins involved in gene silencing by RNA interference and for potential miRNAs-like is shown in Figure S1. Our analysis allowed the identification of dicer and argonaute proteins in *Paracoccidioides* spp. (Table 1). *P*. *brasiliensis* (*Pb*18), *P. americana* (*Pb*03), and *P*. *lutzii* presented two dicer (Dcr-1p and Dcr-2p) and two argonaute (Ago-1 and Ago-2) proteins. *C. neoformans* Serotype D presented two paralogous genes for argonaute (*ago1* and *ago2*) and dicer (*dcr1* and *dcr2*), in contrast to the *C. neoformans* serotype A (H99), which contains a single gene for argonaute (*ago1*) and two paralogous genes for dicers (*dcr1* and *dcr2*) (Janbon *et al.*, 2010). Studies with *N. crassa* demonstrated the presence of two genes that encode dicers (*qde-2* and *dcr*) and two that code for argonautes (*Sms-2* and *Sms-3*) (Galagan *et al.*, 2003). The *A. nidulans* genome has only one gene that codes for dicer (ANID_10380) and one that codes for argonaute (ANID_01519), while *A. fumigatus* has two homologs that encode for two dicer (AFUA_5G11790/AFUA_4G02930) and two argonaute proteins (AFUA_3G11010/AFUA_8G05280) (Nakayashiki *et al.*, 2006; Janbon *et al.*, 2010). Hence, the number of proteins involved in post transcriptional gene silencing in fungi of the *Paracoccidioides* complex is similar in number to other fungi. Furthermore, the analysis of identity indicates major similarity among proteins from *Paracoccidioides* spp., *H. capsulatum,* and *A. fumigatus* (Table 1).

**Table 1:** *In silico* prediction of proteins potentially involved in the post-transcriptional gene silencing machinery in *Paracoccidioides* spp.

Proteins ^a^	Identity/e-value ^b^	Identity/e-value ^b^	Identity/e-value ^b^	Identity/e-value ^b^	Identity/e-value ^b^	Identity/e-value ^b^
Argonaut 1 ^c^	*C. neoformans* (H99) CNAG_04609	*N. crassa* NCU09434	*A. fumigatus* Af230 Afu3g11010	*A. nidulans*	*H. capsulatum* H143 HCDG_08528	*H. capsulatum* G186AR HCBG_06692
*P*. *brasiliensis* (*Pb*18) PADG_00716	28%/2 e-81	40%/0.0	54%/0.0	ND	76%/0.0	76%/0.0
*P. americana* (*Pb*03) PABG_02302	28%/2 e-82	40%/0.0	54%/0.0	ND	74%/0.0	74%/0.0
*P. lutzii* PAAG_11422	28%/ 2e-80	40%/9e-179	54%/0.0	ND	74%/0.0	74%/0.0
Argonaut 2 ^c^	*C. neoformans*	*N.crassa* NCU04730	*A. fumigatus* Af230 Afu8g05280	*A. nidulans* ANID_01519	*H. capsulatum* H143 HCDG_00823	*H. capsulatum* G186AR HCBG_03944
*P*. *brasiliensis* (*Pb*18) PADG_03108	ND	37% 2 e-177	50%/0.0	49%/0.0	78%/0.0	76%/0.0
*P. americana* (*Pb*03) PABG_00673	ND	37%/7e-175	50%/0.0	49%/0.0	78/00	76%/0.0
*P. lutzii* PAAG_03231	ND	37%/7e-175	50%/0.0	49%/0.0	78%/0.0	77%/0.0
Dicer 1 ^c^	*C.neoformans* CNAG_02745	*N.crassa* NCU08270	*A. fumigatus* Afu5g11790	*A. nidulans*	*H. capsulatum* H143 HCDG_06891	*H. capsulatum* G186AR HCBG_01751
*P*. *brasiliensis* (*Pb*18) PADG_11946	29%/2 e-38	39%/0.0	53%/0.0	ND	62%/0.0	72%/0.0
*P. americana* (*Pb*03) PABG_04917	24%/ 4e-37	40%/0.0	54%/0.0	ND	67%/0.0	70%/0.0
*P. lutzii* PAAG_11489	24%/3e-33	38%/0.0	53%/0.0	ND	65%/0.0	71%/0.0
Dicer 2 ^c^	*C. neoformans*	*N.crassa* NCU06766	*A. fumigatus* Afu4g02930	*A. nidulans* ANID_10380	*H. capsulatum* H143 HCDG_06620	*H. capsulatum* G186AR HCBG_01136
*P*. *brasiliensis (Pb*18) PADG_07189	ND	32%/0.0	38%/0.0	37%/0.0	70%/0.0	68%/0.0
*P. americana* (*Pb*03) PABG_05105	ND	32%/0.0	38%/0.0	37%/0.0	70/0.0	68%0.0
*P. lutzii* PAAG_00072	ND	34%/0.0	39%/0.0	37%/0.0	70%/0.0	69%/0.0

a Predicted name of the protein involved in the post-transcriptional gene silencing mediated by miRNAs;

b The sequence identity values and e-value were obtained through the BLASTp tools of the NCBI database; (http://blast.ncbi.nlm.nih.gov/Blast.cgi?PROGRAM=blastp&PAGE_TYPE=BlastSearch&LINK_LOC=blasthome)

c Proteins of *Paracoccidioides* spp. homologous to *C. neoformans* (H99), *N.crassa, A. fumigatus*, *A. nidulans*, *H capsulatum* G186AR and H143 strains. N.D. (not described).

The phylogenetic analysis of Ago-1p and Ago-2p, Dcl-1p and Dcl-2p showed that the sequences present in *Paracoccidioides* spp. are closely related to *Blastomyces dermatittidis, H. capsulatum, P. chrysogenum,* and *Aspergillus* spp. (Figure S2). With phylogenetic distances from 0.98 to 1 it is possible to infer that the proteins are orthologous, possibly having a similar function among different fungi. Correspondingly, a study by Lau *et al.* (2013) showed that Dcl-2p of *P. marneffei*is is more closely related to those of the thermal dimorphic pathogenic fungi *H. capsulatum, B. dermatitidis,* and *C. immitis,* than to *P. chrysogenum* and *Aspergillus* spp.

### The post-transcriptional gene silencing machinery is highly conserved in *Paracoccidioides* spp. and other fungi

**Figure 1:**
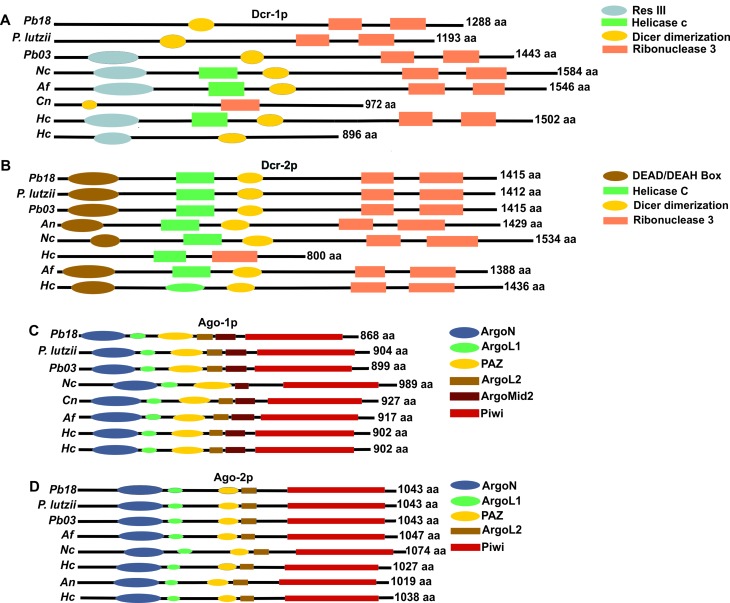
Domains identified in proteins involved in the post-transcriptional gene silencing pathway. (AD) Domains present in dicers and argonauts proteins from *P. brasiliensis* (Pb18), *P. lutzii* (Pb01) and *P. americana* (Pb03). The accession numbers of the proteins are as follows. Dicer 1: (PADG_11946; PABG_04917; PAAG_11489; NCU08270; Afu5g11790; CNAG_02745; HCBG_01751; HCDG_06891), Dicer 2: (PADG_07189; PAAG_00072; PABG_05105; ANID_10380; NCU06766; HCDG_06620; Afu4g02930; HCBG_01136) Ago1: (PADG_00716; PAAG_11422; PABG_02302; NCU09434; CNAG_04609; Afu3g11010; HCBG_06692; HCDG_08528) Ago2: (PADG_03108; PAAG_03231; PABG_00673; Afu8g05280; NCU04730; HCBG_03944; ANID_01519; HCDG_00823).

The prediction of protein domains performed using the PFAM database allowed the identification of conserved domains in dicers and argonauts (Figure 1). It is important to highlight that the main components involved in miRNA processing and gene silencing include RNA polymerase (Pol II), dicer, and the silencing complex induced by RNA (Bartel *et al.*, 2004). Studies analyzing domains of the canonical dicer of *N. crassa* Dcr-2p demonstrated the presence of DEAD box, Helicase C, ribonuclease 3 and dicer dimerization domains. Dicer 1 from *N. crassa* contains at its N-terminal region a RESIII domain instead of a DEAD box. The homologue from *C. neoformans* has only the ribonuclease 3 and dicer dimerization domains (Janbon *et al.*, 2010), and the analysis of the Dcr-1p proteins from *Pb*18 and *P. lutzii* (*Pb*01) indicates similarity to the homologous protein found in *C. neoformans*, since it possesses only the dicer dimerization and the ribonuclease 3 domains (Figure1A). On the other hand, the analysis of the same homologue in *Pb*03 demonstrated higher similarity to the dicer dimerization, ribonuclease 3 and RESIII domains found in the *N. crassa* homolog (Figure 1A). Therefore, we can conclude that the general domain arrangement of this protein is not fully conserved among members of the complex *Paracoccidioides.*

Furthermore, the analysis of protein domains from *Paracoccidioides* spp. demonstrated that Dcr-2p contains the Dicer dimerization, DEAD/DEAH box, Helicase C, and ribonuclease 3 domains similar to those present in other dimorphic and filamentous fungi, except for *H. capsulatum* (H143) (Figure 1B). Comparing the members of the *Paracoccidioides* complex, depicted in (Figure 1), it is apparent that Dcr-2p is conserved among species of *Paracoccidioides.*

Previous studies described the dicer dimerization domain as being similar to the binding domain of dsRNA (Dlakic, 2006), while the DEAD/DEAH-box helicase domain interacts with the pre-miRNA loop, facilitating the alignment of this molecule to the RNase III domain for precise pre-miRNA cleavage (Tsutsumi *et al.*, 2011). Additionally, the Helicase C domain binds to the dsRNA loop region and checks the size. Furthermore, this domain also measures the distance between two adjacent nucleotides in the 3`region of the mRNA molecule, with the loop region verifying the pre-miRNA sequence before cutting (Tsutsumi *et al.*, 2011). Dicers proteins have a ribonuclease 3 domain responsible for the cleavage of pre-miRNA, generating a duplex of small RNAs with two adjacent nucleotides in the 3’end (Zhang *et al.*, 2004). In this regard, our data suggest that these proteins are able to process double-stranded RNA, since we identified conserved domains among dicers from *Paracoccidioides* spp.

Our data also indicate that Ago-1p from *Paracoccidioides* spp. contains PIWI, PAZ, ArgoL1, ArgoL2, ArgoMid2 and ArgoN domains (Figure1C), while Ago-2p contains PIWI, PAZ, ArgoL1, ArgoL2 and ArgoN domains (Figure1D). The analysis of argonaute proteins from *N. crassa, P. chrysogenum*, *P. marneffei* and *C.neoformans* indicated the presence of PAZ and PIWI domains that are conserved among different species of fungi and that play a role in RNA interference processes (Nakayashiki, 2005; Joshua-Tor, 2006; Janbon *et al.*, 2010; Lau *et al.*, 2013; Dahlmann and Kück, 2015). Interestingly, the PIWI domain present in argonaute shows a tertiary structure similar to the RNase H family and has been described as being involved in targeting mRNA cleavage, while the PAZ domain is responsible for the binding and transfer of small RNAs to the RISC complex (Lingel *et al.*, 2003; Yan *et al.*, 2003; Song *et al.*, 2004).

**Figure 2:**
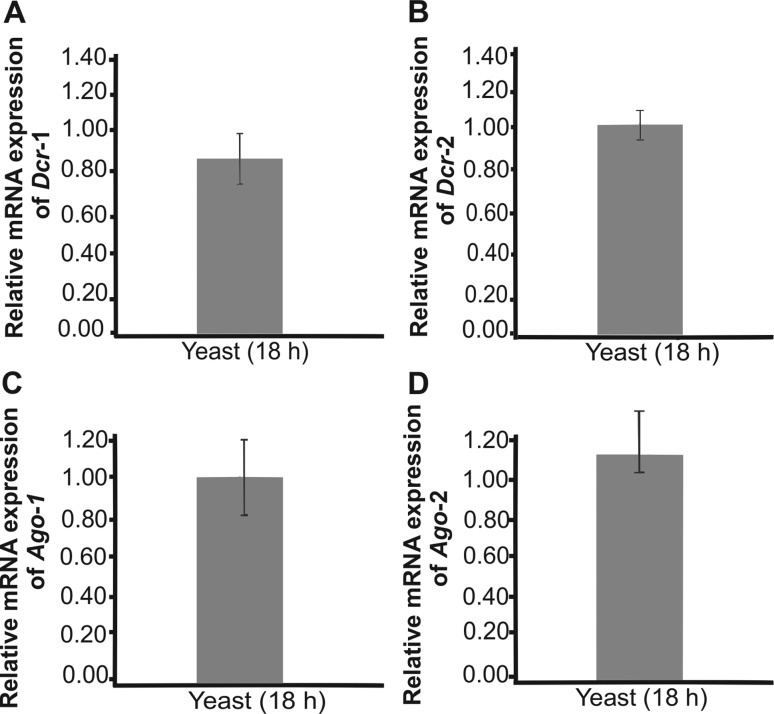
Genes involved in the processing of microRNAs-like are induced in the parasitic phase of *P. brasiliensis*. Quantitative RT-PCR was performed with transcripts of Pb18 yeast cells cultivated in liquid Sabouraud medium for 18 hours. Expression values of genes involved in the processing of microRNAs (*dcr-1, dcr-2, ago-1, ago-2*) were calculated using actin as the endogenous control. Data are expressed as mean ? standard deviation from triplicates.

After *in silico* prediction of proteins involved in gene silencing mediated by miRNAs-like molecules, the respective transcripts levels were quantified by qRT-PCR. We performed this study in *Pb*18, a highly investigated species in the *Paracoccidioides* complex. The transcripts were assessed in yeast cells cultivated at 18 h of growth. As shown in Figure 2, transcripts for dicers and argonauts are clearly detected after 18 h of growth. Detection of the transcripts encoding argonaute and dicer proteins reinforce our *in silico* data, depicting the active pattern of transcription of the cognate genes.

### Identification of miRNAs-like sequences in the *P. brasiliensis Pb*18 genome

**Figure 3:**
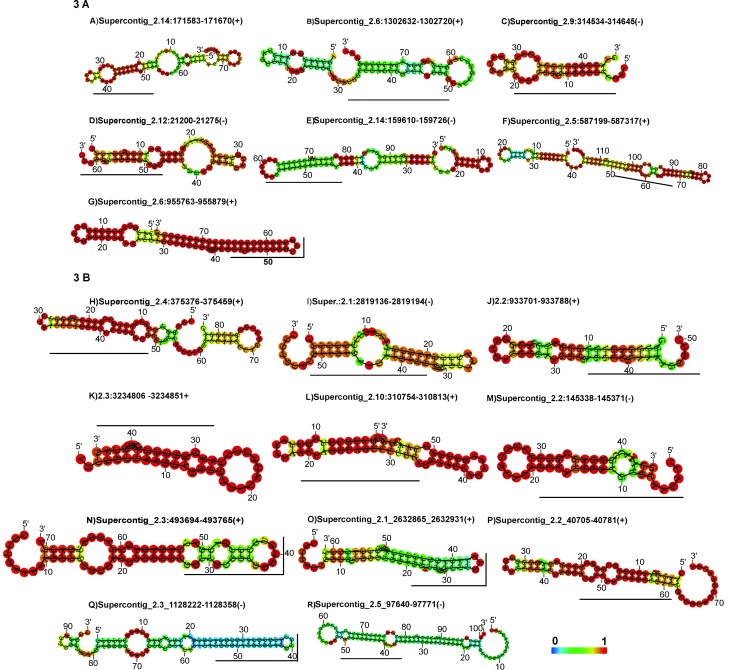
Representation of the newly-identified potential pre-miRNAs in *P. brasiliensis.* The matured miRNA portion is highlighted in black bar. The structures were generated using the Rfold program. The actual size of the precursors may be slightly shorter or longer than shown in the figures. The structures are colored according to the base pairing probabilities. Red color denotes high probability, as represented in the color bar.

In order to identify putative miRNAs-like in the genome of *P*. *brasiliensis* (*Pb*18) we analyzed the similarity between microRNA-like sequences characterized in fungi or present in vesicles secreted by these microorganism, with regions of the genome of *P. brasiliensis Pb*18. The miRNAs-like sequences were obtained from the literature. In the first step we analyzed the microRNAs-like characterized in other fungi, and from this analysis we identified 134 potential miRNAs-like sequences (Table S3). Remarkably, amongst these miRNA-like sequences, 20 sequences aligned in the genome of *P.brasiliensis* with identities higher than 95% (Table S3). Moreover, the analyzed sequences demonstrated a high percentage of alignment with *A. flavus* (7.5%) and *N. crassa* (5%) genomes (Figure S3). Furthermore, we observed that seven sequences matched all the criteria (Dehury *et al.*, 2013) to be considered mature miRNA-like molecules in the *P. brasiliensis* (*Pb*18) genome (Table 2). The analysis of the predicted secondary structure of these sequences corroborated the features of a pre-miRNA hairpin and free energy folding (Table 2 and Figure 3A). In fact, all the Fasta sequences were analyzed in relation to these two characteristics by the RNAfold program. In an analysis for miRNAs-like sequences described to be present in secreted vesicles (Peres da Silva *et al.*, 2015) a total number of 1477 miRNA sequences was described in vesicles of other fungi species, but only 100 sequences were aligned in the genome of *P. brasiliensis* with identity higher than 95% (Table S4). It is important to note that in many cases, the same miRNAs-like sequence aligned in more than one region in the *P. brasiliensis* genome. From the total of 100 sequences that aligned in the genome of *P. brasiliensis*, only 11 presented all the criteria for potential miRNAs-like molecules (Dehury *et al.*, 2013) (Table 2 and Figure 3B). The microRNAs-like predicted *in silico* for *P. brasiliensis Pb*18 that are already described in other fungi are listed in Figure 3A, whereas the microRNAs present in vesicles with similarity are shown in Figure 3B.

**Table 2:** Potential miRNAs-like identified in the genome of *Paracoccidioides brasiliensis Pb*18.

Mature microRNA sequence ^(a)^	Supercontig ^(b)^	Pre-microRNA sequence ^(c)^	Microorganism of origin/MFE ^(d)^
GGGAGAGGGGGCCGUUG	Super.:2.14:171583-171670(+)	GUCUAGUAGCACCAGCUAAGGGCCCUAGAACCACUGGGAGAGGGGGCCGUUGCACACUGGCGGGACAGGGCAAGGGAAGCCGAAGGU	*Aspergillus flavus*- milR-23/(-26.50)
UGGCCGAGUGGUUAAGGC	Super.:2.6:1302632-1302720(+)	AGUUAUGAAACUAGUGUUAAAAAUGGCAAGAUGGCCGAGUGGUUAAGGCGUACCGUUCAAGUCACAUGAACACUUGGAAA	*Fusarium oxysporum*-milRNA_2a/Fox_milRNA_2b/(-14.10)
GAGAUGGCCGAGCGGUCC	Super.:2.9:314534-314645(-)	GAACCGUUUCCAACAAGCAAUGGGUGAGAUGGCCGAGCGGUCCAA	*Aspergillus flavus*-milR-9/(-10.10)
UAGGAUUAGGAUUAGG	Super.:2.12:21200-21275(-)	UUAGGAUUAGGAUUAGGGUCAGUUAAGGAUUAGGGUCUGAGAAAUAUGAUAAAAUCGCUACCG	*Penicillium marneffei*-milR-MC7/(-7.60)
GCGGAGAGGGGUGGAA	Super.:2.14:159610-159726(-)	UAAAUGAGUGAGUGACCUUGCUAUCACAUACCUUUUUAUAUAGCGGAGAGGGGUGGAAGUAGUUAAUUUAUCAUUUUGUAGUUAAUUUAUUUAGCCAACG	*Aspergillus flavus*-milR-6/(-18.90)
AAAUCACCUUCACCUUCA	Super.:2.5:587199-587317(+)	UUUUGCCUCGCUUUAUUGUGUGCGAGUGUUGGGGCAGAUUUCGAGUAUUAAAAUCACCUUCACCUUCAAGGGGCUUAAUAGCACUUCUUUGAUGUAUGGUGUUUUACCAUAUUUAUUA	*Neurospora crassa-*milR-4/(-26.70)
GCGGACGCGAUGGUGG	Super.:2.6:955763-955879(+)	CAUGGCCUGCAUGUGGGAUGCAGAAAUGAGGAGGUGAUUUGCGGACGCGAUGGUGGCCGUUGUUCUGUUCACUUUUUG	*Penicillium marneffei*-milR-MC17/(-28.56)
AUCCAGUUCUCUGAGGG	Super.:2.4:375376-375459(+)	CUCGGUGCAUAUCAGUCGAACUACGGGUCAAAUCCAGUUCUCUGAGGGCUAUCCAUUUCUUUUCAACCCAGAUCGCGUUGGAG	*Saccharomyces Cerevisiae+/*(-16.90)
UGAAGAGAAGAAGAUU	Super.:2.1:2819136-2819194(-)	CAUGAAGAGAAGAAGAUUAUUUCAGCACAGCUUAAUCUUAUCAGUCCUCUUCU	*Candida albicans+*/(-10.50)
AGGCUGCAAAAGGGUU	Super.:2.2:933701-933788(+)	CUUUGAGUUUGCCAUCCACGGUUGAUGUGAAGGUGAGGCUGCAAAAGGGUU	*Candida albicans+*/(-10.10)
AAGUGCUUAUAGUGCAG	Super.:2.3:3234806 -3234851(+)	AUCUGCUAAGCAGUUGUCAUAGUUCUCAAGUGCUUAUAGUGCAG	*Candida albicans+*/(-14.30)
ACUAGGUAGUCCUUGA	Super.:2.10:310754-310813(+)	CAGCUGCUUUGUUAAAUAACUAGGUAGUCCUUGAGUAACCAGAAAGGUUAGCAAUGGUG	*Paracoccidioides brasiliensis+*/(-15.19)
GCUAUUCAUGUGCAAGA	Super.:2.2:145338-145371(-)	GCAAACGGUGAGCGGCUUUUGAUAUAGCUAUUCAUGUGCAAGA	*C*, *albicans*/*C.neoforman+s*(-9.60)
UUUGGCUGGGGCGGGU	Super.:2.3:493694-493765(+)	GGUUGAAAAAGUUGGGCGAUGUUUUUUGGCUGGGGCGGGUUUGGCCGAGGAUGAGAGAGGUAGGAUGACUA	*S.cerevisiae*/*C.albicans/C. neoformans+/*(-15.20)
GGACUGGAUUCUUGAA	Super.:2.1:2632865-2632931(+)	GGGGAUUGGCGGUGGGUGGAGGACUGGAUUCUUGAAAAGAGGCUUCCAAGGCUCCCAUGCU	*Paracoccidioides brasiliensis+*/(-25.80)
AUGACAAACUGUUGAU	Super.:2.2:40705-40781(+)	GAAUCUGAACAGUGCGUGCUUCUAGAAGGGGGGAAUUCCUCAAGGAUGACAAACUGUUGAUUGCCCCACCCAAUGA	*Cryptococcus neoformans+s*/(-26.30)
UGUGCAUGUGCAUGUG	Super.:2.3:1128222-1128358(-)	CCGCACUGGCUAUAAGCAGGGGACAUACUCCGUACAUAUAUGUGCAUGUGCAUGUGCAUGUGCAUGUGCAUGUGCAUGCAGAGAUAAACCUCUG	*Saccharomyces cerevisiae+e*/(-33.30)
AUGGUGGAAGAACAAGU	Super.:2.5:97640-97771(-)	AAAUCGGUCUCUACUUCGUAACAUGAUCUUUUAUCUUCUCGUCUUUCUUGAUGGUGGAAGAACAAGUGUUUGGGGGGGGGCAGGGUGUUGGGAGUGAGGUU	*Saccharomyces cerevisiae+*/(-27.80)

a) miRNA sequence predicted by *in silico* analysis;

b) Alignment region;

c) Predicted sequence of pre-miRNAs from *in silico* analysis;

d) Name of miRNAs-like described in other fungi in the genome or in vesicles/ Minimum Free Energy predicted by RNAfold.

* a small variation in the position of the supercontigs occurred in some cases due to the removal of some bases after alignment for better prediction of the secondary structure.

+ The microRNAs with similarity to *P. brasiliensis*, *C. albicans*, *C. neoformans* and *S. cerevisiae* were described in secreted vesicles and classified by the authors Silva *et al.*

Our *in silico* prediction approach based on the comparison with miRNAs-like data described in other fungi or present in secreted vesicles allowed the characterization of 18 potential miRNAs-like sequences in the genome of *P. brasiliensis* (*Pb*18), with identity values above 95%, values of negative folding free energies between -7.60 kcal/mol and -33.30 kcal/mol (Tables 2 and 3), as well as evidence for hairpin formation. Previous reports have evaluated the MFEI and calculated the AMFE values (Zhang *et al.*, 2006). In plants, MFEI values were found to vary between 0.81 and 0.96, and their AMFE values between 26 and 44 kcal/mol (Mishra *et al.*, 2015). We observed lower MFEI but similar AMFE values those described in plants (Table 3). We also evaluated other features of miRNA precursors, such as the amount of uracil and adenine in their sequences. In fact, previous reports demonstrated that miRNA precursors have a higher percentage of these bases in their sequences (Zhang *et al.*, 2006). As demonstrated in (Table 3), the majority of the miRNA precursors described in *P. brasiliensis* have a high proportion of uracil and adenine in their sequence.

**Table 3:** Characteristics of miRNAs-like molecules predicted in this study.

Number of microRNA^a^	Supercontig^b^	Length^c^	MFE^d^	AMFE^e^	MFEI^f^	(G+C)%^g^	(A+U)%^h^
*Pb*-milR-1*	Super.:2.14:171583-171670(+)	17	-26.50	30.45	0.48	63.2%	36.80%
*Pb*-milR-2	Super.:2.6:1302632-1302720(+)	18	-14.10	17.62	0.42	41.25%	58.75%
*Pb*-milR-3	Super.:2.9:314534-314645(-)	18	-10.10	22.44	0.40	55.6%	44.44%
*Pb*-milR-4*	Super.:2.12:21200-21275(-)	16	-7.60	12.06	0.31	38.1%	61.9%
*Pb*-milR-5	Super.:2.14:159610-159726(-)	16	-18.90	18.9	0.55	34%	66%
*Pb*-milR-6*	Super.:2.5:587199-587317(+)	18	-26.70	22.62	0.58	39%	61%
*Pb*-milR-7*	Super.:2.6:955763-955879(+)	16	-28.56	36.61	0.69	52.56%	47.43%
*Pb*-milR-8	Super.:2.4:375376-375459(+)	17	-16.90	20.36	0.41	49.50%	50.60%
*Pb*-milR-9	Super.:2.1:2819136-2819194(-)	16	-10.50	20.19	0.56	35.8%	64.2%
*Pb*-milR-10	Super.:2.2:933701-933788(+)	16	-10.10	19.80	0.4	49.00%	51.00%
*Pb*-milR-11*	Super.:2.3:1128222-1128358(-)	16	-33.30	35.42	0.73	47.87%	51.06%
*Pb*-milR-12	Super.:2.10:310754-310813(+)	16	-15.19	25.74	0.63	40.67%	59.32%
*Pb*-milR-13	Super.:2.2:145338-145371(-)	17	-9.60	22.32	0.50	44.2%	55.8%
*Pb*-milR-14	Super.:2.3:493694-493765(+)	16	-15.20	21.4	0.41	52.11%	47.88%
*Pb*-milR-15	Super.:2.1:2632865-2632931(+)	16	-25.80	42.29	0.73	57.40%	42.60%
*Pb*-milR-16	Super.:2.2:40705-40781(+)	16	-26.30	34.60	0.71	48.60%	51.40%
*Pb*-milR-17	Super.:2.3:3234806-3234851(+)	17	-14.30	32.5	0.79	40.90%	59.1%
*Pb*-milR-18	Super.:2.5:97640-97771(-)	17	-27.80	27.52	0.58	47%	54%

Name of predicted microRNA-like in the genome of *P. brasiliensis* (*Pb*18)*;*

Region of alignment;

Size of mature microRNA-like;

Minimum free energy predicted by RNA fold;

AMFE: adjusted minimal free folding energy (kcal/mol);

MFEI: minimal free folding energy index;

Percentage of nucleotides (G/C) present in the pre-microRNA sequence;

Percentage of nucleotides (A/U) present in the pre-microRNA sequence;

* microRNAs-like used for the validation experiments.

In general, the *in silico* analyses based on similarity of sequences allowed the identification of 18 potential microRNA-like coding regions. These exhibited microRNAs-like characteristics, such as hairpin structure, MFE values similar to those already described for microRNAs-like from other microorganisms, a large amount of uracil, demonstrating that these coding regions of microRNAs-like are conserved in the genome of *P. brasiliensis.*

### Experimental validation of predictions obtained by bioinformatics

**Figure 4:**
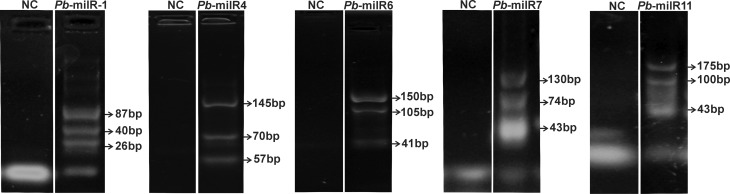
Qualitative RT-PCR of microRNAs-like predicted by bioinformatics tools. Five potential microRNAs-like were selected on the basis of the highest and lowest MFE values and were used for the validation experiments. Each reaction sample was individually fractionated on a 3% agarose gel and the fragments sizes were predicted by linear regression. RNA was obtained from *P. brasiliensis* Pb18.

The validation of our data predicted by bioinformatics was performed by qualitative RT-PCR, for five potential microRNAs-like sequences (Figure 4). The selection of these microRNAs-like was based on the highest and lowest MFE values. The presence of three amplification products was detected representing, putatively, the pri-microRNA, pre-microRNA and mature microRNA-like molecules. This method has previously been used to detect microRNAs in different cell types (He *et al.*, 2005; Chiba *et al.*, 2012). Therefore, the presence of these amplification products confirms the data predicted by bioinformatics and suggests these potential microRNAs-like candidates as being conserved in the genome of this human pathogen.

## Discussion

Proteins involved in the machinery of gene silencing by RNA interference (dicers and argonauts) are conserved in fungi of different phyla, including Ascomycota, Basidiomycota, and Zygomycota (Drinnenberg *et al.*, 2009). Our analysis demonstrated that the number of proteins involved in the post-transcriptional gene silencing pathway of *Paracoccidioides* spp. are in line with data described in the literature for other fungi, such as *C. neoformans*, *A. fumigatus,* and *N. crassa* that also present two paralogous genes for dicer and argonaute in their genomes (Galagan *et al.*, 2003; Nakayashiki *et al.*, 2006; Janbon *et al.*, 2010). In this regard, the conservation of domains in proteins involved in RNA interference in *Paracoccidioides* spp. demonstrates that these organisms retain components involved in the processing of miRNAs-like, as seen in other fungi (Lee *et al.*, 2010; Jiang *et al.*, 2012). In addition, proteins involved in the processing of microRNAs-like in *Paracoccidioides* spp. were seen to be phylogenetically related to dimorphic fungi, and, thus, are likely to perform similar functions (Lau *et al.*, 2013). Furthermore, our gene expression data reinforce the suggested conservation of the post-transcriptional gene silencing machinery mediated by microRNAs and support the concept that these genes are active in the parasitic form of this pathogenic fungus.

Different dicer and argonaut proteins are required to produce microRNAs-like molecules in fungi such as *N. crassa* (Lee *et al.*, 2010)*.* These proteins are specifically involved in the production of microRNAs-like molecules in mycelium and yeast cells. For example, the dicer 2 protein is specifically involved in the production of two microRNAs-like molecules exclusive of the mycelium phase of *P. marneffei* (Lau *et al.*, 2013). In addition, microRNAs-like molecules can also be processed via the canonical pathway, involving both both dicer and argonaut proteins (Lee *et al.*, 2010). Our data demonstrate the expression of dicers and argonauts in the yeast phase of *P. brasiliensis,* and in this same condition, the expression of five microRNAs-like was identified. Therefore, the expression of the transcripts encoding the proteins involved in microRNAs processing, possibly correlates with the identified small RNAs, since the transcripts encoding these proteins were also induced in the yeast phase. However, which proteins are specifically involved in the production of these microRNAs-like molecules still needs to be investigated.

The *in silico* prediction of microRNAs-like molecules in *P*. *brasiliensis* demonstrated that this fungus retains regions in the genome that are responsible for the genesis of mature miRNAs. Mature sequences of miRNAs conserved in different kingdoms are already described in the literature, demonstrating the importance of the conservation of these post-transcriptional gene regulation mechanisms (Lee *et al.*, 1993; Chen *et al.*, 2014). In fact, possible miRNAs-like molecules present in vesicles secreted by *P. brasiliensis* (*Pb*18) were already predicted using a strategy based on sequence similarity (Peres da Silva *et al.*, 2015). Similarly, 22 miRNA-like sequences were identified in the genome of *Humulus lupulus* employing *in silico* prediction of miRNAs based on similar sequences present in the miRBase database (Mishra *et al.*, 2015), demonstrating the efficiency of this strategy to characterize microRNAs.

Potential miRNAs-like molecules predicted in this work originated from precursors that form hairpin structures. During the formation of a mature miRNA, precursor molecules generate hairpins that are subsequently processed by dicer to form a miRNA/miRNA* duplex (Bartel *et al.*, 2004). Previous reports demonstrated negative free folding energies of the precursors of PM-milR-M1 and PM-milR-M2 from *P. marneffei* of -17.86 kcal/mol and -23.88 kcal/mol, respectively (Lau *et al.*, 2013). In *A. flavus,* the values vary between -19.4 kcal/mol and -140.2 kcal/mol*,* and in *M. anisopliae* they vary between -20 kcal/mol and -105.32 kcal/mol (Bai *et al.*, 2015; Zhou *et al.*, 2012).In this context, the values of negative free folding energy described in the present work are in agreement to those described in fungi. Interestingly, we obtained sequences of microRNA-like precursors of similar size to those described for other fungi (Zhou *et al.*, 2012a,b). For example, in *C. neoformans* the sizes of the precursors of the miR1 and miR2 are approximately 70 nucleotides (Jiang *et al.*, 2012).

Additionally we found low MFEI and similar AMFE values in the sequences of the microRNA-like precursors. Although these indices have not been described for miRNA-like precursors present in fungi, we hypothesize that the observed differences may be a result of the lower base complementarity between the precursor sequences present in fungal genomes when compared to plants, where the complementarity of bases could be higher (Mishra *et al.*, 2015). In general, the data also demonstrate a higher amount of uracil in microRNA-like precursors, as already described in the literature (Zhang *et al.*, 2006).

The experimental validation of some like microRNAs-like molecules in this work allowed the characterization of sequences ranging in size from 26 to 57 bp. In other fungi, smaller sizes for mature microRNAs-like were seen, as for example in *N. crassa* (Lee *et al.*, 2010)*, C. neoformans* (Jiang *et al.*, 2012), and *P. marneffei* (Lau *et al.*, 2013), in which detection of microRNAs-like molecules was performed by northern blot. In this work, the poly(T) adaptor RT-PCR method (Balcells *et al.*, 2011) adds a polyA tail to the microRNA-like molecule, and possibly, this process confers a larger size to the mature microRNA-like.

In conclusion, our data demonstrate the conservation of proteins involved in the post-transcriptional mechanism of gene regulation in different species of the *Paracoccidioides* complex*.* The transcripts encoding these proteins were detected in the parasitic phase, and sequences of microRNAs-like molecules were seen to be conserved in *P. brasiliensis.* Furthermore, five micro-RNAs-like sequences were confirmed by qualitative RT-PCR. Hence, our results point to the ability of *Paracoccidioides brasiliensis* to produce microRNAs-like molecules. Several studies demonstrate the plasticity of fungi for surviving in different conditions. For example, during dimorphic transition, these pathogens alter the constitution of the cell wall and membrane, changing metabolic pathways for energy production (Felipe *et al.*, 2005; Nunes *et al.*, 2005; Bastos *et al.*, 2007; Rezende *et al.*, 2011). The molecular mechanisms that control these processes are still not fully elucidated, but the confirmation of the capacity of these fungi to produce microRNAs-like allow the investigation of function of these molecules in the regulation of biological processes essential for the survival of these pathogens under different conditions, such as those found in the host, or between the different morphological fungal stages.

## Supplementary material

The following online material is available for this article:

Figure S1 Flowchart of study design.

Figure S2 Phylogenetic tree of the dicer and argonaute proteins.

Figure S3 Aligment analysis of miRNAs-like in the genome of *P. brasilensis*.

Table S1 Access numbers of the proteins used to construct the phylogenetic trees

Table S2 Oligonucleotide sequences used in the present study.

Table S3 miRNAs-like homologs to other fungi species identified in the genome of *P. brasiliensis Pb*18.

Table S4 Identification of miRNAs-like described in fungi vesicles with identity in the genome of *P. brasiliensis Pb*18.
