# Supplementary material for: In silico characterization of microRNAs-like sequences in the genome of Paracoccidioides brasiliensis
Source: Genet Mol Biol. 2019 Feb 14;42(1):95–107. doi: 10.1590/1678-4685-GMB-2018-0014 (PMC6428129; doi:10.1590/1678-4685-GMB-2018-0014)
Supplement: Supplementary file 1 [file 1415-4757-GMB-1678-4685-GMB-2018-0014-s004.pdf]

Supplementary Material "*In silico* characterization of microRNAs-like sequences in the genome of *Paracoccidioides brasiliensis*"

Table S1 - Access numbers of the proteins used to construct the phylogenetic trees.

	Access number			
	**Argonaute 1**	**Argonaute 2**	**Dicer 1**	**Dicer 2**
Aspergillus clavatus NRRL1	-	ACLA_058710	ACLA_014840	ACLA_055980
Aspergillus flavus AF70	-	-	KC686608	AFLA_066400
Aspergillus fumigatus	AFUA_3G11010	AFUA_8G05280	AFUA_5G11790	AFUA_4G02930
Aspergillus kawachii	-	-	-	AKAW_05119
Aspergillus nidulans	-	ANIA_01519	ANIA_00119	PDIP_77770
Aspergillus niger	-	ANI_1_1958144	ANI_1_424164	-
Aspergillus oryzae RIB40	AO090003000654	AO090012000881	AO090120000355	Ao3042_00077
Aspergillus terreus NIH2624	ATEG_04627	ATEG_04763	ATEG_02092	ATEG_07902
Blastomyces dermatitidis ATCC	BDDG_11934	BDDG_06070	BDCG_08125	BDCG_02544
Coccidioides immitis RS	CIMG_03797	CIMG_08296	-	CIMG_05654
Cryptococcus neoformans H99	-	-	CNAG_02745	-
Histoplasma capsulatum G186AR	HCBG_06692	HCBG_03944	HCBG_01751	HCBG_01136
Histoplasma capsulatum H143	HCDG_08528	HCDG_00823	HCDG_06891	-
Magnaporthe oryzae 70-15	-	-	MGG_01541	MGG_12357
Neurospora crassa OR74A	NCU06838	NCU08389	NCU08270	NCU06766
Paracoccidioides brasiliensis Pb01	PAAG_11422	PAAG_03231	PAAG_11489	PAAG_00072
Paracoccidioides brasiliensis Pb03	PADG_02302	PABG_00673	PABG_04917	PABG_05105
Paracoccidioides brasiliensis Pb18	PADG_00716	PADG_03108	PADG_11946	PADG_07189
Penicillium chrysogenum	EN45_032900	PC12G03410	-	-
Penicillium marneffei	-	AGM20448	-	-
Sclerotinia sclerotiorum	-	SS1G_00384	SS1G_13747	SS1G_10419
Schizosaccharomyces pombe 972h	-	-	SPCC188.13c	-
Sporothrix schenckii	-	-	C8CK81_SPOSC	-
