# Supplementary material for: In silico characterization of microRNAs-like sequences in the genome of Paracoccidioides brasiliensis
Source: Genet Mol Biol. 2019 Feb 14;42(1):95–107. doi: 10.1590/1678-4685-GMB-2018-0014 (PMC6428129; doi:10.1590/1678-4685-GMB-2018-0014)
Supplement: Supplementary file 2 [file 1415-4757-GMB-1678-4685-GMB-2018-0014-s005.pdf]

Supplementary Material "*In silico* characterization of microRNAs-like sequences in the genome of *Paracoccidioides brasiliensis*"

Table S2 - Oligonucleotide sequences used in the present study

Genes	Forward sequences	Reverse sequence
dcr 1	GGAGATTGAAGCTACTGAGAC	TCTGGCAGACACTATTTACAAC
dcr 2	GAGGGAGGCAACCAACTATC	TTAGAAACCACCTCGTCCTTG
ago-1	CCATGGCTGCAGTGTCAGTA	AAACACCATCGCGGAAGTAGT
ago-2	CGACTATTTCAGACGCACATAT	GGGTTAAGCTTAGCATTTGCC
Act	CGTCCTCGCCATCATGGTAT	TCTCCATATCATCCCAGTTCG
Super.:2.3:1128222-1128358(-)	GCAGTGTGCATGTGCAT	GGTCCAGTTTTTTTTTTTTTTTCAC
Super.:2.5:587199-587317(+)	CGCAGAAATCACCTTCAC	GGTCCAGTTTTTTTTTTTTTTTGAAG
Super.:2.6:955763-955879(+)	GGCGGACGCGATG	GTCCAGTTTTTTTTTTTTTTTCCAC
Super.:2.12:21200-21275(-)	CAGCGCAGTAGGATTAGGAT	GGTCCAGTTTTTTTTTTTTTTTCCT
Super.:2.14:171583-171670(+)	GGAGAGGGGGCCG	GGTCCAGTTTTTTTTTTTTTTTCAAC

(*) all primers described in Table S2 were constructed for this work, except the actin primer.
