# Supplementary material for: In silico characterization of microRNAs-like sequences in the genome of Paracoccidioides brasiliensis
Source: Genet Mol Biol. 2019 Feb 14;42(1):95–107. doi: 10.1590/1678-4685-GMB-2018-0014 (PMC6428129; doi:10.1590/1678-4685-GMB-2018-0014)
Supplement: Supplementary file 4 [file 1415-4757-GMB-1678-4685-GMB-2018-0014-s002.pdf]

Supplementary Material "*In silico* characterization of microRNAs-like sequences in the genome of *Paracoccidioides brasiliensis*"

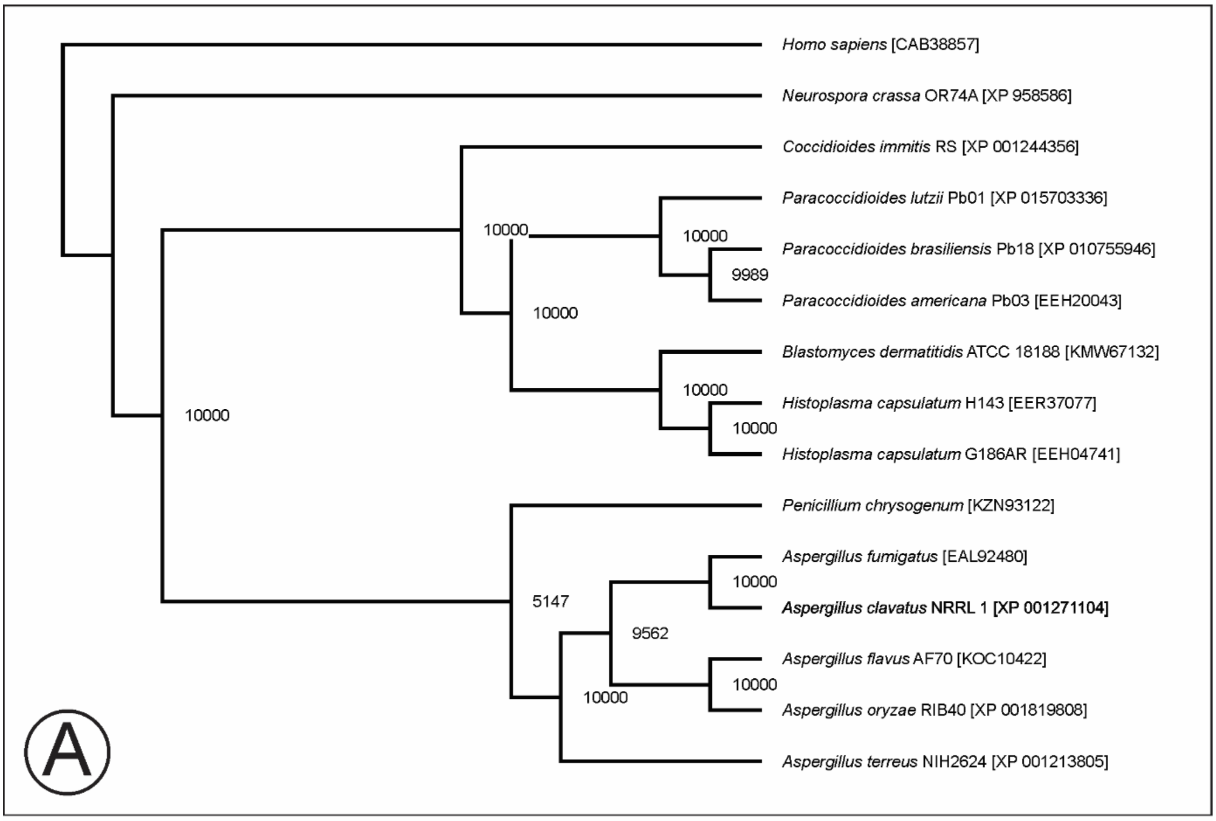

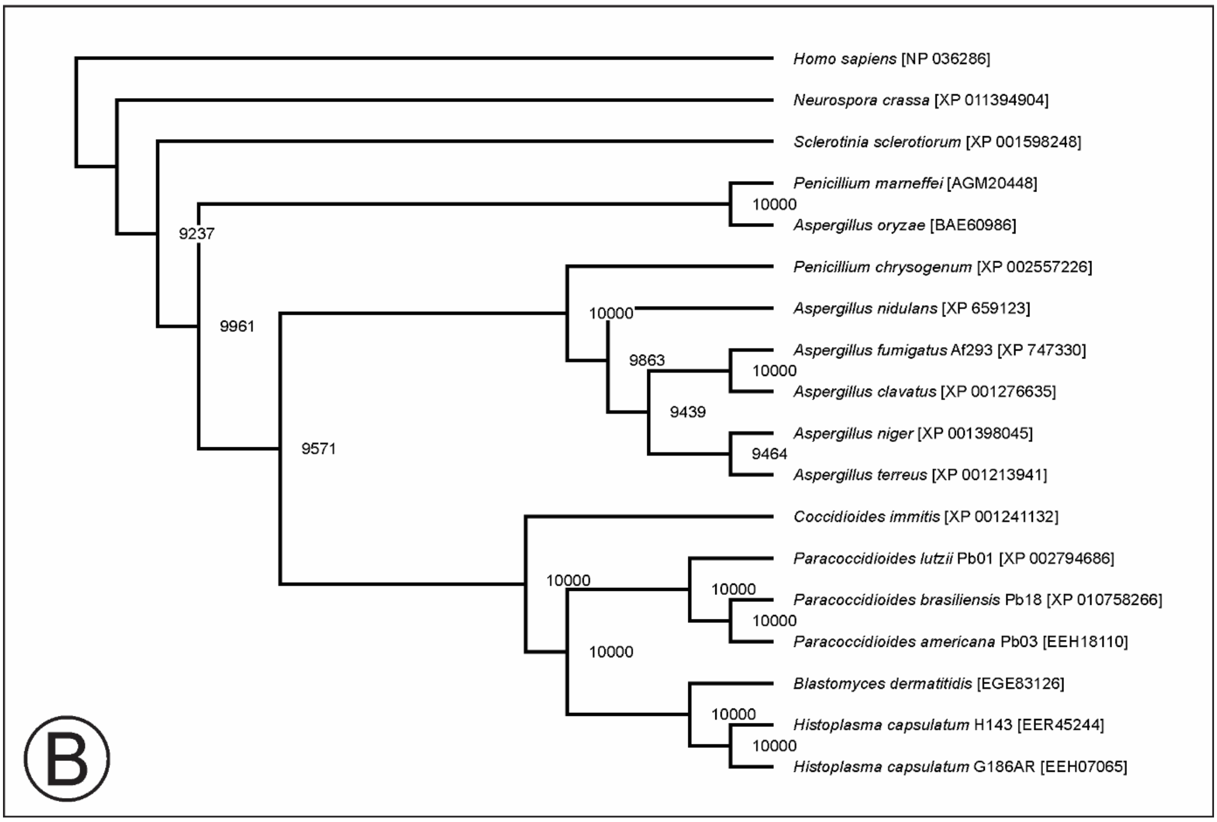

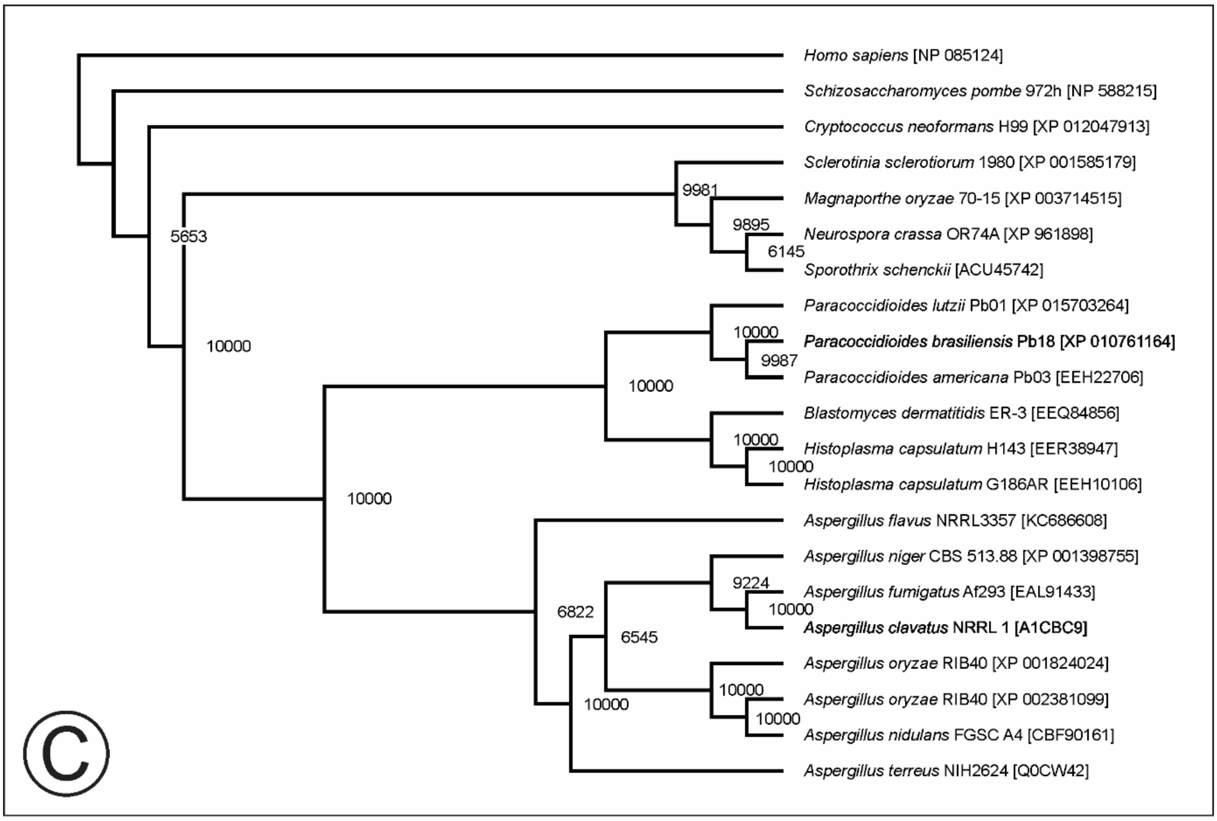

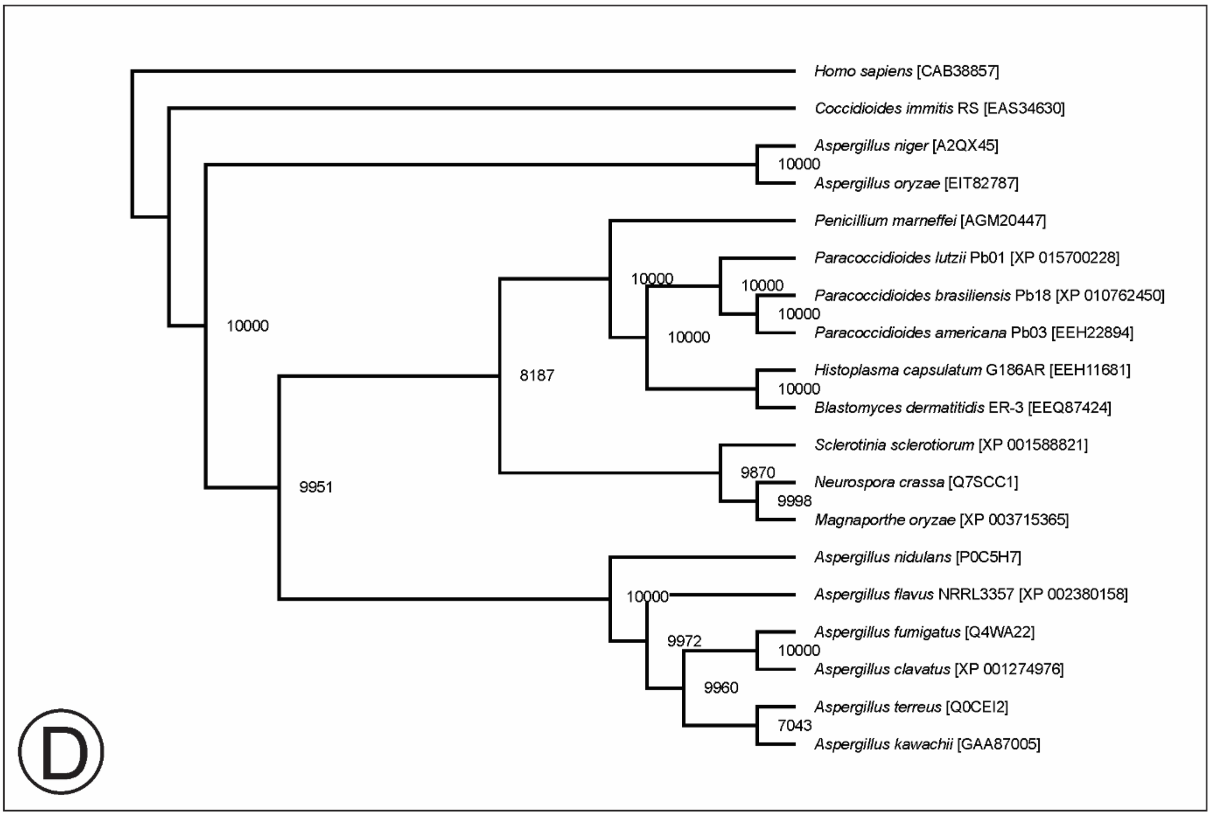

Figure S2 - Phylogenetic tree of the dicer and argonaute proteins from *P. brasiliensis* (*Pb*18), *P. americana* (*Pb03*) and *P. lutzii* (*Pb*01). (A) Argonaute-1p; (B) Argonaute-2p; (C) Dicer-1p; (D) Dicer-2p. The Phylogenetic tree was constructed by multiple sequence alignments using CLUSTALX2 and was generated by neighbor-joining method.
