# Supplementary material for: In silico characterization of microRNAs-like sequences in the genome of Paracoccidioides brasiliensis
Source: Genet Mol Biol. 2019 Feb 14;42(1):95–107. doi: 10.1590/1678-4685-GMB-2018-0014 (PMC6428129; doi:10.1590/1678-4685-GMB-2018-0014)
Supplement: Supplementary file 5 [file 1415-4757-GMB-1678-4685-GMB-2018-0014-s006.pdf]

Supplementary Material "*In silico* characterization of microRNAs-like sequences in the genome of *Paracoccidioides brasiliensis*"

Table S3 - miRNAs-like homologs to other fungi species identified in the genome of *Paracoccidioides brasiliensis Pb*18.

Query ID[a]	Sequence[b]	Subject ID[c]	Identity (%)[d]	Alignment length[e]	Mismatches[f]	Gap opens[g]	E-value[h]	Bit score[i]	Organism[j]	Reference[k]
Afl-milR-13	CUUUCUGGUUGAUGGCGUCGGA	Supercontig_2.7	100	16	0	0	0.048	32.2	*A.flavus*	Bai *et al.*. 2015
Afl-milR-23	GAGGGGAGAGGGGGCCGUUG	Supercontig_2.14	100	17	0	0	0.009	34.2	*A.flavus*	
Afl-milR-23	GAGGGGAGAGGGGGCCGUUG	Supercontig_2.1	95	20	1	0	0.036	32.2	*A.flavus*	
Afl-milR-33	GGCGAGAUGGCCGAGCGGUC	Supercontig_2.3	100	16	0	0	0.036	32.2	*A.flavus*	
Afl-milR-33	GGCGAGAUGGCCGAGCGGUC	Supercontig_2.9	100	17	0	0	0.009	34.2	*A.flavus*	
Afl-milR-6	GAGGGCGGAGAGGGGUGGAA	Supercontig_2.14	100	16	0	0	0.036	32.2	*A.flavus*	
Afl-milR-7	GUGGGAGGUUGAGUGGGUGGUA	Supercontig_2.3	100	16	0	0	0.048	32.2	*A.flavus*	
Afl-milR-9	GGCGAGAUGGCCGAGCGGUCC	Supercontig_2.3	100	17	0	0	0.011	34.2	*A.flavus*	
Afl-milR-9	GGCGAGAUGGCCGAGCGGUCC	Supercontig_2.9	100	18	0	0	0.003	36.2	*A.flavus*	
fox_milRNA_2	GACAACGUGGCCGAGUGGUUAAGGC	Supercontig_2.2	100	18	0	0	0.004	36.2	*F. oxysporum*	Chen *et al.*. 2014
fox_milRNA_2	GACAACGUGGCCGAGUGGUUAAGGC	Supercontig_2.6	100	18	0	0	0.004	36.2	*F. oxysporum*	
fox_milRNA_4	UGGAUGAAUCAAGCGUGGUAUGA	Supercontig_2.9	100	16	0	0	0.054	32.2	*F. oxysporum*	
fox_milRNA_8	CUUGAGACCCGGGUUCAAUUCCCGGC	Supercontig_2.8	95.65	23	1	0	0.001	38.2	*F. oxysporum*	
man-milR-10	AGGGAUCUAGAAAAGAAGGCUU	Supercontig_2.8	100	16	0	0	0.048	32.2	*M. anisopliae*	Zhou *et al.*. 2012
man-milR-14	UUAAAGAUGUGGAAAAGAAGGC	Supercontig_2.3	100	17	0	0	0.012	34.2	*M. anisopliae*	
man-milR-5	UUAACAAGGCGUCGAGGGAUA	Supercontig_2.1	100	16	0	0	0.042	32.2	*M. anisopliae*	
milR-18	TTGGCAGGAATGTGGTGGACGAACA	Supercontig_2.3	100	17	0	0	0.017	34.2	*N. crassa*	Yang *et al.*. 2013
milR-1	TAAGCCGCGAGTACGCCTCCGGACT	Supercontig_2.3	100	16	0	0	0.066	32.2	*N. crassa*	
milR-4	TTCCCAAATCACCTTCACCTTCACC	Supercontig_2.12	100	17	0	0	0.017	34.2	*N. crassa*	
milR-4	TTCCCAAATCACCTTCACCTTCACC	Supercontig_2.5	100	18	0	0	0.004	36.2	*N. crassa*	
milR-6	GGCCGATTTCCTCGTCAGTCA	Supercontig_2.1	100	16	0	0	0.042	32.2	*N. crassa*	
PM-milR-MC17	UGGCGGACGCGAUGGUGGAGG	Supercontig_2.6	100	16	0	0	0.042	32.2	*P. marneffei*	Lau *et al.*. 2013
PM-milR-MC7	UAGGAUUAGGAUUAGGAUUA	Supercontig_2.1	100	16	0	0	0.036	32.2	*P. marneffei*	
PM-milR-MC7	UAGGAUUAGGAUUAGGAUUA	Supercontig_2.12	100	16	0	0	0.036	32.2	*P. marneffei*	
PM-milR-MC7	UAGGAUUAGGAUUAGGAUUA	Supercontig_2.12	100	16	0	0	0.036	32.2	*P. marneffei*	
PM-milR-MC7	UAGGAUUAGGAUUAGGAUUA	Supercontig_2.12	100	17	0	0	0.009	34.2	*P. marneffei*	
PM-milR-MC7	UAGGAUUAGGAUUAGGAUUA	Supercontig_2.2	95	20	1	0	0.036	32.2	*P. marneffei*	
PM-milR-MC7	UAGGAUUAGGAUUAGGAUUA	Supercontig_2.4	100	17	0	0	0.009	34.2	*P. marneffei*	
PM-milR-MC7	UAGGAUUAGGAUUAGGAUUA	Supercontig_2.5	100	16	0	0	0.036	32.2	*P. marneffei*	
PM-milR-MC7	UAGGAUUAGGAUUAGGAUUA	Supercontig_2.5	100	17	0	0	0.009	34.2	*P. marneffei*	
PM-milR-MC7	UAGGAUUAGGAUUAGGAUUA	Supercontig_2.5	95	20	1	0	0.036	32.2	*P. marneffei*	
PM-milR-YC1	UGCCAUUGCUAAGUCAAGG	Supercontig_2.3	100	17	0	0	0.008	34.2	*P. marneffei*	
Tre-milR-2	TCTCTGTTGGAGTTGAGGGGG	Supercontig_2.2	100	16	0	0	0.042	32.2	*T. reesei*	Kang *et al.*. 2013
miR1	TCCTGAACTTGATCACCATTGA	-	-	-	-	-	-	-	*Cryptococcus neoformans*	Jiang *et al.*. 2012
miR2	TCGACACCTTCATCGTAA	-	-	-	-	-	-	-	*Cryptococcus neoformans*	
Afl-milR-4	GUGGAGGAUUGGGACGGGGU	-	-	-	-	-	-	-	*A.flavus*	

Query ID[a]	Sequence[b]	Subject ID[c]	Identity (%)[d]	Alignment length[e]	Mismatches[f]	Gap opens[g]	E-value[h]	Bit score[i]	Organism[j]	Reference[k]
Afl-milR-5	CUUUGGAGGGAUUGGUGGGA	-	-	-	-	-	-	-	*A.flavus*	
Afl-milR-8	UUUUGUGGAAUCUGCCUCGCGCU	-	-	-	-	-	-	-	*A.flavus*	
Afl-milR-10	CUGGAUUCGUCCCGGGAACCC	-	-	-	-	-	-	-	*A.flavus*	
Afl-milR-11	AGCGGUCUAAGGCGCACGGUUCA	-	-	-	-	-	-	-	*A.flavus*	
Afl-milR-12	CUUCUAUCUCGUCGGGGGUCA	-	-	-	-	-	-	-	*A.flavus*	
Afl-milR-14	AAAGGGGCAUGGGUAGUAUGA	-	-	-	-	-	-	-	*A.flavus*	
Afl-milR-15	CUGGUCGGGUUUGAUGAUGGA	-	-	-	-	-	-	-	*A.flavus*	
Afl-milR-16	AUUGCUUGCAUGUUCGUUCUGGA	-	-	-	-	-	-	-	*A.flavus*	
Afl-milR-17	CUUUCUCAUAUAUACGUCGGAA	-	-	-	-	-	-	-	*A.flavus*	
Afl-milR-18	CCAUGAUACUUUGUUGGUCGGA	-	-	-	-	-	-	-	*A.flavus*	
Afl-milR-19	AGCGAGACGACCUGCCUGGCA	-	-	-	-	-	-	-	*A.flavus*	
Afl-milR-20	CAUCUCUCUUGUCGGUUCGAGA	-	-	-	-	-	-	-	*A.flavus*	
Afl-milR-21	AUGGGGGUCUGUAUGUGAUGGA	-	-	-	-	-	-	-	*A.flavus*	
Afl-milR-22	GGAAGUUGAUCUUGAUUGUUGGA	-	-	-	-	-	-	-	*A.flavus*	
Afl-milR-24	UGGCUAUCGAUCGAUCGUCGGA	-	-	-	-	-	-	-	*A.flavus*	
Afl-milR-25	GUGUAGGGUGUGGUAGUGCUC	-	-	-	-	-	-	-	*A.flavus*	
Afl-milR-26	CCUGUCUAUCGAGGAUUGUUGGA	-	-	-	-	-	-	-	*A.flavus*	
Afl-milR-27	GGAUGAUAGGUCGGGAUGAGA	-	-	-	-	-	-	-	*A.flavus*	
Afl-milR-28	UAGCUAUCUCGUGACAGACAAU	-	-	-	-	-	-	-	*A.flavus*	
Afl-milR-29	ACUCCUUGGGCGCAUCGUUGGA	-	-	-	-	-	-	-	*A.flavus*	
Afl-milR-30	UUACUGUACAUAAGCUAGACA	-	-	-	-	-	-	-	*A.flavus*	
Afl-milR-31	CAAGGAUUGUGAUUGUUCUGGA	-	-	-	-	-	-	-	*A.flavus*	
Afl-milR-32	CUUGUUCGUCGGGGGGAUGGCA	-	-	-	-	-	-	-	*A.flavus*	
Afl-milR-34	AAGGCGGACGUUGGCGGCUGAUA	-	-	-	-	-	-	-	*A.flavus*	
Afl-milR-35	GGCGGACUGCUUCAAACGACGGA	-	-	-	-	-	-	-	*A.flavus*	
Afl-milR-36	ACUUUAGAUGGUCGUGUUGGGGA	-	-	-	-	-	-	-	*A.flavus*	
Afl-milR-37	CAUUGUUUAAGCGUCGUUGGAA	-	-	-	-	-	-	-	*A.flavus*	
Afl-milR-38	CCAUGGGAUUCUAAUCGUCGGA	-	-	-	-	-	-	-	*A.flavus*	
Afl-milR-39	GGUUUUGAUGAUCGUGUCGGA	-	-	-	-	-	-	-	*A.flavus*	
Afl-milR-40	CCUAUCGGCAUUGUGAGACGGA	-	-	-	-	-	-	-	*A.flavus*	
Afl-milR-41	GUCGUGGUAAGUGUGGCGUCGGA	-	-	-	-	-	-	-	*A.flavus*	
Afl-milR-42	GUUCUCAGCACGAUCGGCCGGA	-	-	-	-	-	-	-	*A.flavus*	
Afl-milR-43	CCUAGGACAAGGGCGCACAGAGU	-	-	-	-	-	-	-	*A.flavus*	
Afl-milR-44	GUAUCAAUGACAGAUCGUAAGGA	-	-	-	-	-	-	-	*A.flavus*	
Afl-milR-45	AGGCGACUGGUACAUGUAUGG	-	-	-	-	-	-	-	*A.flavus*	
Afl-milR-46	AUGGCAUUGAAUCGGUCGGGA	-	-	-	-	-	-	-	*A.flavus*	
Afl-milR-47	AUGUAGUACUGCAUCGUUCUGGA	-	-	-	-	-	-	-	*A.flavus*	
Afl-milR-48	UGGGUGGUGGGGUGGCGAUGGCU	-	-	-	-	-	-	-	*A.flavus*	
fox_milRNA_1	UGCUAGGGUAGAGAAUUUUUGCAGGC	-	-	-	-	-	-	-	*F. oxysporum*	
fox_milRNA_2	GGCAUUGUGUUCGCACGCGUAGGUUCG	-	-	-	-	-	-	-	*F. oxysporum*	
fox_milRNA_3a	GGUGUGGUGUAUCGGUUUAUCAUUCCGC	-	-	-	-	-	-	-	*F. oxysporum*	
fox_milRNA_3b	CCGGUGUGGUGUAUCGGUUAUCAUUCCGC	-	-	-	-	-	-	-	*F. oxysporum*	
fox_milRNA_5	UCCGGUAUGGUGUAGUGGC	-	-	-	-	-	-	-	*F. oxysporum*	
fox_milRNA_6	GUUCCGUGGUCUAGUUGGUUAUGGCAUCU	-	-	-	-	-	-	-	*F. oxysporum*	
fox_milRNA_7	CUUCCGUAGUAUAGUGGUCAGUAUGCA	-	-	-	-	-	-	-	*F. oxysporum*	
man-milR-1	UAUCUUGUGGACUAAUAGGUA	-	-	-	-	-	-	-	*M. anisopliae*	

Query ID[a]	Sequence[b]	Subject ID[c]	Identity (%)[d]	Alignment length[e]	Mismatches[f]	Gap opens[g]	E-value[h]	Bit score[i]	Organism[j]	Reference[k]
man-milR-2	UACAAGGGCACGAGCAAGGU	-	-	-	-	-	-	-	*M. anisopliae*	
man-milR-3	UUGUCGAGGCAUACCACUAUU	-	-	-	-	-	-	-	*M. anisopliae*	
man-milR-4	UCGAGGAGCAGAAGCUGAUCUU	-	-	-	-	-	-	-	*M. anisopliae*	
man-milR-6	UUUGGAGAGGCUGCUGUGUUU	-	-	-	-	-	-	-	*M. anisopliae*	
man-milR-7	GCGGGUGCUGAGAAAGCGUUUA	-	-	-	-	-	-	-	*M. anisopliae*	
man-milR-8	UUGCAUGAUGAGACUUAUUU	-	-	-	-	-	-	-	*M. anisopliae*	
man-milR-9	UGCCUAGGCAGGUAGAUCAG	-	-	-	-	-	-	-	*M. anisopliae*	
man-milR-11	UGCAGGGGAGCAUCGUCGUUG	-	-	-	-	-	-	-	*M. anisopliae*	
man-milR-12	AGACCUCGUUGAUGCUGGCAUU	-	-	-	-	-	-	-	*M. anisopliae*	
man-milR-13	CGACGACUCUGGCGAGGACAA	-	-	-	-	-	-	-	*M. anisopliae*	
man-milR-15	GAUGUCGAAUCGUGCCGGGGCUC	-	-	-	-	-	-	-	*M. anisopliae*	
milR-2	TGTCCTGGTACTTTCATCCGGCTGT	-	-	-	-	-	-	-	*N. crassa*	
milR-3	TGAACGAGCGGTTGTTGT	-	-	-	-	-	-	-	*N. crassa*	
milR-5	AAGCAAGATCCGGACATTC	-	-	-	-	-	-	-	*N. crassa*	
milR-7	TGCGGTGCTTTCCTTGCCCT	-	-	-	-	-	-	-	*N. crassa*	
milR-8	AAACGCTCTACCGGGTTGCTGTTAGA	-	-	-	-	-	-	-	*N. crassa*	
milR-9	GAGCTTGTCGCCTTCAA	-	-	-	-	-	-	-	*N. crassa*	
milR-10	CACTCGTGTGCTGTAGCGGGACTA	-	-	-	-	-	-	-	*N. crassa*	
milR-11	TGACCGGGCGGTTGTTGTT	-	-	-	-	-	-	-	*N. crassa*	
milR-12	TCATCCGATCATGTCACATTGTC	-	-	-	-	-	-	-	*N. crassa*	
milR-13	TGGGAATGTGAGGCGTTTG	-	-	-	-	-	-	-	*N. crassa*	
milR-14	TGCCGTGTCGTAGCATCGAAGTCAA	-	-	-	-	-	-	-	*N. crassa*	
milR-15	TTATTTGCCAAATCAACACTCGG	-	-	-	-	-	-	-	*N. crassa*	
milR-16	TACTTGATTCACAGCGTCTATAC	-	-	-	-	-	-	-	*N. crassa*	
milR-17	CGGCATCAGCGTGTTCA	-	-	-	-	-	-	-	*N. crassa*	
milR-19	GATACACGCTAGATGTCCCT	-	-	-	-	-	-	-	*N. crassa*	
milR-20	TACTCTGTGTGCTCGGTGTGGTT	-	-	-	-	-	-	-	*N. crassa*	
milR--21	ATCCTGGCTTCCTTTGCCTG	-	-	-	-	-	-	-	*N. crassa*	
milR-22	TGGAGGGTGATATATGGTTTGCCAG	-	-	-	-	-	-	-	*N. crassa*	
milR-23	TGCAATCTAGTCGCGTTCCCTCTT	-	-	-	-	-	-	-	*N. crassa*	
milR-24	TGCGGGGCATTGAGTGGTCGACG	-	-	-	-	-	-	-	*N. crassa*	
milR-25	CGACGCGGAGAAGAAGGGGATTG	-	-	-	-	-	-	-	*N. crassa*	
PM-milR-M1	GAGAAACGCCUUAUGAUCGAC	-	-	-	-	-	-	-	*P. marneffei*	
PM-milR-M1*	UGACUCGAAGAGCCUCUA	-	-	-	-	-	-	-	*P. marneffei*	
PM-milR-M2	GUCCUAUAGUAAAGCCAGUC	-	-	-	-	-	-	-	*P. marneffei*	
PM-milR-M2*	AUUUCUAGGCUAUAAAAGCUU	-	-	-	-	-	-	-	*P. marneffei*	
PM-milR-MC3	UGAUAUCAAAGUGGGCUAUC	-	-	-	-	-	-	-	*P. marneffei*	
PM-milR-MC4	UCAAGUCAACCCUUACUC	-	-	-	-	-	-	-	*P. marneffei*	
PM-milR-MC5	UUGCUAUGAUGAAAGCUGAGCA	-	-	-	-	-	-	-	*P. marneffei*	
PM-milR-MC6	AACGUUUAAAUUUCCGAUACAAUU	-	-	-	-	-	-	-	*P. marneffei*	
PM-milR-MC8	UUUCUACAGCUGCUGAACGUC	-	-	-	-	-	-	-	*P. marneffei*	
PM-milR-MC9	UUGGCGUUGGGUGUAAUUG	-	-	-	-	-	-	-	*P. marneffei*	
PM-milR-MC10	UCGACUGGCUCACCUGAUGCC	-	-	-	-	-	-	-	*P. marneffei*	
PM-milR-MC11	UCGAUGUACUUCCUUGUGGA	-	-	-	-	-	-	-	*P. marneffei*	
PM-milR-MC12	UGUUCAUCGAUCUGCUGUAGA	-	-	-	-	-	-	-	*P. marneffei*	
PM-milR-MC13	UGCCACUCGAUCAUCUUGGG	-	-	-	-	-	-	-	*P. marneffei*	

**Query ID[a]**	**Sequence[b]**	**Subject ID[c]**	**Identity (%)[d]**	**Alignment length[e]**	**Mismatches[f]**	**Gap opens[g]**	**E-value[h]**	**Bit score[i]**	**Organism[j]**	**Reference[k]**
PM-milR-MC14	UAAGAGCUGUACAUAUGUAAG	-	-	-	-	-	-	-	*P. marneffei*	
PM-milR-MC15	AUCCGGAUCGAGUUAUUCAC	-	-	-	-	-	-	-	*P. marneffei*	
PM-milR-MC16	CAUAAGGUCGAGAGUCUCGCA	-	-	-	-	-	-	-	*P. marneffei*	
PM-milR-YC2	CAGCGGUGAUGACAACC	-	-	-	-	-	-	-	*P. marneffei*	
PM-milR-YC3	CCGCUUCUAAAAUUGCUAGAGC	-	-	-	-	-	-	-	*P. marneffei*	
PM-milR-YC4	UUGCUAUGAUGAAAGCUGAGCA	-	-	-	-	-	-	-	*P. marneffei*	
PM-milR-YC5	UUUCUUGUCUACCUUUCGAGU	-	-	-	-	-	-	-	*P. marneffei*	
PM-milR-YC6	UUCUCGGUGGCGAUGUCCAUU	-	-	-	-	-	-	-	*P. marneffei*	
PM-milR-YC7	CCUUCAGAUCUGGGCUAUGCCC	-	-	-	-	-	-	-	*P. marneffei*	
Tre-milR-1	AGCCGGCTGTTGACGTAGGTGA	-	-	-	-	-	-	-	*T. reesei*	
Tre-milR-3	GGGAGAATGCGCCGTGATTGT	-	-	-	-	-	-	-	*T. reesei*	
Tre-milR-4	AGCAGCGACGGCGGAACTCTGC	-	-	-	-	-	-	-	*T. reesei*	
Tre-milR-5	CCCGTTTATCTGATCAACGCCG	-	-	-	-	-	-	-	*T. reesei*	
Tre-milR-6	CGGAGCTGGAGGAGGACTGCGA	-	-	-	-	-	-	-	*T. reesei*	
Tre-milR-7	TCAAGGGGAATCTGAGGCAG	-	-	-	-	-	-	-	*T. reesei*	
Tre-milR-8	CTCGAGGGAAGTGGAGATGGA	-	-	-	-	-	-	-	*T. reesei*	
Tre-milR-9	TGGCATGTTAGACAAGTTGCG	-	-	-	-	-	-	-	*T. reesei*	
Tre-milR-10	AGGCTGTACTGTAGGGCAG	-	-	-	-	-	-	-	*T. reesei*	
Tre-milR-11	TGGAGACGTGGAGCCGGA	-	-	-	-	-	-	-	*T. reesei*	
Tre-milR-12	GGTGCGGGCTGGCGGCGG	-	-	-	-	-	-	-	*T. reesei*	
Tre-milR-13	CCAGCAGGACTATGACGACG	-	-	-	-	-	-	-	*T. reesei*	

a) Identifier of the miRNAs-like;
b) Sequence;
c) Alignment region;
d) Percent of the identity between the sequences;
e) Size of alignment between the sequences of miRNAs-like and the genome region of *P. brasiliensis* (*Pb*18);
f) Number of different bases between the aligned sequences;
g) Absence of complementary bases between the sequences;
h) Likelihood sequence analyzed to align randomly to other sequences in the database. false positive rate;
i) Normalized score obtained using the following formula: S'= (I S – ln K)/ln 2;
j) Microorganism which miRNA-like have been described;
k) Articles describing miRNAs-like in fungus;
l) (-) Absence of alignment in genome of *P.brasiliensis* (*Pb*18);
m) (*) microRNA star.
