# Supplementary material for: In silico characterization of microRNAs-like sequences in the genome of Paracoccidioides brasiliensis
Source: Genet Mol Biol. 2019 Feb 14;42(1):95–107. doi: 10.1590/1678-4685-GMB-2018-0014 (PMC6428129; doi:10.1590/1678-4685-GMB-2018-0014)
Supplement: Supplementary file 6 [file 1415-4757-GMB-1678-4685-GMB-2018-0014-s003.pdf]

Supplementary Material "*In silico* characterization of microRNAs-like sequences in the genome of *Paracoccidioides brasiliensis*"

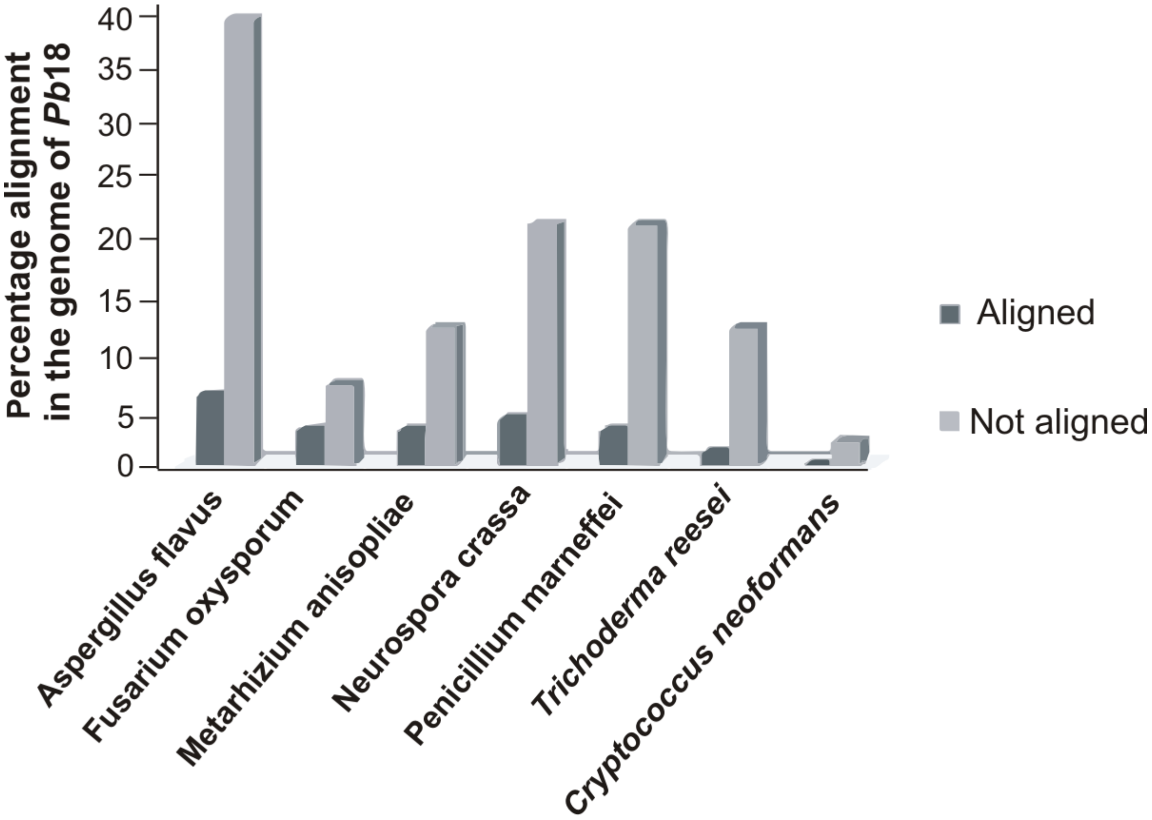

Figure S3 - Aligment analysis of miRNAs-like in the genome of *P. brasilensis*. MiRNAs-like described in fungi demonstrated identity to regions of the *P. brasiliensis Pb*18 genome. The dark gray bars represent the percentage of miRNAs-like aligned in the genome of *P. brasiliensis* and light gray bars demonstrate the percentage of miRNAs-like that did not align to the *Pb*18 genome.
