# Supplementary material for: In silico characterization of microRNAs-like sequences in the genome of Paracoccidioides brasiliensis
Source: Genet Mol Biol. 2019 Feb 14;42(1):95–107. doi: 10.1590/1678-4685-GMB-2018-0014 (PMC6428129; doi:10.1590/1678-4685-GMB-2018-0014)
Supplement: Supplementary file 7 [file 1415-4757-GMB-1678-4685-GMB-2018-0014-s007.pdf]

Supplementary Material "*In silico* characterization of microRNAs-like sequences in the genome of *Paracoccidioides brasiliensis*"

Table S4 - Identification of miRNAs-like described in fungi vesicles with identity in the genome of *P. brasiliensis Pb*18.

Query id[a]	Subject id[b]	% Identity[c]	Alignment length[d]	Mismatches[e]	Gap opens[f]	Evalue [g]	Bit score[h]	Fungi vesicle[i]	Reference[j]
aae-miR-309b-5p	Supercontig_2.3	100	16	0	0	0.054	32.2	*S. cerevisiae*	Peres da Silva *et.al.* 2015
aca-miR-1388-5p	Supercontig_2.4	95	20	1	0	0.048	32.2	S. cerevisiae/ *C. albicans*	
aca-miR-20a-5p	Supercontig_2.3	100	17	0	0	0.012	34.2	*C. albicans*	
aca-miR-5419	Supercontig_2.1	100	16	0	0	0.042	32.2	*C.neoformans/S. cerevisiae*	
aly-miR166g-5p	Supercontig_2.4	100	16	0	0	0.042	32.2	*C.neoformans*	
aly-miR166g-5p	Supercontig_2.9	100	16	0	0	0.042	32.2	*C.neoformans*	
aly-miR167b-3p*	Supercontig_2.8	100	16	0	0	0.054	32.2	*S. cerevisiae*	
aly-miR4233	Supercontig_2.1	100	17	0	0	0.011	34.2	*C. albicans*	
aly-miR4233	Supercontig_2.2	100	16	0	0	0.042	32.2	*C. albicans*	
aly-miR4233	Supercontig_2.29	100	16	0	0	0.042	32.2	*C. albicans*	
aly-miR4233	Supercontig_2.4	100	16	0	0	0.042	32.2	*C. albicans*	
aly-miR4233	Supercontig_2.8	100	16	0	0	0.042	32.2	*C. albicans*	
ame-miR-3754	Supercontig_2.2	100	16	0	0	0.054	32.2	*P. brasiliensis*	
ame-miR-3772	Supercontig_2.2	100	16	0	0	0.054	32.2	*C. albicans/ C.neoformans*	
ath-miR2938	Supercontig_2.7	100	16	0	0	0.042	32.2	*C. albicans*	
ath-miR414	Supercontig_2.10	100	17	0	0	0.011	34.2	*C. albicans/ P. brasiliensis*	
ath-miR414	Supercontig_2.1	100	16	0	0	0.042	32.2	*C. albicans/ P. brasiliensis*	
ath-miR414	Supercontig_2.11	95.24	21	1	0	0.011	34.2	*C. albicans/ P. brasiliensis*	
ath-miR414	Supercontig_2.11	95.24	21	1	0	0.011	34.2	*C. albicans/ P. brasiliensis*	
ath-miR414	Supercontig_2.11	95.24	21	1	0	0.011	34.2	*C. albicans/ P. brasiliensis*	
ath-miR414	Supercontig_2.13	95.24	21	1	0	0.011	34.2	*C. albicans/ P. brasiliensis*	
ath-miR414	Supercontig_2.14	100	17	0	0	0.011	34.2	*C. albicans/ P. brasiliensis*	
ath-miR414	Supercontig_2.1	95	20	1	0	0.042	32.2	*C. albicans/ P. brasiliensis*	
ath-miR414	Supercontig_2.1	95	20	1	0	4.20E-02	32.2	*C. albicans/ P. brasiliensis*	
ath-miR414	Supercontig_2.1	95	20	1	0	0.042	32.2	*C. albicans/ P. brasiliensis*	
ath-miR5021	Supercontig_2.10	100	16	0	0	0.036	32.2	*S. cerevisiae*	
ath-miR5021	Supercontig_2.10	100	16	0	0	0.036	32.2	*S. cerevisiae*	
ath-miR5021	Supercontig_2.10	100	16	0	0	0.036	32.2	*S. cerevisiae*	
ath-miR5021	Supercontig_2.10	100	16	0	0	0.036	32.2	*S. cerevisiae*	
ath-miR5021	Supercontig_2.10	100	16	0	0	0.036	32.2	*S. cerevisiae*	
ath-miR5021	Supercontig_2.10	100	16	0	0	0.036	32.2	*S. cerevisiae*	
ath-miR5021	Supercontig_2.10	100	16	0	0	0.036	32.2	*S. cerevisiae*	
ath-miR5021	Supercontig_2.10	100	16	0	0	0.036	32.2	*S. cerevisiae*	
ath-miR5021	Supercontig_2.10	100	16	0	0	3.60E-02	32.2	*S. cerevisiae*	
ath-miR5021	Supercontig_2.10	100	16	0	0	0.036	32.2	*S. cerevisiae*	
ath-miR5021	Supercontig_2.10	100	16	0	0	0.036	32.2	*S. cerevisiae*	
ath-miR5021	Supercontig_2.10	100	16	0	0	0.036	32.2	*S. cerevisiae*	
ath-miR5021	Supercontig_2.10	100	16	0	0	0.036	32.2	*S. cerevisiae*	
ath-miR5021	Supercontig_2.10	100	16	0	0	0.036	32.2	*S. cerevisiae*	
ath-miR5021	Supercontig_2.10	100	16	0	0	0.036	32.2	*S. cerevisiae*	

Query id[a]	Subject id[b]	% Identity[c]	Alignment length[d]	Mismatches[e]	Gap opens[f]	Evalue [g]	Bit score[h]	Fungi vesicle[i]	Reference[j]
ath-miR5021	Supercontig_2.10	100	16	0	0	0.036	32.2	*S. cerevisiae*	
ath-miR5021	Supercontig_2.10	100	16	0	0	0.036	32.2	*S. cerevisiae*	
ath-miR5021	Supercontig_2.10	100	16	0	0	0.036	32.2	*S. cerevisiae*	
ath-miR5021	Supercontig_2.10	100	16	0	0	0.036	32.2	*S. cerevisiae*	
ath-miR5021	Supercontig_2.10	100	16	0	0	0.036	32.2	*S. cerevisiae*	
ath-miR5021	Supercontig_2.10	100	16	0	0	0.036	32.2	*S. cerevisiae*	
ath-miR5021	Supercontig_2.10	100	16	0	0	0.036	32.2	*S. cerevisiae*	
ath-miR5021	Supercontig_2.10	100	16	0	0	0.036	32.2	*S. cerevisiae*	
ath-miR5021	Supercontig_2.10	100	16	0	0	0.036	32.2	*S. cerevisiae*	
ath-miR5021	Supercontig_2.10	100	16	0	0	0.036	32.2	*S. cerevisiae*	
ath-miR5021	Supercontig_2.10	100	16	0	0	0.036	32.2	*S. cerevisiae*	
ath-miR5021	Supercontig_2.10	100	16	0	0	0.036	32.2	*S. cerevisiae*	
ath-miR5021	Supercontig_2.10	100	16	0	0	0.036	32.2	*S. cerevisiae*	
ath-miR5021	Supercontig_2.10	100	16	0	0	0.036	32.2	*S. cerevisiae*	
ath-miR5021	Supercontig_2.10	100	16	0	0	0.036	32.2	*S. cerevisiae*	
ath-miR5021	Supercontig_2.10	100	16	0	0	0.036	32.2	*S. cerevisiae*	
ath-miR5021	Supercontig_2.10	100	16	0	0	0.036	32.2	*S. cerevisiae*	
ath-miR5021	Supercontig_2.10	100	16	0	0	0.036	32.2	*S. cerevisiae*	
ath-miR5021	Supercontig_2.10	100	16	0	0	0.036	32.2	*S. cerevisiae*	
ath-miR5021	Supercontig_2.10	100	16	0	0	0.036	32.2	*S. cerevisiae*	
ath-miR5021	Supercontig_2.10	100	16	0	0	0.036	32.2	*S. cerevisiae*	
ath-miR5021	Supercontig_2.10	100	16	0	0	3.60E-02	32.2	*S. cerevisiae*	
ath-miR5021	Supercontig_2.10	100	16	0	0	0.036	32.2	*S. cerevisiae*	
ath-miR5021	Supercontig_2.10	100	16	0	0	0.036	32.2	*S. cerevisiae*	
ath-miR5021	Supercontig_2.10	100	16	0	0	0.036	32.2	*S. cerevisiae*	
ath-miR5021	Supercontig_2.10	100	16	0	0	0.036	32.2	*S. cerevisiae*	
ath-miR5021	Supercontig_2.10	100	16	0	0	0.036	32.2	*S. cerevisiae*	
ath-miR5021	Supercontig_2.10	100	16	0	0	0.036	32.2	*S. cerevisiae*	
ath-miR5021	Supercontig_2.10	100	16	0	0	0.036	32.2	*S. cerevisiae*	
ath-miR5021	Supercontig_2.10	100	16	0	0	0.036	32.2	*S. cerevisiae*	
ath-miR5021	Supercontig_2.10	100	16	0	0	0.036	32.2	*S. cerevisiae*	
ath-miR5021	Supercontig_2.10	100	16	0	0	3.60E-02	32.2	*S. cerevisiae*	
ath-miR5021	Supercontig_2.10	100	16	0	0	0.036	32.2	*S. cerevisiae*	
ath-miR5021	Supercontig_2.10	100	17	0	0	0.009	34.2	*S. cerevisiae*	
ath-miR5021	Supercontig_2.10	100	17	0	0	0.009	34.2	*S. cerevisiae*	
ath-miR5021	Supercontig_2.10	100	17	0	0	0.009	34.2	*S. cerevisiae*	
ath-miR5021	Supercontig_2.10	100	17	0	0	0.009	34.2	*S. cerevisiae*	
ath-miR5021	Supercontig_2.10	100	18	0	0	0.002	36.2	*S. cerevisiae*	
ath-miR5021	Supercontig_2.10	100	18	0	0	0.002	36.2	*S. cerevisiae*	
ath-miR5021	Supercontig_2.1	100	16	0	0	0.036	32.2	*S. cerevisiae*	
ath-miR5021	Supercontig_2.1	100	16	0	0	0.036	32.2	*S. cerevisiae*	
ath-miR5021	Supercontig_2.1	100	16	0	0	0.036	32.2	*S. cerevisiae*	
ath-miR5021	Supercontig_2.1	100	16	0	0	0.036	32.2	*S. cerevisiae*	
ath-miR5021	Supercontig_2.1	100	16	0	0	0.036	32.2	*S. cerevisiae*	
ath-miR5021	Supercontig_2.1	100	16	0	0	0.036	32.2	*S. cerevisiae*	
ath-miR5021	Supercontig_2.1	100	16	0	0	0.036	32.2	*S. cerevisiae*	

Query id[a]	Subject id[b]	% Identity[c]	Alignment length[d]	Mismatches[e]	Gap opens[f]	Evalue [g]	Bit score[h]	Fungi vesicle[i]	Reference[j]
ath-miR5021	Supercontig_2.1	100	16	0	0	0.036	32.2	*S. cerevisiae*	
ath-miR5021	Supercontig_2.1	100	16	0	0	0.036	32.2	*S. cerevisiae*	
ath-miR5021	Supercontig_2.1	100	16	0	0	0.036	32.2	*S. cerevisiae*	
ath-miR5021	Supercontig_2.1	100	16	0	0	0.036	32.2	*S. cerevisiae*	
ath-miR5021	Supercontig_2.1	100	16	0	0	0.036	32.2	*S. cerevisiae*	
ath-miR5021	Supercontig_2.1	100	16	0	0	0.036	32.2	*S. cerevisiae*	
ath-miR5021	Supercontig_2.1	100	16	0	0	0.036	32.2	*S. cerevisiae*	
ath-miR5021	Supercontig_2.1	100	16	0	0	0.036	32.2	*S. cerevisiae*	
ath-miR5021	Supercontig_2.1	100	16	0	0	0.036	32.2	*S. cerevisiae*	
ath-miR5021	Supercontig_2.1	100	16	0	0	0.036	32.2	*S. cerevisiae*	
ath-miR5021	Supercontig_2.1	100	16	0	0	0.036	32.2	*S. cerevisiae*	
ath-miR5021	Supercontig_2.1	100	16	0	0	0.036	32.2	*S. cerevisiae*	
ath-miR5021	Supercontig_2.1	100	16	0	0	0.036	32.2	*S. cerevisiae*	
ath-miR5021	Supercontig_2.1	100	16	0	0	0.036	32.2	*S. cerevisiae*	
ath-miR5021	Supercontig_2.1	100	16	0	0	0.036	32.2	*S. cerevisiae*	
ath-miR5021	Supercontig_2.1	100	16	0	0	0.036	32.2	*S. cerevisiae*	
ath-miR5021	Supercontig_2.1	100	16	0	0	0.036	32.2	*S. cerevisiae*	
ath-miR5021	Supercontig_2.1	100	16	0	0	0.036	32.2	*S. cerevisiae*	
ath-miR5021	Supercontig_2.1	100	16	0	0	0.036	32.2	*S. cerevisiae*	
ath-miR5021	Supercontig_2.1	100	16	0	0	0.036	32.2	*S. cerevisiae*	
ath-miR5021	Supercontig_2.1	100	16	0	0	0.036	32.2	*S. cerevisiae*	
ath-miR5021	Supercontig_2.1	100	16	0	0	0.036	32.2	*S. cerevisiae*	
ath-miR5021	Supercontig_2.1	100	16	0	0	0.036	32.2	*S. cerevisiae*	
ath-miR5021	Supercontig_2.1	100	16	0	0	0.036	32.2	*S. cerevisiae*	
ath-miR5021	Supercontig_2.1	100	16	0	0	0.036	32.2	*S. cerevisiae*	
ath-miR5021	Supercontig_2.1	100	16	0	0	0.036	32.2	*S. cerevisiae*	
ath-miR5021	Supercontig_2.1	100	16	0	0	0.036	32.2	*S. cerevisiae*	
ath-miR5021	Supercontig_2.1	100	16	0	0	0.036	32.2	*S. cerevisiae*	
ath-miR5021	Supercontig_2.1	100	16	0	0	0.036	32.2	*S. cerevisiae*	
ath-miR5021	Supercontig_2.1	100	16	0	0	0.036	32.2	*S. cerevisiae*	
ath-miR5021	Supercontig_2.1	100	16	0	0	0.036	32.2	*S. cerevisiae*	
ath-miR5021	Supercontig_2.1	100	16	0	0	0.036	32.2	*S. cerevisiae*	
ath-miR5021	Supercontig_2.1	100	16	0	0	0.036	32.2	*S. cerevisiae*	
ath-miR5021	Supercontig_2.1	100	16	0	0	0.036	32.2	*S. cerevisiae*	
ath-miR5021	Supercontig_2.1	100	16	0	0	0.036	32.2	*S. cerevisiae*	
ath-miR5021	Supercontig_2.1	100	16	0	0	0.036	32.2	*S. cerevisiae*	
ath-miR5021	Supercontig_2.1	100	16	0	0	0.036	32.2	*S. cerevisiae*	
ath-miR5021	Supercontig_2.1	100	16	0	0	0.036	32.2	*S. cerevisiae*	
ath-miR5021	Supercontig_2.1	100	16	0	0	0.036	32.2	*S. cerevisiae*	
ath-miR5021	Supercontig_2.1	100	16	0	0	0.036	32.2	*S. cerevisiae*	
ath-miR5021	Supercontig_2.1	100	16	0	0	0.036	32.2	*S. cerevisiae*	
ath-miR5021	Supercontig_2.1	100	16	0	0	0.036	32.2	*S. cerevisiae*	
ath-miR5021	Supercontig_2.1	100	16	0	0	0.036	32.2	*S. cerevisiae*	
ath-miR5021	Supercontig_2.1	100	16	0	0	0.036	32.2	*S. cerevisiae*	
ath-miR5021	Supercontig_2.1	100	16	0	0	0.036	32.2	*S. cerevisiae*	
ath-miR5021	Supercontig_2.1	100	16	0	0	0.036	32.2	*S. cerevisiae*	

Query id[a]	Subject id[b]	% Identity[c]	Alignment length[d]	Mismatches[e]	Gap opens[f]	Evalue [g]	Bit score[h]	Fungi vesicle[i]	Reference[j]
ath-miR5021	Supercontig_2.1	100	16	0	0	0.036	32.2	*S. cerevisiae*	
ath-miR5021	Supercontig_2.1	100	16	0	0	0.036	32.2	*S. cerevisiae*	
ath-miR5021	Supercontig_2.1	100	16	0	0	0.036	32.2	*S. cerevisiae*	
ath-miR5021	Supercontig_2.1	100	16	0	0	0.036	32.2	*S. cerevisiae*	
ath-miR5021	Supercontig_2.1	100	16	0	0	0.036	32.2	*S. cerevisiae*	
ath-miR5021	Supercontig_2.1	100	16	0	0	0.036	32.2	*S. cerevisiae*	
ath-miR5021	Supercontig_2.1	100	16	0	0	0.036	32.2	*S. cerevisiae*	
ath-miR5021	Supercontig_2.1	100	16	0	0	0.036	32.2	*S. cerevisiae*	
ath-miR5021	Supercontig_2.1	100	16	0	0	0.036	32.2	*S. cerevisiae*	
ath-miR5021	Supercontig_2.1	100	16	0	0	0.036	32.2	*S. cerevisiae*	
ath-miR5021	Supercontig_2.1	100	16	0	0	0.036	32.2	*S. cerevisiae*	
ath-miR5021	Supercontig_2.1	100	16	0	0	0.036	32.2	*S. cerevisiae*	
ath-miR5021	Supercontig_2.1	100	16	0	0	0.036	32.2	*S. cerevisiae*	
ath-miR5021	Supercontig_2.1	100	16	0	0	0.036	32.2	*S. cerevisiae*	
ath-miR5021	Supercontig_2.1	100	16	0	0	0.036	32.2	*S. cerevisiae*	
ath-miR5021	Supercontig_2.1	100	16	0	0	0.036	32.2	*S. cerevisiae*	
ath-miR5021	Supercontig_2.1	100	16	0	0	0.036	32.2	*S. cerevisiae*	
ath-miR5021	Supercontig_2.1	100	16	0	0	0.036	32.2	*S. cerevisiae*	
ath-miR5021	Supercontig_2.1	100	16	0	0	0.036	32.2	*S. cerevisiae*	
ath-miR5021	Supercontig_2.1	100	16	0	0	0.036	32.2	*S. cerevisiae*	
ath-miR5021	Supercontig_2.1	100	16	0	0	0.036	32.2	*S. cerevisiae*	
ath-miR5021	Supercontig_2.1	100	16	0	0	0.036	32.2	*S. cerevisiae*	
ath-miR5021	Supercontig_2.1	100	16	0	0	0.036	32.2	*S. cerevisiae*	
ath-miR5021	Supercontig_2.1	100	16	0	0	0.036	32.2	*S. cerevisiae*	
ath-miR5021	Supercontig_2.1	100	16	0	0	0.036	32.2	*S. cerevisiae*	
ath-miR5021	Supercontig_2.1	100	16	0	0	0.036	32.2	*S. cerevisiae*	
ath-miR5021	Supercontig_2.1	100	16	0	0	0.036	32.2	*S. cerevisiae*	
ath-miR5021	Supercontig_2.1	100	16	0	0	0.036	32.2	*S. cerevisiae*	
ath-miR5021	Supercontig_2.1	100	16	0	0	0.036	32.2	*S. cerevisiae*	
ath-miR5021	Supercontig_2.1	100	16	0	0	0.036	32.2	*S. cerevisiae*	
ath-miR5021	Supercontig_2.1	100	16	0	0	0.036	32.2	*S. cerevisiae*	
ath-miR5021	Supercontig_2.1	100	16	0	0	0.036	32.2	*S. cerevisiae*	
ath-miR5021	Supercontig_2.1	100	16	0	0	0.036	32.2	*S. cerevisiae*	
ath-miR5021	Supercontig_2.1	100	16	0	0	0.036	32.2	*S. cerevisiae*	
ath-miR5021	Supercontig_2.1	100	16	0	0	0.036	32.2	*S. cerevisiae*	
ath-miR5021	Supercontig_2.1	100	16	0	0	0.036	32.2	*S. cerevisiae*	
ath-miR5021	Supercontig_2.1	100	16	0	0	0.036	32.2	*S. cerevisiae*	
ath-miR5021	Supercontig_2.1	100	16	0	0	0.036	32.2	*S. cerevisiae*	
ath-miR5021	Supercontig_2.1	100	16	0	0	0.036	32.2	*S. cerevisiae*	
ath-miR5021	Supercontig_2.1	100	16	0	0	0.036	32.2	*S. cerevisiae*	
ath-miR5021	Supercontig_2.1	100	16	0	0	0.036	32.2	*S. cerevisiae*	
ath-miR5021	Supercontig_2.1	100	16	0	0	0.036	32.2	*S. cerevisiae*	
ath-miR5021	Supercontig_2.1	100	16	0	0	0.036	32.2	*S. cerevisiae*	
ath-miR5021	Supercontig_2.1	100	16	0	0	0.036	32.2	*S. cerevisiae*	
ath-miR5021	Supercontig_2.1	100	16	0	0	0.036	32.2	*S. cerevisiae*	
ath-miR5021	Supercontig_2.1	100	16	0	0	0.036	32.2	*S. cerevisiae*	

Query id[a]	Subject id[b]	% Identity[c]	Alignment length[d]	Mismatches[e]	Gap opens[f]	Evalue [g]	Bit score[h]	Fungi vesicle[i]	Reference[j]
ath-miR5021	Supercontig_2.1	100	16	0	0	0.036	32.2	*S. cerevisiae*	
ath-miR5021	Supercontig_2.1	100	16	0	0	0.036	32.2	*S. cerevisiae*	
ath-miR5021	Supercontig_2.1	100	16	0	0	0.036	32.2	*S. cerevisiae*	
ath-miR5021	Supercontig_2.1	100	16	0	0	0.036	32.2	*S. cerevisiae*	
ath-miR5021	Supercontig_2.1	100	16	0	0	0.036	32.2	*S. cerevisiae*	
ath-miR5021	Supercontig_2.1	100	16	0	0	0.036	32.2	*S. cerevisiae*	
ath-miR5021	Supercontig_2.1	100	16	0	0	0.036	32.2	*S. cerevisiae*	
ath-miR5021	Supercontig_2.1	100	16	0	0	0.036	32.2	*S. cerevisiae*	
ath-miR5021	Supercontig_2.1	100	16	0	0	0.036	32.2	*S. cerevisiae*	
ath-miR5021	Supercontig_2.1	100	16	0	0	0.036	32.2	*S. cerevisiae*	
ath-miR5021	Supercontig_2.1	100	16	0	0	0.036	32.2	*S. cerevisiae*	
ath-miR5021	Supercontig_2.1	100	16	0	0	0.036	32.2	*S. cerevisiae*	
ath-miR5021	Supercontig_2.1	100	16	0	0	0.036	32.2	*S. cerevisiae*	
ath-miR5021	Supercontig_2.1	100	16	0	0	0.036	32.2	*S. cerevisiae*	
ath-miR5021	Supercontig_2.1	100	16	0	0	0.036	32.2	*S. cerevisiae*	
ath-miR5021	Supercontig_2.1	100	16	0	0	0.036	32.2	*S. cerevisiae*	
ath-miR5021	Supercontig_2.1	100	16	0	0	0.036	32.2	*S. cerevisiae*	
ath-miR5021	Supercontig_2.1	100	16	0	0	0.036	32.2	*S. cerevisiae*	
ath-miR5021	Supercontig_2.1	100	16	0	0	0.036	32.2	*S. cerevisiae*	
ath-miR5021	Supercontig_2.1	100	16	0	0	0.036	32.2	*S. cerevisiae*	
ath-miR5021	Supercontig_2.1	100	16	0	0	0.036	32.2	*S. cerevisiae*	
ath-miR5021	Supercontig_2.1	100	16	0	0	0.036	32.2	*S. cerevisiae*	
ath-miR5021	Supercontig_2.1	100	16	0	0	0.036	32.2	*S. cerevisiae*	
ath-miR5021	Supercontig_2.1	100	16	0	0	3.60E-02	32.2	*S. cerevisiae*	
ath-miR5021	Supercontig_2.1	100	16	0	0	0.036	32.2	*S. cerevisiae*	
ath-miR5021	Supercontig_2.1	100	16	0	0	0.036	32.2	*S. cerevisiae*	
ath-miR5021	Supercontig_2.1	100	16	0	0	0.036	32.2	*S. cerevisiae*	
ath-miR5021	Supercontig_2.1	100	16	0	0	0.036	32.2	*S. cerevisiae*	
ath-miR5021	Supercontig_2.1	100	16	0	0	0.036	32.2	*S. cerevisiae*	
ath-miR5021	Supercontig_2.1	100	16	0	0	0.036	32.2	*S. cerevisiae*	
ath-miR5021	Supercontig_2.1	100	16	0	0	0.036	32.2	*S. cerevisiae*	
ath-miR5021	Supercontig_2.1	100	16	0	0	0.036	32.2	*S. cerevisiae*	
ath-miR5021	Supercontig_2.1	100	16	0	0	0.036	32.2	*S. cerevisiae*	
ath-miR5021	Supercontig_2.1	100	16	0	0	0.036	32.2	*S. cerevisiae*	
ath-miR5021	Supercontig_2.1	100	16	0	0	0.036	32.2	*S. cerevisiae*	
ath-miR5021	Supercontig_2.1	100	16	0	0	0.036	32.2	*S. cerevisiae*	
ath-miR5021	Supercontig_2.1	100	16	0	0	0.036	32.2	*S. cerevisiae*	
ath-miR5021	Supercontig_2.1	100	16	0	0	0.036	32.2	*S. cerevisiae*	
ath-miR5021	Supercontig_2.1	100	16	0	0	0.036	32.2	*S. cerevisiae*	
ath-miR5021	Supercontig_2.1	100	16	0	0	0.036	32.2	*S. cerevisiae*	
ath-miR5021	Supercontig_2.1	100	16	0	0	0.036	32.2	*S. cerevisiae*	
ath-miR5021	Supercontig_2.1	100	16	0	0	0.036	32.2	*S. cerevisiae*	
ath-miR5021	Supercontig_2.1	100	16	0	0	0.036	32.2	*S. cerevisiae*	
ath-miR5021	Supercontig_2.1	100	16	0	0	0.036	32.2	*S. cerevisiae*	
ath-miR5021	Supercontig_2.1	100	16	0	0	0.036	32.2	*S. cerevisiae*	
ath-miR5021	Supercontig_2.1	100	16	0	0	0.036	32.2	*S. cerevisiae*	

Query id[a]	Subject id[b]	% Identity[c]	Alignment length[d]	Mismatches[e]	Gap opens[f]	Evalue [g]	Bit score[h]	Fungi vesicle[i]	Reference[j]
ath-miR5021	Supercontig_2.1	100	16	0	0	0.036	32.2	*S. cerevisiae*	
ath-miR5021	Supercontig_2.1	100	16	0	0	0.036	32.2	*S. cerevisiae*	
ath-miR5021	Supercontig_2.1	100	16	0	0	0.036	32.2	*S. cerevisiae*	
ath-miR5021	Supercontig_2.1	100	16	0	0	0.036	32.2	*S. cerevisiae*	
ath-miR5021	Supercontig_2.1	100	16	0	0	0.036	32.2	*S. cerevisiae*	
ath-miR5021	Supercontig_2.1	100	16	0	0	0.036	32.2	*S. cerevisiae*	
ath-miR5021	Supercontig_2.1	100	16	0	0	0.036	32.2	*S. cerevisiae*	
ath-miR5021	Supercontig_2.1	100	16	0	0	0.036	32.2	*S. cerevisiae*	
ath-miR5021	Supercontig_2.1	100	16	0	0	0.036	32.2	*S. cerevisiae*	
ath-miR5021	Supercontig_2.1	100	16	0	0	0.036	32.2	*S. cerevisiae*	
ath-miR5021	Supercontig_2.1	100	16	0	0	0.036	32.2	*S. cerevisiae*	
ath-miR5021	Supercontig_2.1	100	16	0	0	0.036	32.2	*S. cerevisiae*	
ath-miR5021	Supercontig_2.1	100	16	0	0	0.036	32.2	*S. cerevisiae*	
ath-miR5021	Supercontig_2.1	100	16	0	0	0.036	32.2	*S. cerevisiae*	
ath-miR5021	Supercontig_2.1	100	16	0	0	0.036	32.2	*S. cerevisiae*	
ath-miR5021	Supercontig_2.1	100	16	0	0	0.036	32.2	*S. cerevisiae*	
ath-miR5021	Supercontig_2.1	100	16	0	0	0.036	32.2	*S. cerevisiae*	
ath-miR5021	Supercontig_2.1	100	16	0	0	0.036	32.2	*S. cerevisiae*	
ath-miR5021	Supercontig_2.1	100	16	0	0	0.036	32.2	*S. cerevisiae*	
ath-miR5021	Supercontig_2.1	100	16	0	0	0.036	32.2	*S. cerevisiae*	
ath-miR5021	Supercontig_2.1	100	16	0	0	0.036	32.2	*S. cerevisiae*	
ath-miR5021	Supercontig_2.1	100	16	0	0	0.036	32.2	*S. cerevisiae*	
ath-miR5021	Supercontig_2.1	100	16	0	0	0.036	32.2	*S. cerevisiae*	
ath-miR5021	Supercontig_2.1	100	16	0	0	0.036	32.2	*S. cerevisiae*	
ath-miR5021	Supercontig_2.1	100	16	0	0	0.036	32.2	*S. cerevisiae*	
ath-miR5021	Supercontig_2.1	100	16	0	0	0.036	32.2	*S. cerevisiae*	
ath-miR5021	Supercontig_2.1	100	16	0	0	0.036	32.2	*S. cerevisiae*	
ath-miR5021	Supercontig_2.1	100	16	0	0	0.036	32.2	*S. cerevisiae*	
ath-miR5021	Supercontig_2.1	100	16	0	0	0.036	32.2	*S. cerevisiae*	
ath-miR5021	Supercontig_2.1	100	16	0	0	0.036	32.2	*S. cerevisiae*	
ath-miR5021	Supercontig_2.1	100	16	0	0	0.036	32.2	*S. cerevisiae*	
ath-miR5021	Supercontig_2.1	100	16	0	0	0.036	32.2	*S. cerevisiae*	
ath-miR5021	Supercontig_2.1	100	16	0	0	0.036	32.2	*S. cerevisiae*	
ath-miR5021	Supercontig_2.1	100	16	0	0	0.036	32.2	*S. cerevisiae*	
ath-miR5021	Supercontig_2.1	100	16	0	0	0.036	32.2	*S. cerevisiae*	
ath-miR5021	Supercontig_2.1	100	16	0	0	0.036	32.2	*S. cerevisiae*	
ath-miR5021	Supercontig_2.1	100	16	0	0	0.036	32.2	*S. cerevisiae*	
ath-miR5021	Supercontig_2.1	100	16	0	0	0.036	32.2	*S. cerevisiae*	
ath-miR5021	Supercontig_2.1	100	16	0	0	0.036	32.2	*S. cerevisiae*	
ath-miR5021	Supercontig_2.1	100	16	0	0	0.036	32.2	*S. cerevisiae*	
ath-miR5021	Supercontig_2.1	100	16	0	0	0.036	32.2	*S. cerevisiae*	
ath-miR5021	Supercontig_2.1	100	16	0	0	0.036	32.2	*S. cerevisiae*	
ath-miR5021	Supercontig_2.1	100	16	0	0	0.036	32.2	*S. cerevisiae*	
ath-miR5021	Supercontig_2.1	100	16	0	0	0.036	32.2	*S. cerevisiae*	
ath-miR5021	Supercontig_2.1	100	16	0	0	0.036	32.2	*S. cerevisiae*	
ath-miR5021	Supercontig_2.1	100	17	0	0	0.009	34.2	*S. cerevisiae*	

Query id[a]	Subject id[b]	% Identity[c]	Alignment length[d]	Mismatches[e]	Gap opens[f]	Evalue [g]	Bit score[h]	Fungi vesicle[i]	Reference[j]
ath-miR5021	Supercontig_2.1	100	17	0	0	0.009	34.2	*S. cerevisiae*	
ath-miR5021	Supercontig_2.1	100	17	0	0	0.009	34.2	*S. cerevisiae*	
ath-miR5021	Supercontig_2.1	100	17	0	0	0.009	34.2	*S. cerevisiae*	
ath-miR5021	Supercontig_2.1	100	17	0	0	0.009	34.2	*S. cerevisiae*	
ath-miR5021	Supercontig_2.1	100	17	0	0	0.009	34.2	*S. cerevisiae*	
ath-miR5021	Supercontig_2.1	100	17	0	0	0.009	34.2	*S. cerevisiae*	
ath-miR5021	Supercontig_2.1	100	17	0	0	0.009	34.2	*S. cerevisiae*	
ath-miR5021	Supercontig_2.1	100	17	0	0	0.009	34.2	*S. cerevisiae*	
ath-miR5021	Supercontig_2.1	100	17	0	0	0.009	34.2	*S. cerevisiae*	
ath-miR5021	Supercontig_2.1	100	17	0	0	0.009	34.2	*S. cerevisiae*	
ath-miR5021	Supercontig_2.1	100	18	0	0	0.002	36.2	*S. cerevisiae*	
ath-miR5021	Supercontig_2.1	100	18	0	0	0.002	36.2	*S. cerevisiae*	
ath-miR5021	Supercontig_2.1	100	18	0	0	0.002	36.2	*S. cerevisiae*	
ath-miR5021	Supercontig_2.1	100	18	0	0	0.002	36.2	*S. cerevisiae*	
ath-miR5021	Supercontig_2.1	100	18	0	0	0.002	36.2	*S. cerevisiae*	
ath-miR5021	Supercontig_2.1	100	18	0	0	0.002	36.2	*S. cerevisiae*	
ath-miR5021	Supercontig_2.1	100	18	0	0	0.002	36.2	*S. cerevisiae*	
ath-miR5021	Supercontig_2.1	100	18	0	0	0.002	36.2	*S. cerevisiae*	
ath-miR5021	Supercontig_2.1	100	19	0	0	6.00E-04	38.2	*S. cerevisiae*	
ath-miR5021	Supercontig_2.11	100	16	0	0	0.036	32.2	*S. cerevisiae*	
ath-miR5021	Supercontig_2.11	100	16	0	0	0.036	32.2	*S. cerevisiae*	
ath-miR5021	Supercontig_2.11	100	16	0	0	0.036	32.2	*S. cerevisiae*	
ath-miR5021	Supercontig_2.11	100	16	0	0	0.036	32.2	*S. cerevisiae*	
ath-miR5021	Supercontig_2.11	100	16	0	0	0.036	32.2	*S. cerevisiae*	
ath-miR5021	Supercontig_2.11	100	16	0	0	0.036	32.2	*S. cerevisiae*	
ath-miR5021	Supercontig_2.11	100	16	0	0	0.036	32.2	*S. cerevisiae*	
ath-miR5021	Supercontig_2.11	100	16	0	0	0.036	32.2	*S. cerevisiae*	
ath-miR5021	Supercontig_2.11	100	16	0	0	0.036	32.2	*S. cerevisiae*	
ath-miR5021	Supercontig_2.11	100	16	0	0	0.036	32.2	*S. cerevisiae*	
ath-miR5021	Supercontig_2.11	100	16	0	0	0.036	32.2	*S. cerevisiae*	
ath-miR5021	Supercontig_2.11	100	16	0	0	0.036	32.2	*S. cerevisiae*	
ath-miR5021	Supercontig_2.11	100	16	0	0	0.036	32.2	*S. cerevisiae*	
ath-miR5021	Supercontig_2.11	100	16	0	0	0.036	32.2	*S. cerevisiae*	
ath-miR5021	Supercontig_2.11	100	16	0	0	0.036	32.2	*S. cerevisiae*	
ath-miR5021	Supercontig_2.11	100	16	0	0	0.036	32.2	*S. cerevisiae*	
ath-miR5021	Supercontig_2.11	100	16	0	0	0.036	32.2	*S. cerevisiae*	
ath-miR5021	Supercontig_2.11	100	16	0	0	0.036	32.2	*S. cerevisiae*	
ath-miR5021	Supercontig_2.11	100	16	0	0	0.036	32.2	*S. cerevisiae*	
ath-miR5021	Supercontig_2.11	100	16	0	0	0.036	32.2	*S. cerevisiae*	
ath-miR5021	Supercontig_2.11	100	16	0	0	0.036	32.2	*S. cerevisiae*	
ath-miR5021	Supercontig_2.11	100	16	0	0	0.036	32.2	*S. cerevisiae*	
ath-miR5021	Supercontig_2.11	100	16	0	0	0.036	32.2	*S. cerevisiae*	
ath-miR5021	Supercontig_2.11	100	16	0	0	0.036	32.2	*S. cerevisiae*	
ath-miR5021	Supercontig_2.11	100	16	0	0	0.036	32.2	*S. cerevisiae*	
ath-miR5021	Supercontig_2.11	100	16	0	0	0.036	32.2	*S. cerevisiae*	
ath-miR5021	Supercontig_2.11	100	16	0	0	0.036	32.2	*S. cerevisiae*	

Query id[a]	Subject id[b]	% Identity[c]	Alignment length[d]	Mismatches[e]	Gap opens[f]	Evalue [g]	Bit score[h]	Fungi vesicle[i]	Reference[j]
ath-miR5021	Supercontig_2.11	100	16	0	0	0.036	32.2	*S. cerevisiae*	
ath-miR5021	Supercontig_2.11	100	16	0	0	0.036	32.2	*S. cerevisiae*	
ath-miR5021	Supercontig_2.11	100	16	0	0	0.036	32.2	*S. cerevisiae*	
ath-miR5021	Supercontig_2.11	100	16	0	0	0.036	32.2	*S. cerevisiae*	
ath-miR5021	Supercontig_2.11	100	16	0	0	0.036	32.2	*S. cerevisiae*	
ath-miR5021	Supercontig_2.11	100	16	0	0	0.036	32.2	*S. cerevisiae*	
ath-miR5021	Supercontig_2.11	100	16	0	0	0.036	32.2	*S. cerevisiae*	
ath-miR5021	Supercontig_2.11	100	16	0	0	0.036	32.2	*S. cerevisiae*	
ath-miR5021	Supercontig_2.11	100	16	0	0	0.036	32.2	*S. cerevisiae*	
ath-miR5021	Supercontig_2.11	100	16	0	0	0.036	32.2	*S. cerevisiae*	
ath-miR5021	Supercontig_2.11	100	16	0	0	0.036	32.2	*S. cerevisiae*	
ath-miR5021	Supercontig_2.11	100	16	0	0	0.036	32.2	*S. cerevisiae*	
ath-miR5021	Supercontig_2.11	100	16	0	0	0.036	32.2	*S. cerevisiae*	
ath-miR5021	Supercontig_2.11	100	16	0	0	0.036	32.2	*S. cerevisiae*	
ath-miR5021	Supercontig_2.11	100	16	0	0	0.036	32.2	*S. cerevisiae*	
ath-miR5021	Supercontig_2.11	100	16	0	0	0.036	32.2	*S. cerevisiae*	
ath-miR5021	Supercontig_2.11	100	16	0	0	0.036	32.2	*S. cerevisiae*	
ath-miR5021	Supercontig_2.11	100	16	0	0	0.036	32.2	*S. cerevisiae*	
ath-miR5021	Supercontig_2.11	100	16	0	0	0.036	32.2	*S. cerevisiae*	
ath-miR5021	Supercontig_2.11	100	16	0	0	0.036	32.2	*S. cerevisiae*	
ath-miR5021	Supercontig_2.11	100	16	0	0	0.036	32.2	*S. cerevisiae*	
ath-miR5021	Supercontig_2.11	100	16	0	0	0.036	32.2	*S. cerevisiae*	
ath-miR5021	Supercontig_2.11	100	16	0	0	0.036	32.2	*S. cerevisiae*	
ath-miR5021	Supercontig_2.11	100	16	0	0	0.036	32.2	*S. cerevisiae*	
ath-miR5021	Supercontig_2.11	100	16	0	0	0.036	32.2	*S. cerevisiae*	
ath-miR5021	Supercontig_2.11	100	16	0	0	0.036	32.2	*S. cerevisiae*	
ath-miR5021	Supercontig_2.11	100	16	0	0	0.036	32.2	*S. cerevisiae*	
ath-miR5021	Supercontig_2.11	100	16	0	0	0.036	32.2	*S. cerevisiae*	
ath-miR5021	Supercontig_2.11	100	16	0	0	0.036	32.2	*S. cerevisiae*	
ath-miR5021	Supercontig_2.11	100	16	0	0	0.036	32.2	*S. cerevisiae*	
ath-miR5021	Supercontig_2.11	100	16	0	0	0.036	32.2	*S. cerevisiae*	
ath-miR5021	Supercontig_2.11	100	16	0	0	0.036	32.2	*S. cerevisiae*	
ath-miR5021	Supercontig_2.11	100	16	0	0	0.036	32.2	*S. cerevisiae*	
ath-miR5021	Supercontig_2.11	100	16	0	0	0.036	32.2	*S. cerevisiae*	
ath-miR5021	Supercontig_2.11	100	16	0	0	0.036	32.2	*S. cerevisiae*	
ath-miR5021	Supercontig_2.11	100	16	0	0	0.036	32.2	*S. cerevisiae*	
ath-miR5021	Supercontig_2.11	100	16	0	0	0.036	32.2	*S. cerevisiae*	
ath-miR5021	Supercontig_2.11	100	16	0	0	0.036	32.2	*S. cerevisiae*	
ath-miR5021	Supercontig_2.11	100	16	0	0	0.036	32.2	*S. cerevisiae*	
ath-miR5021	Supercontig_2.11	100	16	0	0	0.036	32.2	*S. cerevisiae*	
ath-miR5021	Supercontig_2.11	100	16	0	0	0.036	32.2	*S. cerevisiae*	
ath-miR5021	Supercontig_2.11	100	16	0	0	0.036	32.2	*S. cerevisiae*	
ath-miR5021	Supercontig_2.11	100	16	0	0	0.036	32.2	*S. cerevisiae*	
ath-miR5021	Supercontig_2.11	100	16	0	0	0.036	32.2	*S. cerevisiae*	
ath-miR5021	Supercontig_2.11	100	16	0	0	0.036	32.2	*S. cerevisiae*	
ath-miR5021	Supercontig_2.11	100	16	0	0	0.036	32.2	*S. cerevisiae*	

Query id[a]	Subject id[b]	% Identity[c]	Alignment length[d]	Mismatches[e]	Gap opens[f]	Evalue [g]	Bit score[h]	Fungi vesicle[i]	Reference[j]
ath-miR5021	Supercontig_2.11	100	16	0	0	0.036	32.2	*S. cerevisiae*	
ath-miR5021	Supercontig_2.11	100	17	0	0	0.009	34.2	*S. cerevisiae*	
ath-miR5021	Supercontig_2.11	100	17	0	0	0.009	34.2	*S. cerevisiae*	
ath-miR5021	Supercontig_2.11	100	18	0	0	0.002	36.2	*S. cerevisiae*	
ath-miR5021	Supercontig_2.11	100	18	0	0	0.002	36.2	*S. cerevisiae*	
ath-miR5021	Supercontig_2.12	100	16	0	0	0.036	32.2	*S. cerevisiae*	
ath-miR5021	Supercontig_2.12	100	16	0	0	0.036	32.2	*S. cerevisiae*	
ath-miR5021	Supercontig_2.12	100	16	0	0	0.036	32.2	*S. cerevisiae*	
ath-miR5021	Supercontig_2.12	100	16	0	0	0.036	32.2	*S. cerevisiae*	
ath-miR5021	Supercontig_2.12	100	16	0	0	0.036	32.2	*S. cerevisiae*	
ath-miR5021	Supercontig_2.12	100	16	0	0	0.036	32.2	*S. cerevisiae*	
ath-miR5021	Supercontig_2.12	100	16	0	0	0.036	32.2	*S. cerevisiae*	
ath-miR5021	Supercontig_2.12	100	16	0	0	0.036	32.2	*S. cerevisiae*	
ath-miR5021	Supercontig_2.12	100	16	0	0	0.036	32.2	*S. cerevisiae*	
ath-miR5021	Supercontig_2.12	100	16	0	0	0.036	32.2	*S. cerevisiae*	
ath-miR5021	Supercontig_2.12	100	16	0	0	0.036	32.2	*S. cerevisiae*	
ath-miR5021	Supercontig_2.12	100	16	0	0	0.036	32.2	*S. cerevisiae*	
ath-miR5021	Supercontig_2.12	100	16	0	0	0.036	32.2	*S. cerevisiae*	
ath-miR5021	Supercontig_2.12	100	16	0	0	0.036	32.2	*S. cerevisiae*	
ath-miR5021	Supercontig_2.12	100	16	0	0	0.036	32.2	*S. cerevisiae*	
ath-miR5021	Supercontig_2.12	100	16	0	0	0.036	32.2	*S. cerevisiae*	
ath-miR5021	Supercontig_2.12	100	16	0	0	0.036	32.2	*S. cerevisiae*	
ath-miR5021	Supercontig_2.12	100	16	0	0	0.036	32.2	*S. cerevisiae*	
ath-miR5021	Supercontig_2.12	100	16	0	0	0.036	32.2	*S. cerevisiae*	
ath-miR5021	Supercontig_2.12	100	16	0	0	0.036	32.2	*S. cerevisiae*	
ath-miR5021	Supercontig_2.12	100	16	0	0	0.036	32.2	*S. cerevisiae*	
ath-miR5021	Supercontig_2.12	100	16	0	0	0.036	32.2	*S. cerevisiae*	
ath-miR5021	Supercontig_2.12	100	16	0	0	0.036	32.2	*S. cerevisiae*	
ath-miR5021	Supercontig_2.12	100	16	0	0	0.036	32.2	*S. cerevisiae*	
ath-miR5021	Supercontig_2.12	100	16	0	0	0.036	32.2	*S. cerevisiae*	
ath-miR5021	Supercontig_2.12	100	16	0	0	0.036	32.2	*S. cerevisiae*	
ath-miR5021	Supercontig_2.12	100	16	0	0	0.036	32.2	*S. cerevisiae*	
ath-miR5021	Supercontig_2.12	100	16	0	0	0.036	32.2	*S. cerevisiae*	
ath-miR5021	Supercontig_2.12	100	16	0	0	0.036	32.2	*S. cerevisiae*	
ath-miR5021	Supercontig_2.12	100	16	0	0	0.036	32.2	*S. cerevisiae*	
ath-miR5021	Supercontig_2.12	100	16	0	0	0.036	32.2	*S. cerevisiae*	
ath-miR5021	Supercontig_2.12	100	16	0	0	0.036	32.2	*S. cerevisiae*	
ath-miR5021	Supercontig_2.12	100	16	0	0	0.036	32.2	*S. cerevisiae*	
ath-miR5021	Supercontig_2.12	100	16	0	0	0.036	32.2	*S. cerevisiae*	
ath-miR5021	Supercontig_2.12	100	16	0	0	0.036	32.2	*S. cerevisiae*	
ath-miR5021	Supercontig_2.12	100	16	0	0	0.036	32.2	*S. cerevisiae*	
ath-miR5021	Supercontig_2.12	100	16	0	0	0.036	32.2	*S. cerevisiae*	
ath-miR5021	Supercontig_2.12	100	16	0	0	0.036	32.2	*S. cerevisiae*	
ath-miR5021	Supercontig_2.12	100	16	0	0	0.036	32.2	*S. cerevisiae*	
ath-miR5021	Supercontig_2.12	100	16	0	0	0.036	32.2	*S. cerevisiae*	
ath-miR5021	Supercontig_2.12	100	16	0	0	0.036	32.2	*S. cerevisiae*	

Query id[a]	Subject id[b]	% Identity[c]	Alignment length[d]	Mismatches[e]	Gap opens[f]	Evalue [g]	Bit score[h]	Fungi vesicle[i]	Reference[j]
ath-miR5021	Supercontig_2.12	100	16	0	0	0.036	32.2	*S. cerevisiae*	
ath-miR5021	Supercontig_2.12	100	16	0	0	0.036	32.2	*S. cerevisiae*	
ath-miR5021	Supercontig_2.12	100	16	0	0	0.036	32.2	*S. cerevisiae*	
ath-miR5021	Supercontig_2.12	100	16	0	0	0.036	32.2	*S. cerevisiae*	
ath-miR5021	Supercontig_2.12	100	16	0	0	0.036	32.2	*S. cerevisiae*	
ath-miR5021	Supercontig_2.12	100	16	0	0	0.036	32.2	*S. cerevisiae*	
ath-miR5021	Supercontig_2.12	100	16	0	0	0.036	32.2	*S. cerevisiae*	
ath-miR5021	Supercontig_2.12	100	16	0	0	0.036	32.2	*S. cerevisiae*	
ath-miR5021	Supercontig_2.12	100	16	0	0	0.036	32.2	*S. cerevisiae*	
ath-miR5021	Supercontig_2.12	100	17	0	0	0.009	34.2	*S. cerevisiae*	
ath-miR5021	Supercontig_2.12	100	17	0	0	0.009	34.2	*S. cerevisiae*	
ath-miR5021	Supercontig_2.12	100	17	0	0	0.009	34.2	*S. cerevisiae*	
ath-miR5021	Supercontig_2.12	100	17	0	0	0.009	34.2	*S. cerevisiae*	
ath-miR5021	Supercontig_2.13	100	16	0	0	0.036	32.2	*S. cerevisiae*	
ath-miR5021	Supercontig_2.13	100	16	0	0	0.036	32.2	*S. cerevisiae*	
ath-miR5021	Supercontig_2.13	100	16	0	0	0.036	32.2	*S. cerevisiae*	
ath-miR5021	Supercontig_2.13	100	16	0	0	0.036	32.2	*S. cerevisiae*	
ath-miR5021	Supercontig_2.13	100	16	0	0	0.036	32.2	*S. cerevisiae*	
ath-miR5021	Supercontig_2.13	100	16	0	0	0.036	32.2	*S. cerevisiae*	
ath-miR5021	Supercontig_2.13	100	16	0	0	0.036	32.2	*S. cerevisiae*	
ath-miR5021	Supercontig_2.13	100	16	0	0	0.036	32.2	*S. cerevisiae*	
ath-miR5021	Supercontig_2.13	100	16	0	0	0.036	32.2	*S. cerevisiae*	
ath-miR5021	Supercontig_2.13	100	16	0	0	0.036	32.2	*S. cerevisiae*	
ath-miR5021	Supercontig_2.13	100	16	0	0	0.036	32.2	*S. cerevisiae*	
ath-miR5021	Supercontig_2.13	100	16	0	0	0.036	32.2	*S. cerevisiae*	
ath-miR5021	Supercontig_2.13	100	16	0	0	0.036	32.2	*S. cerevisiae*	
ath-miR5021	Supercontig_2.13	100	16	0	0	0.036	32.2	*S. cerevisiae*	
ath-miR5021	Supercontig_2.13	100	16	0	0	0.036	32.2	*S. cerevisiae*	
ath-miR5021	Supercontig_2.13	100	16	0	0	0.036	32.2	*S. cerevisiae*	
ath-miR5021	Supercontig_2.13	100	16	0	0	0.036	32.2	*S. cerevisiae*	
ath-miR5021	Supercontig_2.13	100	16	0	0	0.036	32.2	*S. cerevisiae*	
ath-miR5021	Supercontig_2.13	100	16	0	0	0.036	32.2	*S. cerevisiae*	
ath-miR5021	Supercontig_2.13	100	16	0	0	0.036	32.2	*S. cerevisiae*	
ath-miR5021	Supercontig_2.13	100	16	0	0	0.036	32.2	*S. cerevisiae*	
ath-miR5021	Supercontig_2.13	100	16	0	0	0.036	32.2	*S. cerevisiae*	
ath-miR5021	Supercontig_2.13	100	16	0	0	0.036	32.2	*S. cerevisiae*	
ath-miR5021	Supercontig_2.13	100	16	0	0	0.036	32.2	*S. cerevisiae*	
ath-miR5021	Supercontig_2.13	100	16	0	0	0.036	32.2	*S. cerevisiae*	
ath-miR5021	Supercontig_2.13	100	16	0	0	0.036	32.2	*S. cerevisiae*	
ath-miR5021	Supercontig_2.13	100	16	0	0	0.036	32.2	*S. cerevisiae*	
ath-miR5021	Supercontig_2.13	100	16	0	0	0.036	32.2	*S. cerevisiae*	
ath-miR5021	Supercontig_2.13	100	16	0	0	0.036	32.2	*S. cerevisiae*	
ath-miR5021	Supercontig_2.13	100	16	0	0	0.036	32.2	*S. cerevisiae*	
ath-miR5021	Supercontig_2.13	100	16	0	0	0.036	32.2	*S. cerevisiae*	
ath-miR5021	Supercontig_2.13	100	16	0	0	0.036	32.2	*S. cerevisiae*	
ath-miR5021	Supercontig_2.13	100	16	0	0	0.036	32.2	*S. cerevisiae*	

Query id[a]	Subject id[b]	% Identity[c]	Alignment length[d]	Mismatches[e]	Gap opens[f]	Evalue [g]	Bit score[h]	Fungi vesicle[i]	Reference[j]
ath-miR5021	Supercontig_2.13	100	16	0	0	0.036	32.2	*S. cerevisiae*	
ath-miR5021	Supercontig_2.13	100	16	0	0	0.036	32.2	*S. cerevisiae*	
ath-miR5021	Supercontig_2.13	100	16	0	0	0.036	32.2	*S. cerevisiae*	
ath-miR5021	Supercontig_2.13	100	16	0	0	0.036	32.2	*S. cerevisiae*	
ath-miR5021	Supercontig_2.13	100	16	0	0	0.036	32.2	*S. cerevisiae*	
ath-miR5021	Supercontig_2.13	100	16	0	0	0.036	32.2	*S. cerevisiae*	
ath-miR5021	Supercontig_2.13	100	16	0	0	0.036	32.2	*S. cerevisiae*	
ath-miR5021	Supercontig_2.13	100	16	0	0	0.036	32.2	*S. cerevisiae*	
ath-miR5021	Supercontig_2.13	100	16	0	0	0.036	32.2	*S. cerevisiae*	
ath-miR5021	Supercontig_2.13	100	16	0	0	0.036	32.2	*S. cerevisiae*	
ath-miR5021	Supercontig_2.13	100	16	0	0	0.036	32.2	*S. cerevisiae*	
ath-miR5021	Supercontig_2.13	100	16	0	0	0.036	32.2	*S. cerevisiae*	
ath-miR5021	Supercontig_2.13	100	16	0	0	0.036	32.2	*S. cerevisiae*	
ath-miR5021	Supercontig_2.13	100	16	0	0	3.60E-02	32.2	*S. cerevisiae*	
ath-miR5021	Supercontig_2.13	100	16	0	0	0.036	32.2	*S. cerevisiae*	
ath-miR5021	Supercontig_2.13	100	16	0	0	0.036	32.2	*S. cerevisiae*	
ath-miR5021	Supercontig_2.13	100	16	0	0	0.036	32.2	*S. cerevisiae*	
ath-miR5021	Supercontig_2.13	100	16	0	0	0.036	32.2	*S. cerevisiae*	
ath-miR5021	Supercontig_2.13	100	16	0	0	0.036	32.2	*S. cerevisiae*	
ath-miR5021	Supercontig_2.13	100	16	0	0	0.036	32.2	*S. cerevisiae*	
ath-miR5021	Supercontig_2.13	100	16	0	0	0.036	32.2	*S. cerevisiae*	
ath-miR5021	Supercontig_2.13	100	16	0	0	0.036	32.2	*S. cerevisiae*	
ath-miR5021	Supercontig_2.13	100	16	0	0	0.036	32.2	*S. cerevisiae*	
ath-miR5021	Supercontig_2.13	100	16	0	0	0.036	32.2	*S. cerevisiae*	
ath-miR5021	Supercontig_2.13	100	16	0	0	0.036	32.2	*S. cerevisiae*	
ath-miR5021	Supercontig_2.13	100	16	0	0	0.036	32.2	*S. cerevisiae*	
ath-miR5021	Supercontig_2.13	100	16	0	0	0.036	32.2	*S. cerevisiae*	
ath-miR5021	Supercontig_2.13	100	16	0	0	0.036	32.2	*S. cerevisiae*	
ath-miR5021	Supercontig_2.13	100	16	0	0	0.036	32.2	*S. cerevisiae*	
ath-miR5021	Supercontig_2.13	100	16	0	0	0.036	32.2	*S. cerevisiae*	
ath-miR5021	Supercontig_2.13	100	16	0	0	0.036	32.2	*S. cerevisiae*	
ath-miR5021	Supercontig_2.13	100	16	0	0	0.036	32.2	*S. cerevisiae*	
ath-miR5021	Supercontig_2.13	100	16	0	0	0.036	32.2	*S. cerevisiae*	
ath-miR5021	Supercontig_2.13	100	16	0	0	0.036	32.2	*S. cerevisiae*	
ath-miR5021	Supercontig_2.13	100	16	0	0	0.036	32.2	*S. cerevisiae*	
ath-miR5021	Supercontig_2.13	100	16	0	0	0.036	32.2	*S. cerevisiae*	
ath-miR5021	Supercontig_2.13	100	16	0	0	0.036	32.2	*S. cerevisiae*	
ath-miR5021	Supercontig_2.13	100	16	0	0	0.036	32.2	*S. cerevisiae*	
ath-miR5021	Supercontig_2.13	100	16	0	0	0.036	32.2	*S. cerevisiae*	
ath-miR5021	Supercontig_2.13	100	16	0	0	0.036	32.2	*S. cerevisiae*	
ath-miR5021	Supercontig_2.13	100	16	0	0	0.036	32.2	*S. cerevisiae*	
ath-miR5021	Supercontig_2.13	100	16	0	0	0.036	32.2	*S. cerevisiae*	
ath-miR5021	Supercontig_2.13	100	16	0	0	0.036	32.2	*S. cerevisiae*	
ath-miR5021	Supercontig_2.13	100	16	0	0	0.036	32.2	*S. cerevisiae*	
ath-miR5021	Supercontig_2.13	100	16	0	0	0.036	32.2	*S. cerevisiae*	
ath-miR5021	Supercontig_2.13	100	16	0	0	0.036	32.2	*S. cerevisiae*	

Query id[a]	Subject id[b]	% Identity[c]	Alignment length[d]	Mismatches[e]	Gap opens[f]	Evalue [g]	Bit score[h]	Fungi vesicle[i]	Reference[j]
ath-miR5021	Supercontig_2.13	100	16	0	0	0.036	32.2	*S. cerevisiae*	
ath-miR5021	Supercontig_2.13	100	16	0	0	0.036	32.2	*S. cerevisiae*	
ath-miR5021	Supercontig_2.13	100	16	0	0	0.036	32.2	*S. cerevisiae*	
ath-miR5021	Supercontig_2.13	100	16	0	0	0.036	32.2	*S. cerevisiae*	
ath-miR5021	Supercontig_2.13	100	16	0	0	0.036	32.2	*S. cerevisiae*	
ath-miR5021	Supercontig_2.13	100	16	0	0	0.036	32.2	*S. cerevisiae*	
ath-miR5021	Supercontig_2.13	100	16	0	0	0.036	32.2	*S. cerevisiae*	
ath-miR5021	Supercontig_2.13	100	16	0	0	0.036	32.2	*S. cerevisiae*	
ath-miR5021	Supercontig_2.13	100	16	0	0	0.036	32.2	*S. cerevisiae*	
ath-miR5021	Supercontig_2.13	100	16	0	0	0.036	32.2	*S. cerevisiae*	
ath-miR5021	Supercontig_2.13	100	16	0	0	0.036	32.2	*S. cerevisiae*	
ath-miR5021	Supercontig_2.13	100	16	0	0	0.036	32.2	*S. cerevisiae*	
ath-miR5021	Supercontig_2.13	100	16	0	0	0.036	32.2	*S. cerevisiae*	
ath-miR5021	Supercontig_2.13	100	16	0	0	0.036	32.2	*S. cerevisiae*	
ath-miR5021	Supercontig_2.13	100	16	0	0	0.036	32.2	*S. cerevisiae*	
ath-miR5021	Supercontig_2.13	100	16	0	0	0.036	32.2	*S. cerevisiae*	
ath-miR5021	Supercontig_2.13	100	16	0	0	0.036	32.2	*S. cerevisiae*	
ath-miR5021	Supercontig_2.13	100	16	0	0	0.036	32.2	*S. cerevisiae*	
ath-miR5021	Supercontig_2.13	100	16	0	0	0.036	32.2	*S. cerevisiae*	
ath-miR5021	Supercontig_2.13	100	16	0	0	0.036	32.2	*S. cerevisiae*	
ath-miR5021	Supercontig_2.13	100	16	0	0	0.036	32.2	*S. cerevisiae*	
ath-miR5021	Supercontig_2.13	100	16	0	0	0.036	32.2	*S. cerevisiae*	
ath-miR5021	Supercontig_2.13	100	16	0	0	0.036	32.2	*S. cerevisiae*	
ath-miR5021	Supercontig_2.13	100	16	0	0	0.036	32.2	*S. cerevisiae*	
ath-miR5021	Supercontig_2.13	100	16	0	0	0.036	32.2	*S. cerevisiae*	
ath-miR5021	Supercontig_2.13	100	16	0	0	0.036	32.2	*S. cerevisiae*	
ath-miR5021	Supercontig_2.13	100	16	0	0	0.036	32.2	*S. cerevisiae*	
ath-miR5021	Supercontig_2.13	100	16	0	0	0.036	32.2	*S. cerevisiae*	
ath-miR5021	Supercontig_2.13	100	16	0	0	0.036	32.2	*S. cerevisiae*	
ath-miR5021	Supercontig_2.13	100	16	0	0	0.036	32.2	*S. cerevisiae*	
ath-miR5021	Supercontig_2.13	100	16	0	0	0.036	32.2	*S. cerevisiae*	
ath-miR5021	Supercontig_2.13	100	16	0	0	0.036	32.2	*S. cerevisiae*	
ath-miR5021	Supercontig_2.13	100	16	0	0	0.036	32.2	*S. cerevisiae*	
ath-miR5021	Supercontig_2.13	100	16	0	0	0.036	32.2	*S. cerevisiae*	
ath-miR5021	Supercontig_2.13	100	16	0	0	0.036	32.2	*S. cerevisiae*	
ath-miR5021	Supercontig_2.13	100	16	0	0	0.036	32.2	*S. cerevisiae*	
ath-miR5021	Supercontig_2.13	100	17	0	0	0.009	34.2	*S. cerevisiae*	
ath-miR5021	Supercontig_2.13	100	17	0	0	0.009	34.2	*S. cerevisiae*	
ath-miR5021	Supercontig_2.13	100	17	0	0	0.009	34.2	*S. cerevisiae*	
ath-miR5021	Supercontig_2.13	100	18	0	0	0.002	36.2	*S. cerevisiae*	
ath-miR5021	Supercontig_2.13	100	18	0	0	0.002	36.2	*S. cerevisiae*	
ath-miR5021	Supercontig_2.13	100	18	0	0	0.002	36.2	*S. cerevisiae*	
ath-miR5021	Supercontig_2.13	100	18	0	0	0.002	36.2	*S. cerevisiae*	
ath-miR5021	Supercontig_2.15	100	16	0	0	0.036	32.2	*S. cerevisiae*	
ath-miR5021	Supercontig_2.15	100	16	0	0	0.036	32.2	*S. cerevisiae*	
ath-miR5021	Supercontig_2.15	100	16	0	0	0.036	32.2	*S. cerevisiae*	

Query id[a]	Subject id[b]	% Identity[c]	Alignment length[d]	Mismatches[e]	Gap opens[f]	Evalue [g]	Bit score[h]	Fungi vesicle[i]	Reference[j]
ath-miR5021	Supercontig_2.15	100	16	0	0	0.036	32.2	*S. cerevisiae*	
ath-miR5021	Supercontig_2.15	100	16	0	0	0.036	32.2	*S. cerevisiae*	
ath-miR5021	Supercontig_2.15	100	16	0	0	0.036	32.2	*S. cerevisiae*	
ath-miR5021	Supercontig_2.15	100	16	0	0	0.036	32.2	*S. cerevisiae*	
ath-miR5021	Supercontig_2.15	100	16	0	0	0.036	32.2	*S. cerevisiae*	
ath-miR5021	Supercontig_2.16	100	16	0	0	0.036	32.2	*S. cerevisiae*	
ath-miR5021	Supercontig_2.16	100	16	0	0	0.036	32.2	*S. cerevisiae*	
ath-miR5021	Supercontig_2.16	100	16	0	0	0.036	32.2	*S. cerevisiae*	
ath-miR5021	Supercontig_2.16	100	16	0	0	0.036	32.2	*S. cerevisiae*	
ath-miR5021	Supercontig_2.16	100	16	0	0	0.036	32.2	*S. cerevisiae*	
ath-miR5021	Supercontig_2.16	100	16	0	0	0.036	32.2	*S. cerevisiae*	
ath-miR5021	Supercontig_2.16	100	16	0	0	0.036	32.2	*S. cerevisiae*	
ath-miR5021	Supercontig_2.16	100	16	0	0	0.036	32.2	*S. cerevisiae*	
ath-miR5021	Supercontig_2.16	100	16	0	0	0.036	32.2	*S. cerevisiae*	
ath-miR5021	Supercontig_2.16	100	16	0	0	0.036	32.2	*S. cerevisiae*	
ath-miR5021	Supercontig_2.16	100	16	0	0	0.036	32.2	*S. cerevisiae*	
ath-miR5021	Supercontig_2.16	100	16	0	0	0.036	32.2	*S. cerevisiae*	
ath-miR5021	Supercontig_2.16	100	18	0	0	0.002	36.2	*S. cerevisiae*	
ath-miR5021	Supercontig_2.17	100	16	0	0	0.036	32.2	*S. cerevisiae*	
ath-miR5021	Supercontig_2.17	100	16	0	0	0.036	32.2	*S. cerevisiae*	
ath-miR5021	Supercontig_2.17	100	16	0	0	0.036	32.2	*S. cerevisiae*	
ath-miR5021	Supercontig_2.17	100	16	0	0	0.036	32.2	*S. cerevisiae*	
ath-miR5021	Supercontig_2.17	100	16	0	0	0.036	32.2	*S. cerevisiae*	
ath-miR5021	Supercontig_2.17	100	16	0	0	0.036	32.2	*S. cerevisiae*	
ath-miR5021	Supercontig_2.17	100	16	0	0	0.036	32.2	*S. cerevisiae*	
ath-miR5021	Supercontig_2.17	100	16	0	0	0.036	32.2	*S. cerevisiae*	
ath-miR5021	Supercontig_2.17	100	16	0	0	0.036	32.2	*S. cerevisiae*	
ath-miR5021	Supercontig_2.17	100	16	0	0	0.036	32.2	*S. cerevisiae*	
ath-miR5021	Supercontig_2.17	100	16	0	0	0.036	32.2	*S. cerevisiae*	
ath-miR5021	Supercontig_2.17	100	16	0	0	0.036	32.2	*S. cerevisiae*	
ath-miR5021	Supercontig_2.17	100	16	0	0	0.036	32.2	*S. cerevisiae*	
ath-miR5021	Supercontig_2.17	100	16	0	0	0.036	32.2	*S. cerevisiae*	
ath-miR5021	Supercontig_2.17	100	16	0	0	0.036	32.2	*S. cerevisiae*	
ath-miR5021	Supercontig_2.17	100	16	0	0	0.036	32.2	*S. cerevisiae*	
ath-miR5021	Supercontig_2.17	100	16	0	0	0.036	32.2	*S. cerevisiae*	
ath-miR5021	Supercontig_2.17	100	16	0	0	0.036	32.2	*S. cerevisiae*	
ath-miR5021	Supercontig_2.17	100	16	0	0	0.036	32.2	*S. cerevisiae*	
ath-miR5021	Supercontig_2.17	100	16	0	0	0.036	32.2	*S. cerevisiae*	
ath-miR5021	Supercontig_2.17	100	16	0	0	0.036	32.2	*S. cerevisiae*	
ath-miR5021	Supercontig_2.17	100	16	0	0	0.036	32.2	*S. cerevisiae*	
ath-miR5021	Supercontig_2.17	100	16	0	0	0.036	32.2	*S. cerevisiae*	
ath-miR5021	Supercontig_2.17	100	16	0	0	0.036	32.2	*S. cerevisiae*	
ath-miR5021	Supercontig_2.17	100	16	0	0	0.036	32.2	*S. cerevisiae*	
ath-miR5021	Supercontig_2.17	100	17	0	0	0.009	34.2	*S. cerevisiae*	
ath-miR5021	Supercontig_2.17	100	18	0	0	0.002	36.2	*S. cerevisiae*	
ath-miR5021	Supercontig_2.18	100	16	0	0	0.036	32.2	*S. cerevisiae*	

Query id[a]	Subject id[b]	% Identity[c]	Alignment length[d]	Mismatches[e]	Gap opens[f]	Evalue [g]	Bit score[h]	Fungi vesicle[i]	Reference[j]
ath-miR5021	Supercontig_2.18	100	16	0	0	0.036	32.2	*S. cerevisiae*	
ath-miR5021	Supercontig_2.18	100	16	0	0	0.036	32.2	*S. cerevisiae*	
ath-miR5021	Supercontig_2.18	100	16	0	0	0.036	32.2	*S. cerevisiae*	
ath-miR5021	Supercontig_2.18	100	16	0	0	0.036	32.2	*S. cerevisiae*	
ath-miR5021	Supercontig_2.18	100	16	0	0	0.036	32.2	*S. cerevisiae*	
ath-miR5021	Supercontig_2.18	100	16	0	0	0.036	32.2	*S. cerevisiae*	
ath-miR5021	Supercontig_2.18	100	16	0	0	0.036	32.2	*S. cerevisiae*	
ath-miR5021	Supercontig_2.18	100	16	0	0	0.036	32.2	*S. cerevisiae*	
ath-miR5021	Supercontig_2.18	100	19	0	0	6.00E-04	38.2	*S. cerevisiae*	
ath-miR5021	Supercontig_2.2	100	16	0	0	0.036	32.2	*S. cerevisiae*	
ath-miR5021	Supercontig_2.2	100	16	0	0	0.036	32.2	*S. cerevisiae*	
ath-miR5021	Supercontig_2.2	100	16	0	0	0.036	32.2	*S. cerevisiae*	
ath-miR5021	Supercontig_2.2	100	16	0	0	0.036	32.2	*S. cerevisiae*	
ath-miR5021	Supercontig_2.2	100	16	0	0	0.036	32.2	*S. cerevisiae*	
ath-miR5021	Supercontig_2.2	100	16	0	0	0.036	32.2	*S. cerevisiae*	
ath-miR5021	Supercontig_2.2	100	16	0	0	0.036	32.2	*S. cerevisiae*	
ath-miR5021	Supercontig_2.2	100	16	0	0	0.036	32.2	*S. cerevisiae*	
ath-miR5021	Supercontig_2.2	100	16	0	0	0.036	32.2	*S. cerevisiae*	
ath-miR5021	Supercontig_2.2	100	16	0	0	0.036	32.2	*S. cerevisiae*	
ath-miR5021	Supercontig_2.2	100	16	0	0	0.036	32.2	*S. cerevisiae*	
ath-miR5021	Supercontig_2.2	100	16	0	0	0.036	32.2	*S. cerevisiae*	
ath-miR5021	Supercontig_2.2	100	16	0	0	0.036	32.2	*S. cerevisiae*	
ath-miR5021	Supercontig_2.2	100	16	0	0	0.036	32.2	*S. cerevisiae*	
ath-miR5021	Supercontig_2.2	100	16	0	0	0.036	32.2	*S. cerevisiae*	
ath-miR5021	Supercontig_2.2	100	16	0	0	0.036	32.2	*S. cerevisiae*	
ath-miR5021	Supercontig_2.2	100	16	0	0	0.036	32.2	*S. cerevisiae*	
ath-miR5021	Supercontig_2.2	100	16	0	0	0.036	32.2	*S. cerevisiae*	
ath-miR5021	Supercontig_2.2	100	16	0	0	0.036	32.2	*S. cerevisiae*	
ath-miR5021	Supercontig_2.2	100	16	0	0	0.036	32.2	*S. cerevisiae*	
ath-miR5021	Supercontig_2.2	100	16	0	0	0.036	32.2	*S. cerevisiae*	
ath-miR5021	Supercontig_2.2	100	16	0	0	0.036	32.2	*S. cerevisiae*	
ath-miR5021	Supercontig_2.2	100	16	0	0	0.036	32.2	*S. cerevisiae*	
ath-miR5021	Supercontig_2.2	100	16	0	0	0.036	32.2	*S. cerevisiae*	
ath-miR5021	Supercontig_2.2	100	16	0	0	0.036	32.2	*S. cerevisiae*	
ath-miR5021	Supercontig_2.2	100	16	0	0	0.036	32.2	*S. cerevisiae*	
ath-miR5021	Supercontig_2.2	100	16	0	0	0.036	32.2	*S. cerevisiae*	
ath-miR5021	Supercontig_2.2	100	16	0	0	0.036	32.2	*S. cerevisiae*	
ath-miR5021	Supercontig_2.2	100	16	0	0	0.036	32.2	*S. cerevisiae*	
ath-miR5021	Supercontig_2.2	100	16	0	0	0.036	32.2	*S. cerevisiae*	
ath-miR5021	Supercontig_2.2	100	16	0	0	0.036	32.2	*S. cerevisiae*	
ath-miR5021	Supercontig_2.2	100	16	0	0	0.036	32.2	*S. cerevisiae*	
ath-miR5021	Supercontig_2.2	100	16	0	0	0.036	32.2	*S. cerevisiae*	
ath-miR5021	Supercontig_2.2	100	16	0	0	0.036	32.2	*S. cerevisiae*	
ath-miR5021	Supercontig_2.2	100	16	0	0	0.036	32.2	*S. cerevisiae*	
ath-miR5021	Supercontig_2.2	100	16	0	0	0.036	32.2	*S. cerevisiae*	
ath-miR5021	Supercontig_2.2	100	16	0	0	0.036	32.2	*S. cerevisiae*	

Query id[a]	Subject id[b]	% Identity[c]	Alignment length[d]	Mismatches[e]	Gap opens[f]	Evalue [g]	Bit score[h]	Fungi vesicle[i]	Reference[j]
ath-miR5021	Supercontig_2.2	100	16	0	0	0.036	32.2	*S. cerevisiae*	
ath-miR5021	Supercontig_2.2	100	16	0	0	0.036	32.2	*S. cerevisiae*	
ath-miR5021	Supercontig_2.2	100	16	0	0	0.036	32.2	*S. cerevisiae*	
ath-miR5021	Supercontig_2.2	100	16	0	0	0.036	32.2	*S. cerevisiae*	
ath-miR5021	Supercontig_2.2	100	16	0	0	0.036	32.2	*S. cerevisiae*	
ath-miR5021	Supercontig_2.2	100	16	0	0	0.036	32.2	*S. cerevisiae*	
ath-miR5021	Supercontig_2.2	100	16	0	0	0.036	32.2	*S. cerevisiae*	
ath-miR5021	Supercontig_2.2	100	16	0	0	0.036	32.2	*S. cerevisiae*	
ath-miR5021	Supercontig_2.2	100	16	0	0	0.036	32.2	*S. cerevisiae*	
ath-miR5021	Supercontig_2.2	100	16	0	0	0.036	32.2	*S. cerevisiae*	
ath-miR5021	Supercontig_2.2	100	16	0	0	0.036	32.2	*S. cerevisiae*	
ath-miR5021	Supercontig_2.2	100	16	0	0	0.036	32.2	*S. cerevisiae*	
ath-miR5021	Supercontig_2.2	100	16	0	0	0.036	32.2	*S. cerevisiae*	
ath-miR5021	Supercontig_2.2	100	16	0	0	0.036	32.2	*S. cerevisiae*	
ath-miR5021	Supercontig_2.2	100	16	0	0	0.036	32.2	*S. cerevisiae*	
ath-miR5021	Supercontig_2.2	100	16	0	0	0.036	32.2	*S. cerevisiae*	
ath-miR5021	Supercontig_2.2	100	16	0	0	0.036	32.2	*S. cerevisiae*	
ath-miR5021	Supercontig_2.2	100	16	0	0	0.036	32.2	*S. cerevisiae*	
ath-miR5021	Supercontig_2.2	100	16	0	0	0.036	32.2	*S. cerevisiae*	
ath-miR5021	Supercontig_2.2	100	16	0	0	0.036	32.2	*S. cerevisiae*	
ath-miR5021	Supercontig_2.2	100	16	0	0	0.036	32.2	*S. cerevisiae*	
ath-miR5021	Supercontig_2.2	100	16	0	0	0.036	32.2	*S. cerevisiae*	
ath-miR5021	Supercontig_2.2	100	16	0	0	0.036	32.2	*S. cerevisiae*	
ath-miR5021	Supercontig_2.2	100	16	0	0	0.036	32.2	*S. cerevisiae*	
ath-miR5021	Supercontig_2.2	100	16	0	0	0.036	32.2	*S. cerevisiae*	
ath-miR5021	Supercontig_2.2	100	16	0	0	0.036	32.2	*S. cerevisiae*	
ath-miR5021	Supercontig_2.2	100	16	0	0	0.036	32.2	*S. cerevisiae*	
ath-miR5021	Supercontig_2.2	100	16	0	0	3.60E-02	32.2	*S. cerevisiae*	
ath-miR5021	Supercontig_2.2	100	16	0	0	0.036	32.2	*S. cerevisiae*	
ath-miR5021	Supercontig_2.2	100	16	0	0	0.036	32.2	*S. cerevisiae*	
ath-miR5021	Supercontig_2.2	100	16	0	0	0.036	32.2	*S. cerevisiae*	
ath-miR5021	Supercontig_2.2	100	16	0	0	0.036	32.2	*S. cerevisiae*	
ath-miR5021	Supercontig_2.2	100	16	0	0	0.036	32.2	*S. cerevisiae*	
ath-miR5021	Supercontig_2.2	100	16	0	0	0.036	32.2	*S. cerevisiae*	
ath-miR5021	Supercontig_2.2	100	16	0	0	0.036	32.2	*S. cerevisiae*	
ath-miR5021	Supercontig_2.2	100	16	0	0	0.036	32.2	*S. cerevisiae*	
ath-miR5021	Supercontig_2.2	100	16	0	0	0.036	32.2	*S. cerevisiae*	
ath-miR5021	Supercontig_2.2	100	16	0	0	0.036	32.2	*S. cerevisiae*	
ath-miR5021	Supercontig_2.2	100	16	0	0	0.036	32.2	*S. cerevisiae*	
ath-miR5021	Supercontig_2.2	100	16	0	0	0.036	32.2	*S. cerevisiae*	
ath-miR5021	Supercontig_2.2	100	16	0	0	0.036	32.2	*S. cerevisiae*	
ath-miR5021	Supercontig_2.2	100	16	0	0	0.036	32.2	*S. cerevisiae*	
ath-miR5021	Supercontig_2.2	100	16	0	0	0.036	32.2	*S. cerevisiae*	
ath-miR5021	Supercontig_2.2	100	16	0	0	0.036	32.2	*S. cerevisiae*	
ath-miR5021	Supercontig_2.2	100	16	0	0	0.036	32.2	*S. cerevisiae*	
ath-miR5021	Supercontig_2.2	100	16	0	0	0.036	32.2	*S. cerevisiae*	

Query id[a]	Subject id[b]	% Identity[c]	Alignment length[d]	Mismatches[e]	Gap opens[f]	Evalue [g]	Bit score[h]	Fungi vesicle[i]	Reference[j]
ath-miR5021	Supercontig_2.2	100	16	0	0	0.036	32.2	*S. cerevisiae*	
ath-miR5021	Supercontig_2.2	100	16	0	0	0.036	32.2	*S. cerevisiae*	
ath-miR5021	Supercontig_2.2	100	16	0	0	0.036	32.2	*S. cerevisiae*	
ath-miR5021	Supercontig_2.2	100	16	0	0	0.036	32.2	*S. cerevisiae*	
ath-miR5021	Supercontig_2.2	100	16	0	0	0.036	32.2	*S. cerevisiae*	
ath-miR5021	Supercontig_2.2	100	16	0	0	0.036	32.2	*S. cerevisiae*	
ath-miR5021	Supercontig_2.2	100	16	0	0	0.036	32.2	*S. cerevisiae*	
ath-miR5021	Supercontig_2.2	100	16	0	0	0.036	32.2	*S. cerevisiae*	
ath-miR5021	Supercontig_2.2	100	16	0	0	0.036	32.2	*S. cerevisiae*	
ath-miR5021	Supercontig_2.2	100	16	0	0	0.036	32.2	*S. cerevisiae*	
ath-miR5021	Supercontig_2.2	100	16	0	0	0.036	32.2	*S. cerevisiae*	
ath-miR5021	Supercontig_2.2	100	16	0	0	0.036	32.2	*S. cerevisiae*	
ath-miR5021	Supercontig_2.2	100	16	0	0	0.036	32.2	*S. cerevisiae*	
ath-miR5021	Supercontig_2.2	100	16	0	0	0.036	32.2	*S. cerevisiae*	
ath-miR5021	Supercontig_2.2	100	16	0	0	0.036	32.2	*S. cerevisiae*	
ath-miR5021	Supercontig_2.2	100	16	0	0	0.036	32.2	*S. cerevisiae*	
ath-miR5021	Supercontig_2.2	100	16	0	0	0.036	32.2	*S. cerevisiae*	
ath-miR5021	Supercontig_2.2	100	16	0	0	0.036	32.2	*S. cerevisiae*	
ath-miR5021	Supercontig_2.2	100	16	0	0	0.036	32.2	*S. cerevisiae*	
ath-miR5021	Supercontig_2.2	100	16	0	0	0.036	32.2	*S. cerevisiae*	
ath-miR5021	Supercontig_2.2	100	16	0	0	0.036	32.2	*S. cerevisiae*	
ath-miR5021	Supercontig_2.2	100	16	0	0	0.036	32.2	*S. cerevisiae*	
ath-miR5021	Supercontig_2.2	100	16	0	0	0.036	32.2	*S. cerevisiae*	
ath-miR5021	Supercontig_2.2	100	16	0	0	0.036	32.2	*S. cerevisiae*	
ath-miR5021	Supercontig_2.2	100	16	0	0	0.036	32.2	*S. cerevisiae*	
ath-miR5021	Supercontig_2.2	100	16	0	0	0.036	32.2	*S. cerevisiae*	
ath-miR5021	Supercontig_2.2	100	16	0	0	0.036	32.2	*S. cerevisiae*	
ath-miR5021	Supercontig_2.2	100	16	0	0	0.036	32.2	*S. cerevisiae*	
ath-miR5021	Supercontig_2.2	100	16	0	0	0.036	32.2	*S. cerevisiae*	
ath-miR5021	Supercontig_2.2	100	16	0	0	0.036	32.2	*S. cerevisiae*	
ath-miR5021	Supercontig_2.2	100	16	0	0	0.036	32.2	*S. cerevisiae*	
ath-miR5021	Supercontig_2.2	100	16	0	0	0.036	32.2	*S. cerevisiae*	
ath-miR5021	Supercontig_2.2	100	16	0	0	0.036	32.2	*S. cerevisiae*	
ath-miR5021	Supercontig_2.2	100	16	0	0	0.036	32.2	*S. cerevisiae*	
ath-miR5021	Supercontig_2.2	100	16	0	0	0.036	32.2	*S. cerevisiae*	
ath-miR5021	Supercontig_2.2	100	16	0	0	0.036	32.2	*S. cerevisiae*	
ath-miR5021	Supercontig_2.2	100	16	0	0	0.036	32.2	*S. cerevisiae*	
ath-miR5021	Supercontig_2.2	100	16	0	0	0.036	32.2	*S. cerevisiae*	
ath-miR5021	Supercontig_2.2	100	16	0	0	0.036	32.2	*S. cerevisiae*	
ath-miR5021	Supercontig_2.2	100	16	0	0	0.036	32.2	*S. cerevisiae*	
ath-miR5021	Supercontig_2.2	100	16	0	0	0.036	32.2	*S. cerevisiae*	
ath-miR5021	Supercontig_2.2	100	16	0	0	0.036	32.2	*S. cerevisiae*	
ath-miR5021	Supercontig_2.2	100	16	0	0	0.036	32.2	*S. cerevisiae*	
ath-miR5021	Supercontig_2.2	100	16	0	0	0.036	32.2	*S. cerevisiae*	
ath-miR5021	Supercontig_2.2	100	16	0	0	0.036	32.2	*S. cerevisiae*	
ath-miR5021	Supercontig_2.2	100	16	0	0	0.036	32.2	*S. cerevisiae*	

Query id[a]	Subject id[b]	% Identity[c]	Alignment length[d]	Mismatches[e]	Gap opens[f]	Evalue [g]	Bit score[h]	Fungi vesicle[i]	Reference[j]
ath-miR5021	Supercontig_2.2	100	16	0	0	0.036	32.2	*S. cerevisiae*	
ath-miR5021	Supercontig_2.2	100	16	0	0	0.036	32.2	*S. cerevisiae*	
ath-miR5021	Supercontig_2.2	100	16	0	0	0.036	32.2	*S. cerevisiae*	
ath-miR5021	Supercontig_2.2	100	16	0	0	0.036	32.2	*S. cerevisiae*	
ath-miR5021	Supercontig_2.2	100	16	0	0	0.036	32.2	*S. cerevisiae*	
ath-miR5021	Supercontig_2.2	100	16	0	0	0.036	32.2	*S. cerevisiae*	
ath-miR5021	Supercontig_2.2	100	16	0	0	0.036	32.2	*S. cerevisiae*	
ath-miR5021	Supercontig_2.2	100	16	0	0	0.036	32.2	*S. cerevisiae*	
ath-miR5021	Supercontig_2.2	100	16	0	0	0.036	32.2	*S. cerevisiae*	
ath-miR5021	Supercontig_2.2	100	16	0	0	0.036	32.2	*S. cerevisiae*	
ath-miR5021	Supercontig_2.2	100	16	0	0	0.036	32.2	*S. cerevisiae*	
ath-miR5021	Supercontig_2.2	100	16	0	0	0.036	32.2	*S. cerevisiae*	
ath-miR5021	Supercontig_2.2	100	16	0	0	0.036	32.2	*S. cerevisiae*	
ath-miR5021	Supercontig_2.2	100	16	0	0	0.036	32.2	*S. cerevisiae*	
ath-miR5021	Supercontig_2.2	100	16	0	0	0.036	32.2	*S. cerevisiae*	
ath-miR5021	Supercontig_2.2	100	16	0	0	0.036	32.2	*S. cerevisiae*	
ath-miR5021	Supercontig_2.2	100	16	0	0	0.036	32.2	*S. cerevisiae*	
ath-miR5021	Supercontig_2.2	100	16	0	0	0.036	32.2	*S. cerevisiae*	
ath-miR5021	Supercontig_2.2	100	16	0	0	0.036	32.2	*S. cerevisiae*	
ath-miR5021	Supercontig_2.2	100	16	0	0	0.036	32.2	*S. cerevisiae*	
ath-miR5021	Supercontig_2.2	100	16	0	0	0.036	32.2	*S. cerevisiae*	
ath-miR5021	Supercontig_2.2	100	16	0	0	0.036	32.2	*S. cerevisiae*	
ath-miR5021	Supercontig_2.2	100	16	0	0	0.036	32.2	*S. cerevisiae*	
ath-miR5021	Supercontig_2.2	100	16	0	0	0.036	32.2	*S. cerevisiae*	
ath-miR5021	Supercontig_2.2	100	16	0	0	0.036	32.2	*S. cerevisiae*	
ath-miR5021	Supercontig_2.2	100	16	0	0	0.036	32.2	*S. cerevisiae*	
ath-miR5021	Supercontig_2.2	100	16	0	0	0.036	32.2	*S. cerevisiae*	
ath-miR5021	Supercontig_2.2	100	16	0	0	0.036	32.2	*S. cerevisiae*	
ath-miR5021	Supercontig_2.2	100	16	0	0	0.036	32.2	*S. cerevisiae*	
ath-miR5021	Supercontig_2.2	100	16	0	0	0.036	32.2	*S. cerevisiae*	
ath-miR5021	Supercontig_2.2	100	16	0	0	0.036	32.2	*S. cerevisiae*	
ath-miR5021	Supercontig_2.2	100	16	0		874569	0.036	*S. cerevisiae*	
ath-miR5021	Supercontig_2.2	100	16	0	0	0.036	32.2	*S. cerevisiae*	
ath-miR5021	Supercontig_2.2	100	16	0	0	0.036	32.2	*S. cerevisiae*	
ath-miR5021	Supercontig_2.2	100	16	0	0	0.036	32.2	*S. cerevisiae*	
ath-miR5021	Supercontig_2.2	100	16	0	0	0.036	32.2	*S. cerevisiae*	
ath-miR5021	Supercontig_2.2	100	16	0	0	0.036	32.2	*S. cerevisiae*	
ath-miR5021	Supercontig_2.2	100	16	0	0	0.036	32.2	*S. cerevisiae*	
ath-miR5021	Supercontig_2.2	100	16	0	0	0.036	32.2	*S. cerevisiae*	
ath-miR5021	Supercontig_2.2	100	16	0	0	0.036	32.2	*S. cerevisiae*	
ath-miR5021	Supercontig_2.2	100	16	0	0	0.036	32.2	*S. cerevisiae*	
ath-miR5021	Supercontig_2.2	100	16	0	0	0.036	32.2	*S. cerevisiae*	
ath-miR5021	Supercontig_2.2	100	16	0	0	0.036	32.2	*S. cerevisiae*	
ath-miR5021	Supercontig_2.2	100	16	0	0	0.036	32.2	*S. cerevisiae*	
ath-miR5021	Supercontig_2.2	100	16	0	0	0.036	32.2	*S. cerevisiae*	
ath-miR5021	Supercontig_2.2	100	16	0	0	0.036	32.2	*S. cerevisiae*	

Query id[a]	Subject id[b]	% Identity[c]	Alignment length[d]	Mismatches[e]	Gap opens[f]	Evalue [g]	Bit score[h]	Fungi vesicle[i]	Reference[j]
ath-miR5021	Supercontig_2.2	100	16	0	0	0.036	32.2	*S. cerevisiae*	
ath-miR5021	Supercontig_2.2	100	16	0	0	0.036	32.2	*S. cerevisiae*	
ath-miR5021	Supercontig_2.2	100	16	0	0	0.036	32.2	*S. cerevisiae*	
ath-miR5021	Supercontig_2.2	100	16	0	0	0.036	32.2	*S. cerevisiae*	
ath-miR5021	Supercontig_2.2	100	16	0	0	0.036	32.2	*S. cerevisiae*	
ath-miR5021	Supercontig_2.2	100	17	0	0	0.009	34.2	*S. cerevisiae*	
ath-miR5021	Supercontig_2.2	100	17	0	0	0.009	34.2	*S. cerevisiae*	
ath-miR5021	Supercontig_2.2	100	17	0	0	0.009	34.2	*S. cerevisiae*	
ath-miR5021	Supercontig_2.2	100	17	0	0	0.009	34.2	*S. cerevisiae*	
ath-miR5021	Supercontig_2.2	100	17	0	0	0.009	34.2	*S. cerevisiae*	
ath-miR5021	Supercontig_2.2	100	17	0	0	0.009	34.2	*S. cerevisiae*	
ath-miR5021	Supercontig_2.2	100	17	0	0	0.009	34.2	*S. cerevisiae*	
ath-miR5021	Supercontig_2.2	100	17	0	0	0.009	34.2	*S. cerevisiae*	
ath-miR5021	Supercontig_2.2	100	18	0	0	0.002	36.2	*S. cerevisiae*	
ath-miR5021	Supercontig_2.2	100	18	0	0	0.002	36.2	*S. cerevisiae*	
ath-miR5021	Supercontig_2.2	100	18	0	0	0.002	36.2	*S. cerevisiae*	
ath-miR5021	Supercontig_2.2	100	18	0	0	0.002	36.2	*S. cerevisiae*	
ath-miR5021	Supercontig_2.2	100	18	0	0	0.002	36.2	*S. cerevisiae*	
ath-miR5021	Supercontig_2.2	100	18	0	0	0.002	36.2	*S. cerevisiae*	
ath-miR5021	Supercontig_2.2	100	18	0	0	0.002	36.2	*S. cerevisiae*	
ath-miR5021	Supercontig_2.2	100	18	0	0	0.002	36.2	*S. cerevisiae*	
ath-miR5021	Supercontig_2.2	100	18	0	0	0.002	36.2	*S. cerevisiae*	
ath-miR5021	Supercontig_2.2	100	19	0	0	6.00E-04	38.2	*S. cerevisiae*	
ath-miR5021	Supercontig_2.21	100	16	0	0	0.036	32.2	*S. cerevisiae*	
ath-miR5021	Supercontig_2.21	100	16	0	0	0.036	32.2	*S. cerevisiae*	
ath-miR5021	Supercontig_2.21	100	16	0	0	0.036	32.2	*S. cerevisiae*	
ath-miR5021	Supercontig_2.23	100	16	0	0	0.036	32.2	*S. cerevisiae*	
ath-miR5021	Supercontig_2.23	100	16	0	0	0.036	32.2	*S. cerevisiae*	
ath-miR5021	Supercontig_2.23	100	16	0	0	0.036	32.2	*S. cerevisiae*	
ath-miR5021	Supercontig_2.23	100	16	0	0	0.036	32.2	*S. cerevisiae*	
ath-miR5021	Supercontig_2.23	100	16	0	0	0.036	32.2	*S. cerevisiae*	
ath-miR5021	Supercontig_2.23	100	16	0	0	0.036	32.2	*S. cerevisiae*	
ath-miR5021	Supercontig_2.23	100	18	0	0	0.002	36.2	*S. cerevisiae*	
ath-miR5021	Supercontig_2.24	100	16	0	0	0.036	32.2	*S. cerevisiae*	
ath-miR5021	Supercontig_2.24	100	16	0	0	0.036	32.2	*S. cerevisiae*	
ath-miR5021	Supercontig_2.24	100	16	0	0	0.036	32.2	*S. cerevisiae*	
ath-miR5021	Supercontig_2.25	100	16	0	0	0.036	32.2	*S. cerevisiae*	
ath-miR5021	Supercontig_2.25	100	16	0	0	0.036	32.2	*S. cerevisiae*	
ath-miR5021	Supercontig_2.25	100	16	0	0	0.036	32.2	*S. cerevisiae*	
ath-miR5021	Supercontig_2.25	100	16	0	0	0.036	32.2	*S. cerevisiae*	
ath-miR5021	Supercontig_2.25	100	17	0	0	0.009	34.2	*S. cerevisiae*	
ath-miR5021	Supercontig_2.3	100	16	0	0	0.036	32.2	*S. cerevisiae*	
ath-miR5021	Supercontig_2.3	100	16	0	0	0.036	32.2	*S. cerevisiae*	
ath-miR5021	Supercontig_2.3	100	16	0	0	0.036	32.2	*S. cerevisiae*	
ath-miR5021	Supercontig_2.3	100	16	0	0	0.036	32.2	*S. cerevisiae*	
ath-miR5021	Supercontig_2.3	100	16	0	0	0.036	32.2	*S. cerevisiae*	

Query id[a]	Subject id[b]	% Identity[c]	Alignment length[d]	Mismatches[e]	Gap opens[f]	Evalue [g]	Bit score[h]	Fungi vesicle[i]	Reference[j]
ath-miR5021	Supercontig_2.3	100	16	0	0	0.036	32.2	*S. cerevisiae*	
ath-miR5021	Supercontig_2.3	100	16	0	0	0.036	32.2	*S. cerevisiae*	
ath-miR5021	Supercontig_2.3	100	16	0	0	0.036	32.2	*S. cerevisiae*	
ath-miR5021	Supercontig_2.3	100	16	0	0	0.036	32.2	*S. cerevisiae*	
ath-miR5021	Supercontig_2.3	100	16	0	0	0.036	32.2	*S. cerevisiae*	
ath-miR5021	Supercontig_2.3	100	16	0	0	0.036	32.2	*S. cerevisiae*	
ath-miR5021	Supercontig_2.3	100	16	0	0	0.036	32.2	*S. cerevisiae*	
ath-miR5021	Supercontig_2.3	100	16	0	0	0.036	32.2	*S. cerevisiae*	
ath-miR5021	Supercontig_2.3	100	16	0	0	0.036	32.2	*S. cerevisiae*	
ath-miR5021	Supercontig_2.3	100	16	0	0	0.036	32.2	*S. cerevisiae*	
ath-miR5021	Supercontig_2.3	100	16	0	0	0.036	32.2	*S. cerevisiae*	
ath-miR5021	Supercontig_2.3	100	16	0	0	0.036	32.2	*S. cerevisiae*	
ath-miR5021	Supercontig_2.3	100	16	0	0	0.036	32.2	*S. cerevisiae*	
ath-miR5021	Supercontig_2.3	100	16	0	0	0.036	32.2	*S. cerevisiae*	
ath-miR5021	Supercontig_2.3	100	16	0	0	0.036	32.2	*S. cerevisiae*	
ath-miR5021	Supercontig_2.3	100	16	0	0	0.036	32.2	*S. cerevisiae*	
ath-miR5021	Supercontig_2.3	100	16	0	0	0.036	32.2	*S. cerevisiae*	
ath-miR5021	Supercontig_2.3	100	16	0	0	0.036	32.2	*S. cerevisiae*	
ath-miR5021	Supercontig_2.3	100	16	0	0	0.036	32.2	*S. cerevisiae*	
ath-miR5021	Supercontig_2.3	100	16	0	0	0.036	32.2	*S. cerevisiae*	
ath-miR5021	Supercontig_2.3	100	16	0	0	0.036	32.2	*S. cerevisiae*	
ath-miR5021	Supercontig_2.3	100	16	0	0	0.036	32.2	*S. cerevisiae*	
ath-miR5021	Supercontig_2.3	100	16	0	0	0.036	32.2	*S. cerevisiae*	
ath-miR5021	Supercontig_2.3	100	16	0	0	0.036	32.2	*S. cerevisiae*	
ath-miR5021	Supercontig_2.3	100	16	0	0	0.036	32.2	*S. cerevisiae*	
ath-miR5021	Supercontig_2.3	100	16	0	0	0.036	32.2	*S. cerevisiae*	
ath-miR5021	Supercontig_2.3	100	16	0	0	0.036	32.2	*S. cerevisiae*	
ath-miR5021	Supercontig_2.3	100	16	0	0	0.036	32.2	*S. cerevisiae*	
ath-miR5021	Supercontig_2.3	100	16	0	0	0.036	32.2	*S. cerevisiae*	
ath-miR5021	Supercontig_2.3	100	16	0	0	0.036	32.2	*S. cerevisiae*	
ath-miR5021	Supercontig_2.3	100	16	0	0	0.036	32.2	*S. cerevisiae*	
ath-miR5021	Supercontig_2.3	100	16	0	0	0.036	32.2	*S. cerevisiae*	
ath-miR5021	Supercontig_2.3	100	16	0	0	0.036	32.2	*S. cerevisiae*	
ath-miR5021	Supercontig_2.3	100	16	0	0	0.036	32.2	*S. cerevisiae*	
ath-miR5021	Supercontig_2.3	100	16	0	0	0.036	32.2	*S. cerevisiae*	
ath-miR5021	Supercontig_2.3	100	16	0	0	0.036	32.2	*S. cerevisiae*	
ath-miR5021	Supercontig_2.3	100	16	0	0	0.036	32.2	*S. cerevisiae*	
ath-miR5021	Supercontig_2.3	100	16	0	0	0.036	32.2	*S. cerevisiae*	
ath-miR5021	Supercontig_2.3	100	16	0	0	0.036	32.2	*S. cerevisiae*	
ath-miR5021	Supercontig_2.3	100	16	0	0	0.036	32.2	*S. cerevisiae*	
ath-miR5021	Supercontig_2.3	100	16	0	0	0.036	32.2	*S. cerevisiae*	
ath-miR5021	Supercontig_2.3	100	16	0	0	0.036	32.2	*S. cerevisiae*	
ath-miR5021	Supercontig_2.3	100	16	0	0	0.036	32.2	*S. cerevisiae*	
ath-miR5021	Supercontig_2.3	100	16	0	0	0.036	32.2	*S. cerevisiae*	
ath-miR5021	Supercontig_2.3	100	16	0	0	0.036	32.2	*S. cerevisiae*	
ath-miR5021	Supercontig_2.3	100	16	0	0	0.036	32.2	*S. cerevisiae*	

Query id[a]	Subject id[b]	% Identity[c]	Alignment length[d]	Mismatches[e]	Gap opens[f]	Evalue [g]	Bit score[h]	Fungi vesicle[i]	Reference[j]
ath-miR5021	Supercontig_2.3	100	16	0	0	0.036	32.2	*S. cerevisiae*	
ath-miR5021	Supercontig_2.3	100	16	0	0	0.036	32.2	*S. cerevisiae*	
ath-miR5021	Supercontig_2.3	100	16	0	0	0.036	32.2	*S. cerevisiae*	
ath-miR5021	Supercontig_2.3	100	16	0	0	0.036	32.2	*S. cerevisiae*	
ath-miR5021	Supercontig_2.3	100	16	0	0	0.036	32.2	*S. cerevisiae*	
ath-miR5021	Supercontig_2.3	100	16	0	0	0.036	32.2	*S. cerevisiae*	
ath-miR5021	Supercontig_2.3	100	16	0	0	0.036	32.2	*S. cerevisiae*	
ath-miR5021	Supercontig_2.3	100	16	0	0	0.036	32.2	*S. cerevisiae*	
ath-miR5021	Supercontig_2.3	100	16	0	0	0.036	32.2	*S. cerevisiae*	
ath-miR5021	Supercontig_2.3	100	16	0	0	0.036	32.2	*S. cerevisiae*	
ath-miR5021	Supercontig_2.3	100	16	0	0	0.036	32.2	*S. cerevisiae*	
ath-miR5021	Supercontig_2.3	100	16	0	0	0.036	32.2	*S. cerevisiae*	
ath-miR5021	Supercontig_2.3	100	16	0	0	0.036	32.2	*S. cerevisiae*	
ath-miR5021	Supercontig_2.3	100	16	0	0	0.036	32.2	*S. cerevisiae*	
ath-miR5021	Supercontig_2.3	100	16	0	0	0.036	32.2	*S. cerevisiae*	
ath-miR5021	Supercontig_2.3	100	16	0	0	0.036	32.2	*S. cerevisiae*	
ath-miR5021	Supercontig_2.3	100	16	0	0	0.036	32.2	*S. cerevisiae*	
ath-miR5021	Supercontig_2.3	100	16	0	0	0.036	32.2	*S. cerevisiae*	
ath-miR5021	Supercontig_2.3	100	16	0	0	0.036	32.2	*S. cerevisiae*	
ath-miR5021	Supercontig_2.3	100	16	0	0	0.036	32.2	*S. cerevisiae*	
ath-miR5021	Supercontig_2.3	100	16	0	0	0.036	32.2	*S. cerevisiae*	
ath-miR5021	Supercontig_2.3	100	16	0	0	0.036	32.2	*S. cerevisiae*	
ath-miR5021	Supercontig_2.3	100	16	0	0	0.036	32.2	*S. cerevisiae*	
ath-miR5021	Supercontig_2.3	100	16	0	0	0.036	32.2	*S. cerevisiae*	
ath-miR5021	Supercontig_2.3	100	16	0	0	0.036	32.2	*S. cerevisiae*	
ath-miR5021	Supercontig_2.3	100	16	0	0	0.036	32.2	*S. cerevisiae*	
ath-miR5021	Supercontig_2.3	100	16	0	0	0.036	32.2	*S. cerevisiae*	
ath-miR5021	Supercontig_2.3	100	16	0	0	0.036	32.2	*S. cerevisiae*	
ath-miR5021	Supercontig_2.3	100	16	0	0	0.036	32.2	*S. cerevisiae*	
ath-miR5021	Supercontig_2.3	100	16	0	0	0.036	32.2	*S. cerevisiae*	
ath-miR5021	Supercontig_2.3	100	16	0	0	0.036	32.2	*S. cerevisiae*	
ath-miR5021	Supercontig_2.3	100	16	0	0	0.036	32.2	*S. cerevisiae*	
ath-miR5021	Supercontig_2.3	100	16	0	0	0.036	32.2	*S. cerevisiae*	
ath-miR5021	Supercontig_2.3	100	16	0	0	0.036	32.2	*S. cerevisiae*	
ath-miR5021	Supercontig_2.3	100	16	0	0	0.036	32.2	*S. cerevisiae*	
ath-miR5021	Supercontig_2.3	100	16	0	0	0.036	32.2	*S. cerevisiae*	
ath-miR5021	Supercontig_2.3	100	16	0	0	0.036	32.2	*S. cerevisiae*	
ath-miR5021	Supercontig_2.3	100	16	0	0	0.036	32.2	*S. cerevisiae*	
ath-miR5021	Supercontig_2.3	100	16	0	0	0.036	32.2	*S. cerevisiae*	
ath-miR5021	Supercontig_2.3	100	16	0	0	0.036	32.2	*S. cerevisiae*	
ath-miR5021	Supercontig_2.3	100	16	0	0	0.036	32.2	*S. cerevisiae*	
ath-miR5021	Supercontig_2.3	100	16	0	0	0.036	32.2	*S. cerevisiae*	
ath-miR5021	Supercontig_2.3	100	16	0	0	0.036	32.2	*S. cerevisiae*	
ath-miR5021	Supercontig_2.3	100	16	0	0	0.036	32.2	*S. cerevisiae*	
ath-miR5021	Supercontig_2.3	100	16	0	0	0.036	32.2	*S. cerevisiae*	
ath-miR5021	Supercontig_2.3	100	16	0	0	0.036	32.2	*S. cerevisiae*	

Query id[a]	Subject id[b]	% Identity[c]	Alignment length[d]	Mismatches[e]	Gap opens[f]	Evalue [g]	Bit score[h]	Fungi vesicle[i]	Reference[j]
ath-miR5021	Supercontig_2.3	100	16	0	0	0.036	32.2	*S. cerevisiae*	
ath-miR5021	Supercontig_2.3	100	16	0	0	0.036	32.2	*S. cerevisiae*	
ath-miR5021	Supercontig_2.3	100	16	0	0	0.036	32.2	*S. cerevisiae*	
ath-miR5021	Supercontig_2.3	100	16	0	0	0.036	32.2	*S. cerevisiae*	
ath-miR5021	Supercontig_2.3	100	16	0	0	0.036	32.2	*S. cerevisiae*	
ath-miR5021	Supercontig_2.3	100	16	0	0	0.036	32.2	*S. cerevisiae*	
ath-miR5021	Supercontig_2.3	100	16	0	0	0.036	32.2	*S. cerevisiae*	
ath-miR5021	Supercontig_2.3	100	16	0	0	0.036	32.2	*S. cerevisiae*	
ath-miR5021	Supercontig_2.3	100	16	0	0	0.036	32.2	*S. cerevisiae*	
ath-miR5021	Supercontig_2.3	100	16	0	0	0.036	32.2	*S. cerevisiae*	
ath-miR5021	Supercontig_2.3	100	16	0	0	0.036	32.2	*S. cerevisiae*	
ath-miR5021	Supercontig_2.3	100	16	0	0	0.036	32.2	*S. cerevisiae*	
ath-miR5021	Supercontig_2.3	100	16	0	0	0.036	32.2	*S. cerevisiae*	
ath-miR5021	Supercontig_2.3	100	16	0	0	0.036	32.2	*S. cerevisiae*	
ath-miR5021	Supercontig_2.3	100	16	0	0	0.036	32.2	*S. cerevisiae*	
ath-miR5021	Supercontig_2.3	100	16	0	0	0.036	32.2	*S. cerevisiae*	
ath-miR5021	Supercontig_2.3	100	16	0	0	0.036	32.2	*S. cerevisiae*	
ath-miR5021	Supercontig_2.3	100	16	0	0	0.036	32.2	*S. cerevisiae*	
ath-miR5021	Supercontig_2.3	100	16	0	0	0.036	32.2	*S. cerevisiae*	
ath-miR5021	Supercontig_2.3	100	16	0	0	0.036	32.2	*S. cerevisiae*	
ath-miR5021	Supercontig_2.3	100	16	0	0	0.036	32.2	*S. cerevisiae*	
ath-miR5021	Supercontig_2.3	100	16	0	0	0.036	32.2	*S. cerevisiae*	
ath-miR5021	Supercontig_2.3	100	16	0	0	0.036	32.2	*S. cerevisiae*	
ath-miR5021	Supercontig_2.3	100	16	0	0	0.036	32.2	*S. cerevisiae*	
ath-miR5021	Supercontig_2.3	100	16	0	0	0.036	32.2	*S. cerevisiae*	
ath-miR5021	Supercontig_2.3	100	16	0	0	0.036	32.2	*S. cerevisiae*	
ath-miR5021	Supercontig_2.3	100	16	0	0	0.036	32.2	*S. cerevisiae*	
ath-miR5021	Supercontig_2.3	100	16	0	0	0.036	32.2	*S. cerevisiae*	
ath-miR5021	Supercontig_2.3	100	16	0	0	0.036	32.2	*S. cerevisiae*	
ath-miR5021	Supercontig_2.3	100	16	0	0	0.036	32.2	*S. cerevisiae*	
ath-miR5021	Supercontig_2.3	100	16	0	0	0.036	32.2	*S. cerevisiae*	
ath-miR5021	Supercontig_2.3	100	16	0	0	0.036	32.2	*S. cerevisiae*	
ath-miR5021	Supercontig_2.3	100	16	0	0	0.036	32.2	*S. cerevisiae*	
ath-miR5021	Supercontig_2.3	100	16	0	0	0.036	32.2	*S. cerevisiae*	
ath-miR5021	Supercontig_2.3	100	16	0	0	0.036	32.2	*S. cerevisiae*	
ath-miR5021	Supercontig_2.3	100	16	0	0	0.036	32.2	*S. cerevisiae*	
ath-miR5021	Supercontig_2.3	100	16	0	0	0.036	32.2	*S. cerevisiae*	
ath-miR5021	Supercontig_2.3	100	16	0	0	0.036	32.2	*S. cerevisiae*	
ath-miR5021	Supercontig_2.3	100	16	0	0	0.036	32.2	*S. cerevisiae*	
ath-miR5021	Supercontig_2.3	100	16	0	0	0.036	32.2	*S. cerevisiae*	
ath-miR5021	Supercontig_2.3	100	16	0	0	0.036	32.2	*S. cerevisiae*	
ath-miR5021	Supercontig_2.3	100	16	0	0	0.036	32.2	*S. cerevisiae*	
ath-miR5021	Supercontig_2.3	100	16	0	0	0.036	32.2	*S. cerevisiae*	
ath-miR5021	Supercontig_2.3	100	16	0	0	0.036	32.2	*S. cerevisiae*	
ath-miR5021	Supercontig_2.3	100	16	0	0	0.036	32.2	*S. cerevisiae*	
ath-miR5021	Supercontig_2.3	100	16	0	0	0.036	32.2	*S. cerevisiae*	

Query id[a]	Subject id[b]	% Identity[c]	Alignment length[d]	Mismatches[e]	Gap opens[f]	Evalue [g]	Bit score[h]	Fungi vesicle[i]	Reference[j]
ath-miR5021	Supercontig_2.3	100	16	0	0	0.036	32.2	*S. cerevisiae*	
ath-miR5021	Supercontig_2.3	100	16	0	0	0.036	32.2	*S. cerevisiae*	
ath-miR5021	Supercontig_2.3	100	16	0	0	0.036	32.2	*S. cerevisiae*	
ath-miR5021	Supercontig_2.3	100	16	0	0	0.036	32.2	*S. cerevisiae*	
ath-miR5021	Supercontig_2.3	100	16	0	0	0.036	32.2	*S. cerevisiae*	
ath-miR5021	Supercontig_2.3	100	16	0	0	0.036	32.2	*S. cerevisiae*	
ath-miR5021	Supercontig_2.3	100	16	0	0	0.036	32.2	*S. cerevisiae*	
ath-miR5021	Supercontig_2.3	100	16	0	0	0.036	32.2	*S. cerevisiae*	
ath-miR5021	Supercontig_2.3	100	16	0	0	0.036	32.2	*S. cerevisiae*	
ath-miR5021	Supercontig_2.3	100	16	0	0	0.036	32.2	*S. cerevisiae*	
ath-miR5021	Supercontig_2.3	100	16	0	0	0.036	32.2	*S. cerevisiae*	
ath-miR5021	Supercontig_2.3	100	16	0	0	0.036	32.2	*S. cerevisiae*	
ath-miR5021	Supercontig_2.3	100	16	0	0	0.036	32.2	*S. cerevisiae*	
ath-miR5021	Supercontig_2.3	100	16	0	0	0.036	32.2	*S. cerevisiae*	
ath-miR5021	Supercontig_2.3	100	16	0	0	0.036	32.2	*S. cerevisiae*	
ath-miR5021	Supercontig_2.3	100	16	0	0	0.036	32.2	*S. cerevisiae*	
ath-miR5021	Supercontig_2.3	100	16	0	0	0.036	32.2	*S. cerevisiae*	
ath-miR5021	Supercontig_2.3	100	16	0	0	0.036	32.2	*S. cerevisiae*	
ath-miR5021	Supercontig_2.3	100	16	0	0	0.036	32.2	*S. cerevisiae*	
ath-miR5021	Supercontig_2.3	100	16	0	0	0.036	32.2	*S. cerevisiae*	
ath-miR5021	Supercontig_2.3	100	16	0	0	0.036	32.2	*S. cerevisiae*	
ath-miR5021	Supercontig_2.3	100	16	0	0	0.036	32.2	*S. cerevisiae*	
ath-miR5021	Supercontig_2.3	100	16	0	0	0.036	32.2	*S. cerevisiae*	
ath-miR5021	Supercontig_2.3	100	16	0	0	0.036	32.2	*S. cerevisiae*	
ath-miR5021	Supercontig_2.3	100	16	0	0	0.036	32.2	*S. cerevisiae*	
ath-miR5021	Supercontig_2.3	100	16	0	0	0.036	32.2	*S. cerevisiae*	
ath-miR5021	Supercontig_2.3	100	16	0	0	0.036	32.2	*S. cerevisiae*	
ath-miR5021	Supercontig_2.3	100	16	0	0	0.036	32.2	*S. cerevisiae*	
ath-miR5021	Supercontig_2.3	100	16	0	0	0.036	32.2	*S. cerevisiae*	
ath-miR5021	Supercontig_2.3	100	16	0	0	0.036	32.2	*S. cerevisiae*	
ath-miR5021	Supercontig_2.3	100	16	0	0	0.036	32.2	*S. cerevisiae*	
ath-miR5021	Supercontig_2.3	100	16	0	0	0.036	32.2	*S. cerevisiae*	
ath-miR5021	Supercontig_2.3	100	16	0	0	0.036	32.2	*S. cerevisiae*	
ath-miR5021	Supercontig_2.3	100	16	0	0	0.036	32.2	*S. cerevisiae*	
ath-miR5021	Supercontig_2.3	100	16	0	0	0.036	32.2	*S. cerevisiae*	
ath-miR5021	Supercontig_2.3	100	16	0	0	0.036	32.2	*S. cerevisiae*	
ath-miR5021	Supercontig_2.3	100	16	0	0	0.036	32.2	*S. cerevisiae*	
ath-miR5021	Supercontig_2.3	100	16	0	0	0.036	32.2	*S. cerevisiae*	
ath-miR5021	Supercontig_2.3	100	16	0	0	0.036	32.2	*S. cerevisiae*	
ath-miR5021	Supercontig_2.3	100	16	0	0	0.036	32.2	*S. cerevisiae*	
ath-miR5021	Supercontig_2.3	100	16	0	0	0.036	32.2	*S. cerevisiae*	
ath-miR5021	Supercontig_2.3	100	16	0	0	0.036	32.2	*S. cerevisiae*	
ath-miR5021	Supercontig_2.3	100	16	0	0	0.036	32.2	*S. cerevisiae*	
ath-miR5021	Supercontig_2.3	100	16	0	0	0.036	32.2	*S. cerevisiae*	
ath-miR5021	Supercontig_2.3	100	16	0	0	0.036	32.2	*S. cerevisiae*	
ath-miR5021	Supercontig_2.3	100	16	0	0	0.036	32.2	*S. cerevisiae*	

Query id[a]	Subject id[b]	% Identity[c]	Alignment length[d]	Mismatches[e]	Gap opens[f]	Evalue [g]	Bit score[h]	Fungi vesicle[i]	Reference[j]
ath-miR5021	Supercontig_2.3	100	16	0	0	0.036	32.2	*S. cerevisiae*	
ath-miR5021	Supercontig_2.3	100	16	0	0	0.036	32.2	*S. cerevisiae*	
ath-miR5021	Supercontig_2.3	100	16	0	0	0.036	32.2	*S. cerevisiae*	
ath-miR5021	Supercontig_2.3	100	16	0	0	0.036	32.2	*S. cerevisiae*	
ath-miR5021	Supercontig_2.3	100	16	0	0	0.036	32.2	*S. cerevisiae*	
ath-miR5021	Supercontig_2.3	100	16	0	0	0.036	32.2	*S. cerevisiae*	
ath-miR5021	Supercontig_2.3	100	16	0	0	0.036	32.2	*S. cerevisiae*	
ath-miR5021	Supercontig_2.3	100	16	0	0	0.036	32.2	*S. cerevisiae*	
ath-miR5021	Supercontig_2.3	100	16	0	0	0.036	32.2	*S. cerevisiae*	
ath-miR5021	Supercontig_2.3	100	16	0	0	0.036	32.2	*S. cerevisiae*	
ath-miR5021	Supercontig_2.3	100	16	0	0	0.036	32.2	*S. cerevisiae*	
ath-miR5021	Supercontig_2.3	100	16	0	0	0.036	32.2	*S. cerevisiae*	
ath-miR5021	Supercontig_2.3	100	16	0	0	0.036	32.2	*S. cerevisiae*	
ath-miR5021	Supercontig_2.3	100	16	0	0	0.036	32.2	*S. cerevisiae*	
ath-miR5021	Supercontig_2.3	100	16	0	0	0.036	32.2	*S. cerevisiae*	
ath-miR5021	Supercontig_2.3	100	16	0	0	0.036	32.2	*S. cerevisiae*	
ath-miR5021	Supercontig_2.3	100	16	0	0	0.036	32.2	*S. cerevisiae*	
ath-miR5021	Supercontig_2.3	100	16	0	0	0.036	32.2	*S. cerevisiae*	
ath-miR5021	Supercontig_2.3	100	16	0	0	0.036	32.2	*S. cerevisiae*	
ath-miR5021	Supercontig_2.3	100	16	0	0	0.036	32.2	*S. cerevisiae*	
ath-miR5021	Supercontig_2.3	100	16	0	0	0.036	32.2	*S. cerevisiae*	
ath-miR5021	Supercontig_2.3	100	16	0	0	0.036	32.2	*S. cerevisiae*	
ath-miR5021	Supercontig_2.3	100	16	0	0	0.036	32.2	*S. cerevisiae*	
ath-miR5021	Supercontig_2.3	100	16	0	0	0.036	32.2	*S. cerevisiae*	
ath-miR5021	Supercontig_2.3	100	16	0	0	0.036	32.2	*S. cerevisiae*	
ath-miR5021	Supercontig_2.3	100	16	0	0	0.036	32.2	*S. cerevisiae*	
ath-miR5021	Supercontig_2.3	100	16	0	0	0.036	32.2	*S. cerevisiae*	
ath-miR5021	Supercontig_2.3	100	16	0	0	0.036	32.2	*S. cerevisiae*	
ath-miR5021	Supercontig_2.3	100	16	0	0	0.036	32.2	*S. cerevisiae*	
ath-miR5021	Supercontig_2.3	100	16	0	0	0.036	32.2	*S. cerevisiae*	
ath-miR5021	Supercontig_2.3	100	16	0	0	0.036	32.2	*S. cerevisiae*	
ath-miR5021	Supercontig_2.3	100	16	0	0	0.036	32.2	*S. cerevisiae*	
ath-miR5021	Supercontig_2.3	100	16	0	0	0.036	32.2	*S. cerevisiae*	
ath-miR5021	Supercontig_2.3	100	16	0	0	0.036	32.2	*S. cerevisiae*	
ath-miR5021	Supercontig_2.3	100	16	0	0	0.036	32.2	*S. cerevisiae*	
ath-miR5021	Supercontig_2.3	100	16	0	0	0.036	32.2	*S. cerevisiae*	
ath-miR5021	Supercontig_2.3	100	16	0	0	0.036	32.2	*S. cerevisiae*	
ath-miR5021	Supercontig_2.3	100	16	0	0	0.036	32.2	*S. cerevisiae*	
ath-miR5021	Supercontig_2.3	100	16	0	0	0.036	32.2	*S. cerevisiae*	
ath-miR5021	Supercontig_2.3	100	16	0	0	0.036	32.2	*S. cerevisiae*	
ath-miR5021	Supercontig_2.3	100	16	0	0	0.036	32.2	*S. cerevisiae*	
ath-miR5021	Supercontig_2.3	100	16	0	0	0.036	32.2	*S. cerevisiae*	
ath-miR5021	Supercontig_2.3	100	16	0	0	0.036	32.2	*S. cerevisiae*	
ath-miR5021	Supercontig_2.3	100	16	0	0	0.036	32.2	*S. cerevisiae*	
ath-miR5021	Supercontig_2.3	100	16	0	0	0.036	32.2	*S. cerevisiae*	
ath-miR5021	Supercontig_2.3	100	16	0	0	0.036	32.2	*S. cerevisiae*	

Query id[a]	Subject id[b]	% Identity[c]	Alignment length[d]	Mismatches[e]	Gap opens[f]	Evalue [g]	Bit score[h]	Fungi vesicle[i]	Reference[j]
ath-miR5021	Supercontig_2.3	100	16	0	0	0.036	32.2	*S. cerevisiae*	
ath-miR5021	Supercontig_2.3	100	16	0	0	0.036	32.2	*S. cerevisiae*	
ath-miR5021	Supercontig_2.3	100	16	0	0	0.036	32.2	*S. cerevisiae*	
ath-miR5021	Supercontig_2.3	100	16	0	0	0.036	32.2	*S. cerevisiae*	
ath-miR5021	Supercontig_2.3	100	16	0	0	0.036	32.2	*S. cerevisiae*	
ath-miR5021	Supercontig_2.3	100	16	0	0	0.036	32.2	*S. cerevisiae*	
ath-miR5021	Supercontig_2.3	100	17	0	0	0.009	34.2	*S. cerevisiae*	
ath-miR5021	Supercontig_2.3	100	17	0	0	0.009	34.2	*S. cerevisiae*	
ath-miR5021	Supercontig_2.3	100	17	0	0	0.009	34.2	*S. cerevisiae*	
ath-miR5021	Supercontig_2.3	100	17	0	0	0.009	34.2	*S. cerevisiae*	
ath-miR5021	Supercontig_2.3	100	17	0	0	0.009	34.2	*S. cerevisiae*	
ath-miR5021	Supercontig_2.3	100	17	0	0	0.009	34.2	*S. cerevisiae*	
ath-miR5021	Supercontig_2.3	100	17	0	0	0.009	34.2	*S. cerevisiae*	
ath-miR5021	Supercontig_2.3	100	17	0	0	0.009	34.2	*S. cerevisiae*	
ath-miR5021	Supercontig_2.3	100	17	0	0	0.009	34.2	*S. cerevisiae*	
ath-miR5021	Supercontig_2.3	100	17	0	0	0.009	34.2	*S. cerevisiae*	
ath-miR5021	Supercontig_2.3	100	17	0	0	0.009	34.2	*S. cerevisiae*	
ath-miR5021	Supercontig_2.3	100	17	0	0	0.009	34.2	*S. cerevisiae*	
ath-miR5021	Supercontig_2.3	100	17	0	0	0.009	34.2	*S. cerevisiae*	
ath-miR5021	Supercontig_2.3	100	18	0	0	0.002	36.2	*S. cerevisiae*	
ath-miR5021	Supercontig_2.3	100	18	0	0	0.002	36.2	*S. cerevisiae*	
ath-miR5021	Supercontig_2.3	100	18	0	0	0.002	36.2	*S. cerevisiae*	
ath-miR5021	Supercontig_2.3	100	18	0	0	0.002	36.2	*S. cerevisiae*	
ath-miR5021	Supercontig_2.3	100	18	0	0	0.002	36.2	*S. cerevisiae*	
ath-miR5021	Supercontig_2.3	100	18	0	0	0.002	36.2	*S. cerevisiae*	
ath-miR5021	Supercontig_2.3	100	18	0	0	0.002	36.2	*S. cerevisiae*	
ath-miR5021	Supercontig_2.3	100	18	0	0	0.002	36.2	*S. cerevisiae*	
ath-miR5021	Supercontig_2.3	100	18	0	0	0.002	36.2	*S. cerevisiae*	
ath-miR5021	Supercontig_2.3	100	18	0	0	0.002	36.2	*S. cerevisiae*	
ath-miR5021	Supercontig_2.3	100	19	0	0	6.00E-04	38.2	*S. cerevisiae*	
ath-miR5021	Supercontig_2.3	95	20	1	0	0.036	32.2	*S. cerevisiae*	
ath-miR5021	Supercontig_2.4	100	16	0	0	0.036	32.2	*S. cerevisiae*	
ath-miR5021	Supercontig_2.4	100	16	0	0	0.036	32.2	*S. cerevisiae*	
ath-miR5021	Supercontig_2.4	100	16	0	0	0.036	32.2	*S. cerevisiae*	
ath-miR5021	Supercontig_2.4	100	16	0	0	0.036	32.2	*S. cerevisiae*	
ath-miR5021	Supercontig_2.4	100	16	0	0	0.036	32.2	*S. cerevisiae*	
ath-miR5021	Supercontig_2.4	100	16	0	0	0.036	32.2	*S. cerevisiae*	
ath-miR5021	Supercontig_2.4	100	16	0	0	0.036	32.2	*S. cerevisiae*	
ath-miR5021	Supercontig_2.4	100	16	0	0	0.036	32.2	*S. cerevisiae*	
ath-miR5021	Supercontig_2.4	100	16	0	0	0.036	32.2	*S. cerevisiae*	
ath-miR5021	Supercontig_2.4	100	16	0	0	0.036	32.2	*S. cerevisiae*	
ath-miR5021	Supercontig_2.4	100	16	0	0	0.036	32.2	*S. cerevisiae*	
ath-miR5021	Supercontig_2.4	100	16	0	0	0.036	32.2	*S. cerevisiae*	
ath-miR5021	Supercontig_2.4	100	16	0	0	0.036	32.2	*S. cerevisiae*	
ath-miR5021	Supercontig_2.4	100	16	0	0	0.036	32.2	*S. cerevisiae*	
ath-miR5021	Supercontig_2.4	100	16	0	0	0.036	32.2	*S. cerevisiae*	

Query id[a]	Subject id[b]	% Identity[c]	Alignment length[d]	Mismatches[e]	Gap opens[f]	Evalue [g]	Bit score[h]	Fungi vesicle[i]	Reference[j]
ath-miR5021	Supercontig_2.4	100	16	0	0	0.036	32.2	*S. cerevisiae*	
ath-miR5021	Supercontig_2.4	100	16	0	0	0.036	32.2	*S. cerevisiae*	
ath-miR5021	Supercontig_2.4	100	16	0	0	0.036	32.2	*S. cerevisiae*	
ath-miR5021	Supercontig_2.4	100	16	0	0	0.036	32.2	*S. cerevisiae*	
ath-miR5021	Supercontig_2.4	100	16	0	0	0.036	32.2	*S. cerevisiae*	
ath-miR5021	Supercontig_2.4	100	16	0	0	0.036	32.2	*S. cerevisiae*	
ath-miR5021	Supercontig_2.4	100	16	0	0	0.036	32.2	*S. cerevisiae*	
ath-miR5021	Supercontig_2.4	100	16	0	0	0.036	32.2	*S. cerevisiae*	
ath-miR5021	Supercontig_2.4	100	16	0	0	0.036	32.2	*S. cerevisiae*	
ath-miR5021	Supercontig_2.4	100	16	0	0	0.036	32.2	*S. cerevisiae*	
ath-miR5021	Supercontig_2.4	100	16	0	0	0.036	32.2	*S. cerevisiae*	
ath-miR5021	Supercontig_2.4	100	16	0	0	0.036	32.2	*S. cerevisiae*	
ath-miR5021	Supercontig_2.4	100	16	0	0	0.036	32.2	*S. cerevisiae*	
ath-miR5021	Supercontig_2.4	100	16	0	0	0.036	32.2	*S. cerevisiae*	
ath-miR5021	Supercontig_2.4	100	16	0	0	0.036	32.2	*S. cerevisiae*	
ath-miR5021	Supercontig_2.4	100	16	0	0	0.036	32.2	*S. cerevisiae*	
ath-miR5021	Supercontig_2.4	100	16	0	0	0.036	32.2	*S. cerevisiae*	
ath-miR5021	Supercontig_2.4	100	16	0	0	0.036	32.2	*S. cerevisiae*	
ath-miR5021	Supercontig_2.4	100	16	0	0	0.036	32.2	*S. cerevisiae*	
ath-miR5021	Supercontig_2.4	100	16	0	0	0.036	32.2	*S. cerevisiae*	
ath-miR5021	Supercontig_2.4	100	16	0	0	0.036	32.2	*S. cerevisiae*	
ath-miR5021	Supercontig_2.4	100	16	0	0	0.036	32.2	*S. cerevisiae*	
ath-miR5021	Supercontig_2.4	100	16	0	0	0.036	32.2	*S. cerevisiae*	
ath-miR5021	Supercontig_2.4	100	16	0	0	0.036	32.2	*S. cerevisiae*	
ath-miR5021	Supercontig_2.4	100	16	0	0	0.036	32.2	*S. cerevisiae*	
ath-miR5021	Supercontig_2.4	100	16	0	0	0.036	32.2	*S. cerevisiae*	
ath-miR5021	Supercontig_2.4	100	16	0	0	0.036	32.2	*S. cerevisiae*	
ath-miR5021	Supercontig_2.4	100	16	0	0	0.036	32.2	*S. cerevisiae*	
ath-miR5021	Supercontig_2.4	100	16	0	0	0.036	32.2	*S. cerevisiae*	
ath-miR5021	Supercontig_2.4	100	16	0	0	0.036	32.2	*S. cerevisiae*	
ath-miR5021	Supercontig_2.4	100	16	0	0	0.036	32.2	*S. cerevisiae*	
ath-miR5021	Supercontig_2.4	100	16	0	0	0.036	32.2	*S. cerevisiae*	
ath-miR5021	Supercontig_2.4	100	16	0	0	0.036	32.2	*S. cerevisiae*	
ath-miR5021	Supercontig_2.4	100	16	0	0	0.036	32.2	*S. cerevisiae*	
ath-miR5021	Supercontig_2.4	100	16	0	0	0.036	32.2	*S. cerevisiae*	
ath-miR5021	Supercontig_2.4	100	16	0	0	0.036	32.2	*S. cerevisiae*	
ath-miR5021	Supercontig_2.4	100	16	0	0	0.036	32.2	*S. cerevisiae*	
ath-miR5021	Supercontig_2.4	100	16	0	0	0.036	32.2	*S. cerevisiae*	
ath-miR5021	Supercontig_2.4	100	16	0	0	0.036	32.2	*S. cerevisiae*	
ath-miR5021	Supercontig_2.4	100	16	0	0	0.036	32.2	*S. cerevisiae*	
ath-miR5021	Supercontig_2.4	100	16	0	0	0.036	32.2	*S. cerevisiae*	
ath-miR5021	Supercontig_2.4	100	16	0	0	0.036	32.2	*S. cerevisiae*	
ath-miR5021	Supercontig_2.4	100	16	0	0	0.036	32.2	*S. cerevisiae*	
ath-miR5021	Supercontig_2.4	100	16	0	0	0.036	32.2	*S. cerevisiae*	
ath-miR5021	Supercontig_2.4	100	16	0	0	0.036	32.2	*S. cerevisiae*	
ath-miR5021	Supercontig_2.4	100	16	0	0	0.036	32.2	*S. cerevisiae*	

Query id[a]	Subject id[b]	% Identity[c]	Alignment length[d]	Mismatches[e]	Gap opens[f]	Evalue [g]	Bit score[h]	Fungi vesicle[i]	Reference[j]
ath-miR5021	Supercontig_2.4	100	16	0	0	0.036	32.2	*S. cerevisiae*	
ath-miR5021	Supercontig_2.4	100	16	0	0	0.036	32.2	*S. cerevisiae*	
ath-miR5021	Supercontig_2.4	100	16	0	0	0.036	32.2	*S. cerevisiae*	
ath-miR5021	Supercontig_2.4	100	16	0	0	0.036	32.2	*S. cerevisiae*	
ath-miR5021	Supercontig_2.4	100	16	0	0	0.036	32.2	*S. cerevisiae*	
ath-miR5021	Supercontig_2.4	100	16	0	0	0.036	32.2	*S. cerevisiae*	
ath-miR5021	Supercontig_2.4	100	16	0	0	0.036	32.2	*S. cerevisiae*	
ath-miR5021	Supercontig_2.4	100	16	0	0	0.036	32.2	*S. cerevisiae*	
ath-miR5021	Supercontig_2.4	100	16	0	0	0.036	32.2	*S. cerevisiae*	
ath-miR5021	Supercontig_2.4	100	16	0	0	0.036	32.2	*S. cerevisiae*	
ath-miR5021	Supercontig_2.4	100	16	0	0	0.036	32.2	*S. cerevisiae*	
ath-miR5021	Supercontig_2.4	100	16	0	0	0.036	32.2	*S. cerevisiae*	
ath-miR5021	Supercontig_2.4	100	16	0	0	0.036	32.2	*S. cerevisiae*	
ath-miR5021	Supercontig_2.4	100	16	0	0	0.036	32.2	*S. cerevisiae*	
ath-miR5021	Supercontig_2.4	100	16	0	0	0.036	32.2	*S. cerevisiae*	
ath-miR5021	Supercontig_2.4	100	16	0	0	0.036	32.2	*S. cerevisiae*	
ath-miR5021	Supercontig_2.4	100	16	0	0	0.036	32.2	*S. cerevisiae*	
ath-miR5021	Supercontig_2.4	100	16	0	0	0.036	32.2	*S. cerevisiae*	
ath-miR5021	Supercontig_2.4	100	16	0	0	0.036	32.2	*S. cerevisiae*	
ath-miR5021	Supercontig_2.4	100	16	0	0	0.036	32.2	*S. cerevisiae*	
ath-miR5021	Supercontig_2.4	100	16	0	0	0.036	32.2	*S. cerevisiae*	
ath-miR5021	Supercontig_2.4	100	16	0	0	0.036	32.2	*S. cerevisiae*	
ath-miR5021	Supercontig_2.4	100	16	0	0	0.036	32.2	*S. cerevisiae*	
ath-miR5021	Supercontig_2.4	100	16	0	0	0.036	32.2	*S. cerevisiae*	
ath-miR5021	Supercontig_2.4	100	16	0	0	0.036	32.2	*S. cerevisiae*	
ath-miR5021	Supercontig_2.4	100	16	0	0	0.036	32.2	*S. cerevisiae*	
ath-miR5021	Supercontig_2.4	100	16	0	0	0.036	32.2	*S. cerevisiae*	
ath-miR5021	Supercontig_2.4	100	16	0	0	0.036	32.2	*S. cerevisiae*	
ath-miR5021	Supercontig_2.4	100	16	0	0	0.036	32.2	*S. cerevisiae*	
ath-miR5021	Supercontig_2.4	100	16	0	0	0.036	32.2	*S. cerevisiae*	
ath-miR5021	Supercontig_2.4	100	16	0	0	0.036	32.2	*S. cerevisiae*	
ath-miR5021	Supercontig_2.4	100	16	0	0	0.036	32.2	*S. cerevisiae*	
ath-miR5021	Supercontig_2.4	100	16	0	0	0.036	32.2	*S. cerevisiae*	
ath-miR5021	Supercontig_2.4	100	16	0	0	0.036	32.2	*S. cerevisiae*	
ath-miR5021	Supercontig_2.4	100	16	0	0	0.036	32.2	*S. cerevisiae*	
ath-miR5021	Supercontig_2.4	100	16	0	0	0.036	32.2	*S. cerevisiae*	
ath-miR5021	Supercontig_2.4	100	16	0	0	0.036	32.2	*S. cerevisiae*	
ath-miR5021	Supercontig_2.4	100	16	0	0	0.036	32.2	*S. cerevisiae*	
ath-miR5021	Supercontig_2.4	100	16	0	0	0.036	32.2	*S. cerevisiae*	
ath-miR5021	Supercontig_2.4	100	16	0	0	0.036	32.2	*S. cerevisiae*	
ath-miR5021	Supercontig_2.4	100	16	0	0	0.036	32.2	*S. cerevisiae*	
ath-miR5021	Supercontig_2.4	100	16	0	0	0.036	32.2	*S. cerevisiae*	
ath-miR5021	Supercontig_2.4	100	16	0	0	0.036	32.2	*S. cerevisiae*	
ath-miR5021	Supercontig_2.4	100	16	0	0	0.036	32.2	*S. cerevisiae*	
ath-miR5021	Supercontig_2.4	100	16	0	0	0.036	32.2	*S. cerevisiae*	
ath-miR5021	Supercontig_2.4	100	16	0	0	0.036	32.2	*S. cerevisiae*	

Query id[a]	Subject id[b]	% Identity[c]	Alignment length[d]	Mismatches[e]	Gap opens[f]	Evalue [g]	Bit score[h]	Fungi vesicle[i]	Reference[j]
ath-miR5021	Supercontig_2.4	100	16	0	0	0.036	32.2	*S. cerevisiae*	
ath-miR5021	Supercontig_2.4	100	16	0	0	0.036	32.2	*S. cerevisiae*	
ath-miR5021	Supercontig_2.4	100	16	0	0	0.036	32.2	*S. cerevisiae*	
ath-miR5021	Supercontig_2.4	100	16	0	0	0.036	32.2	*S. cerevisiae*	
ath-miR5021	Supercontig_2.4	100	16	0	0	0.036	32.2	*S. cerevisiae*	
ath-miR5021	Supercontig_2.4	100	16	0	0	0.036	32.2	*S. cerevisiae*	
ath-miR5021	Supercontig_2.4	100	16	0	0	0.036	32.2	*S. cerevisiae*	
ath-miR5021	Supercontig_2.4	100	16	0	0	0.036	32.2	*S. cerevisiae*	
ath-miR5021	Supercontig_2.4	100	16	0	0	0.036	32.2	*S. cerevisiae*	
ath-miR5021	Supercontig_2.4	100	16	0	0	0.036	32.2	*S. cerevisiae*	
ath-miR5021	Supercontig_2.4	100	16	0	0	0.036	32.2	*S. cerevisiae*	
ath-miR5021	Supercontig_2.4	100	16	0	0	0.036	32.2	*S. cerevisiae*	
ath-miR5021	Supercontig_2.4	100	16	0	0	0.036	32.2	*S. cerevisiae*	
ath-miR5021	Supercontig_2.4	100	16	0	0	0.036	32.2	*S. cerevisiae*	
ath-miR5021	Supercontig_2.4	100	16	0	0	0.036	32.2	*S. cerevisiae*	
ath-miR5021	Supercontig_2.4	100	16	0	0	0.036	32.2	*S. cerevisiae*	
ath-miR5021	Supercontig_2.4	100	16	0	0	0.036	32.2	*S. cerevisiae*	
ath-miR5021	Supercontig_2.4	100	16	0	0	0.036	32.2	*S. cerevisiae*	
ath-miR5021	Supercontig_2.4	100	16	0	0	0.036	32.2	*S. cerevisiae*	
ath-miR5021	Supercontig_2.4	100	16	0	0	0.036	32.2	*S. cerevisiae*	
ath-miR5021	Supercontig_2.4	100	16	0	0	0.036	32.2	*S. cerevisiae*	
ath-miR5021	Supercontig_2.4	100	16	0	0	0.036	32.2	*S. cerevisiae*	
ath-miR5021	Supercontig_2.4	100	16	0	0	0.036	32.2	*S. cerevisiae*	
ath-miR5021	Supercontig_2.4	100	16	0	0	0.036	32.2	*S. cerevisiae*	
ath-miR5021	Supercontig_2.4	100	16	0	0	0.036	32.2	*S. cerevisiae*	
ath-miR5021	Supercontig_2.4	100	16	0	0	0.036	32.2	*S. cerevisiae*	
ath-miR5021	Supercontig_2.4	100	16	0	0	0.036	32.2	*S. cerevisiae*	
ath-miR5021	Supercontig_2.4	100	16	0	0	0.036	32.2	*S. cerevisiae*	
ath-miR5021	Supercontig_2.4	100	16	0	0	0.036	32.2	*S. cerevisiae*	
ath-miR5021	Supercontig_2.4	100	16	0	0	0.036	32.2	*S. cerevisiae*	
ath-miR5021	Supercontig_2.4	100	17	0	0	0.009	34.2	*S. cerevisiae*	
ath-miR5021	Supercontig_2.4	100	17	0	0	0.009	34.2	*S. cerevisiae*	
ath-miR5021	Supercontig_2.4	100	17	0	0	0.009	34.2	*S. cerevisiae*	
ath-miR5021	Supercontig_2.4	100	17	0	0	0.009	34.2	*S. cerevisiae*	
ath-miR5021	Supercontig_2.4	100	17	0	0	0.009	34.2	*S. cerevisiae*	
ath-miR5021	Supercontig_2.4	100	17	0	0	0.009	34.2	*S. cerevisiae*	
ath-miR5021	Supercontig_2.4	100	18	0	0	0.002	36.2	*S. cerevisiae*	
ath-miR5021	Supercontig_2.4	100	18	0	0	0.002	36.2	*S. cerevisiae*	
ath-miR5021	Supercontig_2.4	100	18	0	0	0.002	36.2	*S. cerevisiae*	
ath-miR5021	Supercontig_2.4	100	18	0	0	0.002	36.2	*S. cerevisiae*	
ath-miR5021	Supercontig_2.4	100	18	0	0	0.002	36.2	*S. cerevisiae*	
ath-miR5021	Supercontig_2.4	100	18	0	0	0.002	36.2	*S. cerevisiae*	
ath-miR5021	Supercontig_2.4	100	18	0	0	0.002	36.2	*S. cerevisiae*	
ath-miR5021	Supercontig_2.5	100	16	0	0	0.036	32.2	*S. cerevisiae*	
ath-miR5021	Supercontig_2.5	100	16	0	0	0.036	32.2	*S. cerevisiae*	
ath-miR5021	Supercontig_2.5	100	16	0	0	0.036	32.2	*S. cerevisiae*	

Query id[a]	Subject id[b]	% Identity[c]	Alignment length[d]	Mismatches[e]	Gap opens[f]	Evalue [g]	Bit score[h]	Fungi vesicle[i]	Reference[j]
ath-miR5021	Supercontig_2.5	100	16	0	0	0.036	32.2	*S. cerevisiae*	
ath-miR5021	Supercontig_2.5	100	16	0	0	0.036	32.2	*S. cerevisiae*	
ath-miR5021	Supercontig_2.5	100	16	0	0	0.036	32.2	*S. cerevisiae*	
ath-miR5021	Supercontig_2.5	100	16	0	0	0.036	32.2	*S. cerevisiae*	
ath-miR5021	Supercontig_2.5	100	16	0	0	0.036	32.2	*S. cerevisiae*	
ath-miR5021	Supercontig_2.5	100	16	0	0	0.036	32.2	*S. cerevisiae*	
ath-miR5021	Supercontig_2.5	100	16	0	0	0.036	32.2	*S. cerevisiae*	
ath-miR5021	Supercontig_2.5	100	16	0	0	0.036	32.2	*S. cerevisiae*	
ath-miR5021	Supercontig_2.5	100	16	0	0	0.036	32.2	*S. cerevisiae*	
ath-miR5021	Supercontig_2.5	100	16	0	0	0.036	32.2	*S. cerevisiae*	
ath-miR5021	Supercontig_2.5	100	16	0	0	0.036	32.2	*S. cerevisiae*	
ath-miR5021	Supercontig_2.5	100	16	0	0	0.036	32.2	*S. cerevisiae*	
ath-miR5021	Supercontig_2.5	100	16	0	0	0.036	32.2	*S. cerevisiae*	
ath-miR5021	Supercontig_2.5	100	16	0	0	0.036	32.2	*S. cerevisiae*	
ath-miR5021	Supercontig_2.5	100	16	0	0	0.036	32.2	*S. cerevisiae*	
ath-miR5021	Supercontig_2.5	100	16	0	0	0.036	32.2	*S. cerevisiae*	
ath-miR5021	Supercontig_2.5	100	16	0	0	0.036	32.2	*S. cerevisiae*	
ath-miR5021	Supercontig_2.5	100	16	0	0	0.036	32.2	*S. cerevisiae*	
ath-miR5021	Supercontig_2.5	100	16	0	0	0.036	32.2	*S. cerevisiae*	
ath-miR5021	Supercontig_2.5	100	16	0	0	0.036	32.2	*S. cerevisiae*	
ath-miR5021	Supercontig_2.5	100	16	0	0	0.036	32.2	*S. cerevisiae*	
ath-miR5021	Supercontig_2.5	100	16	0	0	0.036	32.2	*S. cerevisiae*	
ath-miR5021	Supercontig_2.5	100	16	0	0	0.036	32.2	*S. cerevisiae*	
ath-miR5021	Supercontig_2.5	100	16	0	0	0.036	32.2	*S. cerevisiae*	
ath-miR5021	Supercontig_2.5	100	16	0	0	0.036	32.2	*S. cerevisiae*	
ath-miR5021	Supercontig_2.5	100	16	0	0	0.036	32.2	*S. cerevisiae*	
ath-miR5021	Supercontig_2.5	100	16	0	0	0.036	32.2	*S. cerevisiae*	
ath-miR5021	Supercontig_2.5	100	16	0	0	0.036	32.2	*S. cerevisiae*	
ath-miR5021	Supercontig_2.5	100	16	0	0	0.036	32.2	*S. cerevisiae*	
ath-miR5021	Supercontig_2.5	100	16	0	0	0.036	32.2	*S. cerevisiae*	
ath-miR5021	Supercontig_2.5	100	16	0	0	0.036	32.2	*S. cerevisiae*	
ath-miR5021	Supercontig_2.5	100	16	0	0	0.036	32.2	*S. cerevisiae*	
ath-miR5021	Supercontig_2.5	100	16	0	0	0.036	32.2	*S. cerevisiae*	
ath-miR5021	Supercontig_2.5	100	16	0	0	0.036	32.2	*S. cerevisiae*	
ath-miR5021	Supercontig_2.5	100	16	0	0	0.036	32.2	*S. cerevisiae*	
ath-miR5021	Supercontig_2.5	100	16	0	0	0.036	32.2	*S. cerevisiae*	
ath-miR5021	Supercontig_2.5	100	16	0	0	0.036	32.2	*S. cerevisiae*	
ath-miR5021	Supercontig_2.5	100	16	0	0	0.036	32.2	*S. cerevisiae*	
ath-miR5021	Supercontig_2.5	100	16	0	0	0.036	32.2	*S. cerevisiae*	
ath-miR5021	Supercontig_2.5	100	16	0	0	0.036	32.2	*S. cerevisiae*	
ath-miR5021	Supercontig_2.5	100	16	0	0	0.036	32.2	*S. cerevisiae*	
ath-miR5021	Supercontig_2.5	100	16	0	0	0.036	32.2	*S. cerevisiae*	
ath-miR5021	Supercontig_2.5	100	16	0	0	0.036	32.2	*S. cerevisiae*	
ath-miR5021	Supercontig_2.5	100	16	0	0	0.036	32.2	*S. cerevisiae*	
ath-miR5021	Supercontig_2.5	100	16	0	0	0.036	32.2	*S. cerevisiae*	
ath-miR5021	Supercontig_2.5	100	16	0	0	0.036	32.2	*S. cerevisiae*	

Query id[a]	Subject id[b]	% Identity[c]	Alignment length[d]	Mismatches[e]	Gap opens[f]	Evalue [g]	Bit score[h]	Fungi vesicle[i]	Reference[j]
ath-miR5021	Supercontig_2.5	100	16	0	0	0.036	32.2	*S. cerevisiae*	
ath-miR5021	Supercontig_2.5	100	16	0	0	0.036	32.2	*S. cerevisiae*	
ath-miR5021	Supercontig_2.5	100	16	0	0	0.036	32.2	*S. cerevisiae*	
ath-miR5021	Supercontig_2.5	100	16	0	0	0.036	32.2	*S. cerevisiae*	
ath-miR5021	Supercontig_2.5	100	16	0	0	0.036	32.2	*S. cerevisiae*	
ath-miR5021	Supercontig_2.5	100	16	0	0	0.036	32.2	*S. cerevisiae*	
ath-miR5021	Supercontig_2.5	100	16	0	0	0.036	32.2	*S. cerevisiae*	
ath-miR5021	Supercontig_2.5	100	16	0	0	0.036	32.2	*S. cerevisiae*	
ath-miR5021	Supercontig_2.5	100	16	0	0	0.036	32.2	*S. cerevisiae*	
ath-miR5021	Supercontig_2.5	100	16	0	0	0.036	32.2	*S. cerevisiae*	
ath-miR5021	Supercontig_2.5	100	16	0	0	0.036	32.2	*S. cerevisiae*	
ath-miR5021	Supercontig_2.5	100	16	0	0	0.036	32.2	*S. cerevisiae*	
ath-miR5021	Supercontig_2.5	100	16	0	0	0.036	32.2	*S. cerevisiae*	
ath-miR5021	Supercontig_2.5	100	16	0	0	0.036	32.2	*S. cerevisiae*	
ath-miR5021	Supercontig_2.5	100	16	0	0	0.036	32.2	*S. cerevisiae*	
ath-miR5021	Supercontig_2.5	100	16	0	0	0.036	32.2	*S. cerevisiae*	
ath-miR5021	Supercontig_2.5	100	16	0	0	0.036	32.2	*S. cerevisiae*	
ath-miR5021	Supercontig_2.5	100	16	0	0	0.036	32.2	*S. cerevisiae*	
ath-miR5021	Supercontig_2.5	100	16	0	0	0.036	32.2	*S. cerevisiae*	
ath-miR5021	Supercontig_2.5	100	16	0	0	0.036	32.2	*S. cerevisiae*	
ath-miR5021	Supercontig_2.5	100	16	0	0	0.036	32.2	*S. cerevisiae*	
ath-miR5021	Supercontig_2.5	100	16	0	0	0.036	32.2	*S. cerevisiae*	
ath-miR5021	Supercontig_2.5	100	16	0	0	0.036	32.2	*S. cerevisiae*	
ath-miR5021	Supercontig_2.5	100	16	0	0	0.036	32.2	*S. cerevisiae*	
ath-miR5021	Supercontig_2.5	100	16	0	0	0.036	32.2	*S. cerevisiae*	
ath-miR5021	Supercontig_2.5	100	16	0	0	0.036	32.2	*S. cerevisiae*	
ath-miR5021	Supercontig_2.5	100	16	0	0	0.036	32.2	*S. cerevisiae*	
ath-miR5021	Supercontig_2.5	100	16	0	0	0.036	32.2	*S. cerevisiae*	
ath-miR5021	Supercontig_2.5	100	16	0	0	0.036	32.2	*S. cerevisiae*	
ath-miR5021	Supercontig_2.5	100	16	0	0	0.036	32.2	*S. cerevisiae*	
ath-miR5021	Supercontig_2.5	100	16	0	0	0.036	32.2	*S. cerevisiae*	
ath-miR5021	Supercontig_2.5	100	16	0	0	0.036	32.2	*S. cerevisiae*	
ath-miR5021	Supercontig_2.5	100	16	0	0	0.036	32.2	*S. cerevisiae*	
ath-miR5021	Supercontig_2.5	100	16	0	0	0.036	32.2	*S. cerevisiae*	
ath-miR5021	Supercontig_2.5	100	16	0	0	0.036	32.2	*S. cerevisiae*	
ath-miR5021	Supercontig_2.5	100	16	0	0	0.036	32.2	*S. cerevisiae*	
ath-miR5021	Supercontig_2.5	100	16	0	0	0.036	32.2	*S. cerevisiae*	
ath-miR5021	Supercontig_2.5	100	16	0	0	0.036	32.2	*S. cerevisiae*	
ath-miR5021	Supercontig_2.5	100	16	0	0	0.036	32.2	*S. cerevisiae*	
ath-miR5021	Supercontig_2.5	100	16	0	0	0.036	32.2	*S. cerevisiae*	
ath-miR5021	Supercontig_2.5	100	16	0	0	0.036	32.2	*S. cerevisiae*	
ath-miR5021	Supercontig_2.5	100	16	0	0	0.036	32.2	*S. cerevisiae*	
ath-miR5021	Supercontig_2.5	100	16	0	0	0.036	32.2	*S. cerevisiae*	
ath-miR5021	Supercontig_2.5	100	16	0	0	0.036	32.2	*S. cerevisiae*	
ath-miR5021	Supercontig_2.5	100	16	0	0	0.036	32.2	*S. cerevisiae*	
ath-miR5021	Supercontig_2.5	100	16	0	0	0.036	32.2	*S. cerevisiae*	

Query id[a]	Subject id[b]	% Identity[c]	Alignment length[d]	Mismatches[e]	Gap opens[f]	Evalue [g]	Bit score[h]	Fungi vesicle[i]	Reference[j]
ath-miR5021	Supercontig_2.5	100	16	0	0	0.036	32.2	*S. cerevisiae*	
ath-miR5021	Supercontig_2.5	100	16	0	0	0.036	32.2	*S. cerevisiae*	
ath-miR5021	Supercontig_2.5	100	16	0	0	0.036	32.2	*S. cerevisiae*	
ath-miR5021	Supercontig_2.5	100	16	0	0	0.036	32.2	*S. cerevisiae*	
ath-miR5021	Supercontig_2.5	100	16	0	0	0.036	32.2	*S. cerevisiae*	
ath-miR5021	Supercontig_2.5	100	16	0	0	0.036	32.2	*S. cerevisiae*	
ath-miR5021	Supercontig_2.5	100	16	0	0	0.036	32.2	*S. cerevisiae*	
ath-miR5021	Supercontig_2.5	100	16	0	0	0.036	32.2	*S. cerevisiae*	
ath-miR5021	Supercontig_2.5	100	16	0	0	0.036	32.2	*S. cerevisiae*	
ath-miR5021	Supercontig_2.5	100	17	0	0	0.009	34.2	*S. cerevisiae*	
ath-miR5021	Supercontig_2.5	100	17	0	0	0.009	34.2	*S. cerevisiae*	
ath-miR5021	Supercontig_2.5	100	17	0	0	0.009	34.2	*S. cerevisiae*	
ath-miR5021	Supercontig_2.5	100	17	0	0	0.009	34.2	*S. cerevisiae*	
ath-miR5021	Supercontig_2.5	100	17	0	0	0.009	34.2	*S. cerevisiae*	
ath-miR5021	Supercontig_2.5	100	17	0	0	0.009	34.2	*S. cerevisiae*	
ath-miR5021	Supercontig_2.5	100	17	0	0	0.009	34.2	*S. cerevisiae*	
ath-miR5021	Supercontig_2.5	100	17	0	0	0.009	34.2	*S. cerevisiae*	
ath-miR5021	Supercontig_2.5	100	17	0	0	0.009	34.2	*S. cerevisiae*	
ath-miR5021	Supercontig_2.5	100	18	0	0	0.002	36.2	*S. cerevisiae*	
ath-miR5021	Supercontig_2.5	100	18	0	0	0.002	36.2	*S. cerevisiae*	
ath-miR5021	Supercontig_2.5	100	18	0	0	0.002	36.2	*S. cerevisiae*	
ath-miR5021	Supercontig_2.5	100	18	0	0	0.002	36.2	*S. cerevisiae*	
ath-miR5021	Supercontig_2.6	100	16	0	0	0.036	32.2	*S. cerevisiae*	
ath-miR5021	Supercontig_2.6	100	16	0	0	0.036	32.2	*S. cerevisiae*	
ath-miR5021	Supercontig_2.6	100	16	0	0	0.036	32.2	*S. cerevisiae*	
ath-miR5021	Supercontig_2.6	100	16	0	0	0.036	32.2	*S. cerevisiae*	
ath-miR5021	Supercontig_2.6	100	16	0	0	0.036	32.2	*S. cerevisiae*	
ath-miR5021	Supercontig_2.6	100	16	0	0	0.036	32.2	*S. cerevisiae*	
ath-miR5021	Supercontig_2.6	100	16	0	0	0.036	32.2	*S. cerevisiae*	
ath-miR5021	Supercontig_2.6	100	16	0	0	0.036	32.2	*S. cerevisiae*	
ath-miR5021	Supercontig_2.6	100	16	0	0	0.036	32.2	*S. cerevisiae*	
ath-miR5021	Supercontig_2.6	100	16	0	0	0.036	32.2	*S. cerevisiae*	
ath-miR5021	Supercontig_2.6	100	16	0	0	0.036	32.2	*S. cerevisiae*	
ath-miR5021	Supercontig_2.6	100	16	0	0	0.036	32.2	*S. cerevisiae*	
ath-miR5021	Supercontig_2.6	100	16	0	0	0.036	32.2	*S. cerevisiae*	
ath-miR5021	Supercontig_2.6	100	16	0	0	0.036	32.2	*S. cerevisiae*	
ath-miR5021	Supercontig_2.6	100	16	0	0	0.036	32.2	*S. cerevisiae*	
ath-miR5021	Supercontig_2.6	100	16	0	0	0.036	32.2	*S. cerevisiae*	
ath-miR5021	Supercontig_2.6	100	16	0	0	0.036	32.2	*S. cerevisiae*	
ath-miR5021	Supercontig_2.6	100	16	0	0	0.036	32.2	*S. cerevisiae*	
ath-miR5021	Supercontig_2.6	100	16	0	0	0.036	32.2	*S. cerevisiae*	
ath-miR5021	Supercontig_2.6	100	16	0	0	0.036	32.2	*S. cerevisiae*	
ath-miR5021	Supercontig_2.6	100	16	0	0	0.036	32.2	*S. cerevisiae*	
ath-miR5021	Supercontig_2.6	100	16	0	0	0.036	32.2	*S. cerevisiae*	
ath-miR5021	Supercontig_2.6	100	16	0	0	0.036	32.2	*S. cerevisiae*	
ath-miR5021	Supercontig_2.6	100	16	0	0	0.036	32.2	*S. cerevisiae*	

Query id[a]	Subject id[b]	% Identity[c]	Alignment length[d]	Mismatches[e]	Gap opens[f]	Evalue [g]	Bit score[h]	Fungi vesicle[i]	Reference[j]
ath-miR5021	Supercontig_2.6	100	16	0	0	0.036	32.2	*S. cerevisiae*	
ath-miR5021	Supercontig_2.6	100	16	0	0	0.036	32.2	*S. cerevisiae*	
ath-miR5021	Supercontig_2.6	100	16	0	0	0.036	32.2	*S. cerevisiae*	
ath-miR5021	Supercontig_2.6	100	16	0	0	0.036	32.2	*S. cerevisiae*	
ath-miR5021	Supercontig_2.6	100	16	0	0	0.036	32.2	*S. cerevisiae*	
ath-miR5021	Supercontig_2.6	100	16	0	0	0.036	32.2	*S. cerevisiae*	
ath-miR5021	Supercontig_2.6	100	16	0	0	0.036	32.2	*S. cerevisiae*	
ath-miR5021	Supercontig_2.6	100	16	0	0	0.036	32.2	*S. cerevisiae*	
ath-miR5021	Supercontig_2.6	100	16	0	0	0.036	32.2	*S. cerevisiae*	
ath-miR5021	Supercontig_2.6	100	16	0	0	0.036	32.2	*S. cerevisiae*	
ath-miR5021	Supercontig_2.6	100	16	0	0	0.036	32.2	*S. cerevisiae*	
ath-miR5021	Supercontig_2.6	100	16	0	0	0.036	32.2	*S. cerevisiae*	
ath-miR5021	Supercontig_2.6	100	16	0	0	0.036	32.2	*S. cerevisiae*	
ath-miR5021	Supercontig_2.6	100	16	0	0	0.036	32.2	*S. cerevisiae*	
ath-miR5021	Supercontig_2.6	100	16	0	0	0.036	32.2	*S. cerevisiae*	
ath-miR5021	Supercontig_2.6	100	16	0	0	0.036	32.2	*S. cerevisiae*	
ath-miR5021	Supercontig_2.6	100	16	0	0	0.036	32.2	*S. cerevisiae*	
ath-miR5021	Supercontig_2.6	100	16	0	0	0.036	32.2	*S. cerevisiae*	
ath-miR5021	Supercontig_2.6	100	16	0	0	0.036	32.2	*S. cerevisiae*	
ath-miR5021	Supercontig_2.6	100	16	0	0	0.036	32.2	*S. cerevisiae*	
ath-miR5021	Supercontig_2.6	100	16	0	0	0.036	32.2	*S. cerevisiae*	
ath-miR5021	Supercontig_2.6	100	16	0	0	0.036	32.2	*S. cerevisiae*	
ath-miR5021	Supercontig_2.6	100	16	0	0	0.036	32.2	*S. cerevisiae*	
ath-miR5021	Supercontig_2.6	100	16	0	0	0.036	32.2	*S. cerevisiae*	
ath-miR5021	Supercontig_2.6	100	16	0	0	0.036	32.2	*S. cerevisiae*	
ath-miR5021	Supercontig_2.6	100	16	0	0	0.036	32.2	*S. cerevisiae*	
ath-miR5021	Supercontig_2.6	100	16	0	0	0.036	32.2	*S. cerevisiae*	
ath-miR5021	Supercontig_2.6	100	16	0	0	0.036	32.2	*S. cerevisiae*	
ath-miR5021	Supercontig_2.6	100	16	0	0	0.036	32.2	*S. cerevisiae*	
ath-miR5021	Supercontig_2.6	100	16	0	0	0.036	32.2	*S. cerevisiae*	
ath-miR5021	Supercontig_2.6	100	16	0	0	0.036	32.2	*S. cerevisiae*	
ath-miR5021	Supercontig_2.6	100	16	0	0	0.036	32.2	*S. cerevisiae*	
ath-miR5021	Supercontig_2.6	100	16	0	0	0.036	32.2	*S. cerevisiae*	
ath-miR5021	Supercontig_2.6	100	16	0	0	0.036	32.2	*S. cerevisiae*	
ath-miR5021	Supercontig_2.6	100	16	0	0	0.036	32.2	*S. cerevisiae*	
ath-miR5021	Supercontig_2.6	100	16	0	0	0.036	32.2	*S. cerevisiae*	
ath-miR5021	Supercontig_2.6	100	16	0	0	0.036	32.2	*S. cerevisiae*	
ath-miR5021	Supercontig_2.6	100	16	0	0	0.036	32.2	*S. cerevisiae*	
ath-miR5021	Supercontig_2.6	100	16	0	0	0.036	32.2	*S. cerevisiae*	
ath-miR5021	Supercontig_2.6	100	16	0	0	0.036	32.2	*S. cerevisiae*	
ath-miR5021	Supercontig_2.6	100	16	0	0	0.036	32.2	*S. cerevisiae*	
ath-miR5021	Supercontig_2.6	100	16	0	0	0.036	32.2	*S. cerevisiae*	
ath-miR5021	Supercontig_2.6	100	16	0	0	0.036	32.2	*S. cerevisiae*	
ath-miR5021	Supercontig_2.6	100	16	0	0	0.036	32.2	*S. cerevisiae*	
ath-miR5021	Supercontig_2.6	100	16	0	0	0.036	32.2	*S. cerevisiae*	
ath-miR5021	Supercontig_2.6	100	16	0	0	0.036	32.2	*S. cerevisiae*	

Query id[a]	Subject id[b]	% Identity[c]	Alignment length[d]	Mismatches[e]	Gap opens[f]	Evalue [g]	Bit score[h]	Fungi vesicle[i]	Reference[j]
ath-miR5021	Supercontig_2.6	100	16	0	0	0.036	32.2	*S. cerevisiae*	
ath-miR5021	Supercontig_2.6	100	16	0	0	0.036	32.2	*S. cerevisiae*	
ath-miR5021	Supercontig_2.6	100	16	0	0	0.036	32.2	*S. cerevisiae*	
ath-miR5021	Supercontig_2.6	100	16	0	0	0.036	32.2	*S. cerevisiae*	
ath-miR5021	Supercontig_2.6	100	16	0	0	0.036	32.2	*S. cerevisiae*	
ath-miR5021	Supercontig_2.6	100	16	0	0	0.036	32.2	*S. cerevisiae*	
ath-miR5021	Supercontig_2.6	100	16	0	0	0.036	32.2	*S. cerevisiae*	
ath-miR5021	Supercontig_2.6	100	16	0	0	0.036	32.2	*S. cerevisiae*	
ath-miR5021	Supercontig_2.6	100	16	0	0	0.036	32.2	*S. cerevisiae*	
ath-miR5021	Supercontig_2.6	100	16	0	0	0.036	32.2	*S. cerevisiae*	
ath-miR5021	Supercontig_2.6	100	16	0	0	0.036	32.2	*S. cerevisiae*	
ath-miR5021	Supercontig_2.6	100	16	0	0	0.036	32.2	*S. cerevisiae*	
ath-miR5021	Supercontig_2.6	100	16	0	0	0.036	32.2	*S. cerevisiae*	
ath-miR5021	Supercontig_2.6	100	16	0	0	0.036	32.2	*S. cerevisiae*	
ath-miR5021	Supercontig_2.6	100	16	0	0	0.036	32.2	*S. cerevisiae*	
ath-miR5021	Supercontig_2.6	100	16	0	0	0.036	32.2	*S. cerevisiae*	
ath-miR5021	Supercontig_2.6	100	16	0	0	0.036	32.2	*S. cerevisiae*	
ath-miR5021	Supercontig_2.6	100	16	0	0	0.036	32.2	*S. cerevisiae*	
ath-miR5021	Supercontig_2.6	100	16	0	0	0.036	32.2	*S. cerevisiae*	
ath-miR5021	Supercontig_2.6	100	16	0	0	0.036	32.2	*S. cerevisiae*	
ath-miR5021	Supercontig_2.6	100	16	0	0	0.036	32.2	*S. cerevisiae*	
ath-miR5021	Supercontig_2.6	100	16	0	0	0.036	32.2	*S. cerevisiae*	
ath-miR5021	Supercontig_2.6	100	16	0	0	0.036	32.2	*S. cerevisiae*	
ath-miR5021	Supercontig_2.6	100	16	0	0	0.036	32.2	*S. cerevisiae*	
ath-miR5021	Supercontig_2.6	100	16	0	0	0.036	32.2	*S. cerevisiae*	
ath-miR5021	Supercontig_2.6	100	16	0	0	0.036	32.2	*S. cerevisiae*	
ath-miR5021	Supercontig_2.6	100	16	0	0	0.036	32.2	*S. cerevisiae*	
ath-miR5021	Supercontig_2.6	100	16	0	0	0.036	32.2	*S. cerevisiae*	
ath-miR5021	Supercontig_2.6	100	16	0	0	0.036	32.2	*S. cerevisiae*	
ath-miR5021	Supercontig_2.6	100	16	0	0	0.036	32.2	*S. cerevisiae*	
ath-miR5021	Supercontig_2.6	100	16	0	0	0.036	32.2	*S. cerevisiae*	
ath-miR5021	Supercontig_2.6	100	16	0	0	0.036	32.2	*S. cerevisiae*	
ath-miR5021	Supercontig_2.6	100	16	0	0	0.036	32.2	*S. cerevisiae*	
ath-miR5021	Supercontig_2.6	100	16	0	0	0.036	32.2	*S. cerevisiae*	
ath-miR5021	Supercontig_2.6	100	16	0	0	0.036	32.2	*S. cerevisiae*	
ath-miR5021	Supercontig_2.6	100	16	0	0	0.036	32.2	*S. cerevisiae*	
ath-miR5021	Supercontig_2.6	100	16	0	0	0.036	32.2	*S. cerevisiae*	
ath-miR5021	Supercontig_2.6	100	17	0	0	0.009	34.2	*S. cerevisiae*	
ath-miR5021	Supercontig_2.6	100	17	0	0	0.009	34.2	*S. cerevisiae*	
ath-miR5021	Supercontig_2.6	100	17	0	0	0.009	34.2	*S. cerevisiae*	
ath-miR5021	Supercontig_2.6	100	17	0	0	0.009	34.2	*S. cerevisiae*	
ath-miR5021	Supercontig_2.6	100	17	0	0	0.009	34.2	*S. cerevisiae*	
ath-miR5021	Supercontig_2.6	100	17	0	0	0.009	34.2	*S. cerevisiae*	
ath-miR5021	Supercontig_2.6	100	17	0	0	0.009	34.2	*S. cerevisiae*	
ath-miR5021	Supercontig_2.6	100	18	0	0	0.002	36.2	*S. cerevisiae*	
ath-miR5021	Supercontig_2.6	100	18	0	0	0.002	36.2	*S. cerevisiae*	

Query id[a]	Subject id[b]	% Identity[c]	Alignment length[d]	Mismatches[e]	Gap opens[f]	Evalue [g]	Bit score[h]	Fungi vesicle[i]	Reference[j]
ath-miR5021	Supercontig_2.6	100	18	0	0	0.002	36.2	*S. cerevisiae*	
ath-miR5021	Supercontig_2.6	100	18	0	0	0.002	36.2	*S. cerevisiae*	
ath-miR5021	Supercontig_2.6	100	18	0	0	0.002	36.2	*S. cerevisiae*	
ath-miR5021	Supercontig_2.6	100	18	0	0	0.002	36.2	*S. cerevisiae*	
ath-miR5021	Supercontig_2.7	100	16	0	0	0.036	32.2	*S. cerevisiae*	
ath-miR5021	Supercontig_2.7	100	16	0	0	0.036	32.2	*S. cerevisiae*	
ath-miR5021	Supercontig_2.7	100	16	0	0	0.036	32.2	*S. cerevisiae*	
ath-miR5021	Supercontig_2.7	100	16	0	0	0.036	32.2	*S. cerevisiae*	
ath-miR5021	Supercontig_2.7	100	16	0	0	0.036	32.2	*S. cerevisiae*	
ath-miR5021	Supercontig_2.7	100	16	0	0	0.036	32.2	*S. cerevisiae*	
ath-miR5021	Supercontig_2.7	100	16	0	0	0.036	32.2	*S. cerevisiae*	
ath-miR5021	Supercontig_2.7	100	16	0	0	0.036	32.2	*S. cerevisiae*	
ath-miR5021	Supercontig_2.7	100	16	0	0	0.036	32.2	*S. cerevisiae*	
ath-miR5021	Supercontig_2.7	100	16	0	0	0.036	32.2	*S. cerevisiae*	
ath-miR5021	Supercontig_2.7	100	16	0	0	0.036	32.2	*S. cerevisiae*	
ath-miR5021	Supercontig_2.7	100	16	0	0	0.036	32.2	*S. cerevisiae*	
ath-miR5021	Supercontig_2.7	100	16	0	0	0.036	32.2	*S. cerevisiae*	
ath-miR5021	Supercontig_2.7	100	16	0	0	0.036	32.2	*S. cerevisiae*	
ath-miR5021	Supercontig_2.7	100	16	0	0	0.036	32.2	*S. cerevisiae*	
ath-miR5021	Supercontig_2.7	100	16	0	0	0.036	32.2	*S. cerevisiae*	
ath-miR5021	Supercontig_2.7	100	16	0	0	0.036	32.2	*S. cerevisiae*	
ath-miR5021	Supercontig_2.7	100	16	0	0	0.036	32.2	*S. cerevisiae*	
ath-miR5021	Supercontig_2.7	100	16	0	0	0.036	32.2	*S. cerevisiae*	
ath-miR5021	Supercontig_2.7	100	16	0	0	0.036	32.2	*S. cerevisiae*	
ath-miR5021	Supercontig_2.7	100	16	0	0	0.036	32.2	*S. cerevisiae*	
ath-miR5021	Supercontig_2.7	100	16	0	0	0.036	32.2	*S. cerevisiae*	
ath-miR5021	Supercontig_2.7	100	16	0	0	0.036	32.2	*S. cerevisiae*	
ath-miR5021	Supercontig_2.7	100	16	0	0	0.036	32.2	*S. cerevisiae*	
ath-miR5021	Supercontig_2.7	100	16	0	0	0.036	32.2	*S. cerevisiae*	
ath-miR5021	Supercontig_2.7	100	16	0	0	0.036	32.2	*S. cerevisiae*	
ath-miR5021	Supercontig_2.7	100	16	0	0	0.036	32.2	*S. cerevisiae*	
ath-miR5021	Supercontig_2.7	100	16	0	0	0.036	32.2	*S. cerevisiae*	
ath-miR5021	Supercontig_2.7	100	16	0	0	0.036	32.2	*S. cerevisiae*	
ath-miR5021	Supercontig_2.7	100	16	0	0	0.036	32.2	*S. cerevisiae*	
ath-miR5021	Supercontig_2.7	100	16	0	0	0.036	32.2	*S. cerevisiae*	
ath-miR5021	Supercontig_2.7	100	16	0	0	0.036	32.2	*S. cerevisiae*	
ath-miR5021	Supercontig_2.7	100	16	0	0	0.036	32.2	*S. cerevisiae*	
ath-miR5021	Supercontig_2.7	100	16	0	0	0.036	32.2	*S. cerevisiae*	
ath-miR5021	Supercontig_2.7	100	16	0	0	0.036	32.2	*S. cerevisiae*	
ath-miR5021	Supercontig_2.7	100	16	0	0	0.036	32.2	*S. cerevisiae*	
ath-miR5021	Supercontig_2.7	100	16	0	0	0.036	32.2	*S. cerevisiae*	
ath-miR5021	Supercontig_2.7	100	16	0	0	0.036	32.2	*S. cerevisiae*	
ath-miR5021	Supercontig_2.7	100	16	0	0	0.036	32.2	*S. cerevisiae*	
ath-miR5021	Supercontig_2.7	100	16	0	0	0.036	32.2	*S. cerevisiae*	
ath-miR5021	Supercontig_2.7	100	16	0	0	0.036	32.2	*S. cerevisiae*	
ath-miR5021	Supercontig_2.7	100	16	0	0	0.036	32.2	*S. cerevisiae*	

Query id[a]	Subject id[b]	% Identity[c]	Alignment length[d]	Mismatches[e]	Gap opens[f]	Evalue [g]	Bit score[h]	Fungi vesicle[i]	Reference[j]
ath-miR5021	Supercontig_2.7	100	16	0	0	0.036	32.2	*S. cerevisiae*	
ath-miR5021	Supercontig_2.7	100	16	0	0	0.036	32.2	*S. cerevisiae*	
ath-miR5021	Supercontig_2.7	100	16	0	0	0.036	32.2	*S. cerevisiae*	
ath-miR5021	Supercontig_2.7	100	16	0	0	0.036	32.2	*S. cerevisiae*	
ath-miR5021	Supercontig_2.7	100	16	0	0	0.036	32.2	*S. cerevisiae*	
ath-miR5021	Supercontig_2.7	100	16	0	0	0.036	32.2	*S. cerevisiae*	
ath-miR5021	Supercontig_2.7	100	16	0	0	0.036	32.2	*S. cerevisiae*	
ath-miR5021	Supercontig_2.7	100	16	0	0	0.036	32.2	*S. cerevisiae*	
ath-miR5021	Supercontig_2.7	100	16	0	0	0.036	32.2	*S. cerevisiae*	
ath-miR5021	Supercontig_2.7	100	16	0	0	0.036	32.2	*S. cerevisiae*	
ath-miR5021	Supercontig_2.7	100	16	0	0	0.036	32.2	*S. cerevisiae*	
ath-miR5021	Supercontig_2.7	100	16	0	0	0.036	32.2	*S. cerevisiae*	
ath-miR5021	Supercontig_2.7	100	16	0	0	0.036	32.2	*S. cerevisiae*	
ath-miR5021	Supercontig_2.7	100	16	0	0	0.036	32.2	*S. cerevisiae*	
ath-miR5021	Supercontig_2.7	100	16	0	0	0.036	32.2	*S. cerevisiae*	
ath-miR5021	Supercontig_2.7	100	16	0	0	0.036	32.2	*S. cerevisiae*	
ath-miR5021	Supercontig_2.7	100	16	0	0	0.036	32.2	*S. cerevisiae*	
ath-miR5021	Supercontig_2.7	100	16	0	0	0.036	32.2	*S. cerevisiae*	
ath-miR5021	Supercontig_2.7	100	16	0	0	0.036	32.2	*S. cerevisiae*	
ath-miR5021	Supercontig_2.7	100	16	0	0	0.036	32.2	*S. cerevisiae*	
ath-miR5021	Supercontig_2.7	100	16	0	0	0.036	32.2	*S. cerevisiae*	
ath-miR5021	Supercontig_2.7	100	16	0	0	0.036	32.2	*S. cerevisiae*	
ath-miR5021	Supercontig_2.7	100	16	0	0	0.036	32.2	*S. cerevisiae*	
ath-miR5021	Supercontig_2.7	100	16	0	0	0.036	32.2	*S. cerevisiae*	
ath-miR5021	Supercontig_2.7	100	16	0	0	0.036	32.2	*S. cerevisiae*	
ath-miR5021	Supercontig_2.7	100	16	0	0	0.036	32.2	*S. cerevisiae*	
ath-miR5021	Supercontig_2.7	100	16	0	0	0.036	32.2	*S. cerevisiae*	
ath-miR5021	Supercontig_2.7	100	16	0	0	0.036	32.2	*S. cerevisiae*	
ath-miR5021	Supercontig_2.7	100	16	0	0	0.036	32.2	*S. cerevisiae*	
ath-miR5021	Supercontig_2.7	100	16	0	0	0.036	32.2	*S. cerevisiae*	
ath-miR5021	Supercontig_2.7	100	16	0	0	0.036	32.2	*S. cerevisiae*	
ath-miR5021	Supercontig_2.7	100	16	0	0	0.036	32.2	*S. cerevisiae*	
ath-miR5023	Supercontig_2.7	100	16	0	0	0.042	32.2	*C. albicans/ C.neoformans*	
ath-miR864-3p*	Supercontig_2.5	100	16	0	0	0.048	32.2	*C.neoformans*	
bdi-miR399b	Supercontig_2.3	100	16	0	0	0.042	32.2	*C. albicans*	
bdi-miR529-3p*	Supercontig_2.1	100	16	0	0	0.042	32.2	*C.neoformans*	
bdi-miR529-3p*	Supercontig_2.5	100	16	0	0	0.042	32.2	*C.neoformans*	
bmo-miR-2768-5p	Supercontig_2.3	100	17	0	0	0.012	34.2	*C. albicans*	
bmo-miR-3250	Supercontig_2.3	100	17	0	0	0.011	34.2	*C. albicans/ P. brasiliensis*	
bmo-miR-3250	Supercontig_2.3	100	18	0	0	0.003	36.2	*C. albicans/ P. brasiliensis*	
bmo-miR-3250	Supercontig_2.6	100	16	0	0	0.042	32.2	*C. albicans/ P. brasiliensis*	
bmo-miR-3306	Supercontig_2.7	100	16	0	0	0.06	32.2	*C.neoformans*	
bmo-miR-3382-3p*	Supercontig_2.1	100	16	0	0	0.066	32.2	*P. brasiliensis*	
bmo-miR-3425	Supercontig_2.2	100	16	0	0	0.066	32.2	*C. albicans*	
bmo-miR-3430	Supercontig_2.1	95.24	21	1	0	0.017	34.2	*C. albicans*	
bta-miR-1284	Supercontig_2.3	100	16	0	0	0.048	32.2	*C. albicans*	

Query id[a]	Subject id[b]	% Identity[c]	Alignment length[d]	Mismatches[e]	Gap opens[f]	Evalue [g]	Bit score[h]	Fungi vesicle[i]	Reference[j]
bta-miR-2304	Supercontig_2.10	100	16	0	0	0.036	32.2	*C.neoformans*	
bta-miR-2304	Supercontig_2.4	100	16	0	0	0.036	32.2	*C.neoformans*	
bta-miR-2437	Supercontig_2.8	100	16	0	0	0.042	32.2	*C. albicans*	
cfa-miR-214	Supercontig_2.6	100	17	0	0	0.012	34.2	*C.neoformans*	
cin-miR-367-5p	Supercontig_2.2	100	17	0	0	0.009	34.2	*C.neoformans/C. albicans*	
cin-miR-4171-5p	Supercontig_2.11	100	16	0	0	0.036	32.2	*C.neoformans/C. albicans/S. cerevisiae*	
cin-miR-4171-5p	Supercontig_2.32	95	20	1	0	0.036	32.2	*C.neoformans/C. albicans/S. cerevisiae*	
cin-miR-4171-5p	Supercontig_2.32	95	20	1	0	0.036	32.2	*C.neoformans/C. albicans/S. cerevisiae*	
cin-miR-4171-5p	Supercontig_2.32	95	20	1	0	0.036	32.2	*C.neoformans/C. albicans/S. cerevisiae*	
cin-miR-4171-5p	Supercontig_2.32	95	20	1	0	0.036	32.2	*C.neoformans/C. albicans/S. cerevisiae*	
cin-miR-4171-5p	Supercontig_2.32	95	20	1	0	0.036	32.2	*C.neoformans/C. albicans/S. cerevisiae*	
cin-miR-4171-5p	Supercontig_2.32	95	20	1	0	0.036	32.2	*C.neoformans/C. albicans/S. cerevisiae*	
cin-miR-4171-5p	Supercontig_2.32	95	20	1	0	0.036	32.2	*C.neoformans/C. albicans/S. cerevisiae*	
cin-miR-4171-5p	Supercontig_2.8	95	20	1	0	0.036	32.2	*C.neoformans/C. albicans/S. cerevisiae*	
cin-miR-4171-5p	Supercontig_2.8	95	20	1	0	0.036	32.2	*C.neoformans/C. albicans/S. cerevisiae*	
cin-miR-4171-5p	Supercontig_2.8	95	20	1	0	0.036	32.2	*C.neoformans/C. albicans/S. cerevisiae*	
csi-miR3946	Supercontig_2.13	100	16	0	0	0.06	32.2	*C.neoformans/S. cerevisiae*	
csi-miR3946	Supercontig_2.2	100	16	0	0	0.06	32.2	*C.neoformans/S. cerevisiae*	
csi-miR3946	Supercontig_2.3	100	16	0	0	0.06	32.2	*C.neoformans/S. cerevisiae*	
csi-miR3946	Supercontig_2.6	100	16	0	0	0.06	32.2	*C.neoformans/S. cerevisiae*	
csi-miR3946	Supercontig_2.6	100	16	0	0	0.06	32.2	*C.neoformans/S. cerevisiae*	
csi-miR3946	Supercontig_2.8	100	16	0	0	0.06	32.2	*C.neoformans/S. cerevisiae*	
csi-miR3946	Supercontig_2.8	100	17	0	0	0.015	34.2	*C.neoformans/S. cerevisiae*	
der-miR-13b	Supercontig_2.11	100	16	0	0	0.048	32.2	*P.brasiliensis/C.neoformans/S. cerevisiae*	
dme-miR-2491-3p*	Supercontig_2.10	100	17	0	0	0.008	34.2	*S. cerevisiae*	
dme-miR-2491-3p*	Supercontig_2.10	100	17	0	0	0.008	34.2	*S. cerevisiae*	
dme-miR-2491-3p*	Supercontig_2.10	100	18	0	0	0.002	36.2	*S. cerevisiae*	
dme-miR-2491-3p*	Supercontig_2.1	100	16	0	0	0.03	32.2	*S. cerevisiae*	
dme-miR-2491-3p*	Supercontig_2.1	100	17	0	0	0.008	34.2	*S. cerevisiae*	
dme-miR-2491-3p*	Supercontig_2.1	100	17	0	0	0.008	34.2	*S. cerevisiae*	
dme-miR-2491-3p*	Supercontig_2.1	100	18	0	0	0.002	36.2	*S. cerevisiae*	
dme-miR-2491-3p*	Supercontig_2.1	100	18	0	0	0.002	36.2	*S. cerevisiae*	
dme-miR-2491-3p*	Supercontig_2.11	100	17	0	0	0.008	34.2	*S. cerevisiae*	
dme-miR-2491-3p*	Supercontig_2.12	100	17	0	0	0.008	34.2	*S. cerevisiae*	
dme-miR-4968-3p*	Supercontig_2.10	100	16	0	0	0.048	32.2	*C.neoformans/S. cerevisiae*	
dme-miR-4968-3p*	Supercontig_2.10	100	16	0	0	0.048	32.2	*C.neoformans/S. cerevisiae*	
dme-miR-4968-3p*	Supercontig_2.10	100	17	0	0	0.012	34.2	*C.neoformans/S. cerevisiae*	
dme-miR-4968-3p*	Supercontig_2.10	100	17	0	0	0.012	34.2	*C.neoformans/S. cerevisiae*	
dme-miR-4968-3p*	Supercontig_2.10	100	17	0	0	0.012	34.2	*C.neoformans/S. cerevisiae*	
dme-miR-4968-3p*	Supercontig_2.10	100	18	0	0	0.003	36.2	*C.neoformans/S. cerevisiae*	
dme-miR-4968-3p*	Supercontig_2.10	100	21	0	0	5.00E-05	42.1	*C.neoformans/S. cerevisiae*	
dme-miR-4968-3p*	Supercontig_2.10	100	21	0	0	5.00E-05	42.1	*C.neoformans/S. cerevisiae*	
dme-miR-4968-3p*	Supercontig_2.10	95	20	1	0	0.048	32.2	*C.neoformans/S. cerevisiae*	
dme-miR-4968-3p*	Supercontig_2.10	95	20	1	0	0.048	32.2	*C.neoformans/S. cerevisiae*	
dme-miR-4968-3p*	Supercontig_2.10	95	20	1	0	0.048	32.2	*C.neoformans/S. cerevisiae*	
dme-miR-4968-3p*	Supercontig_2.10	95	20	1	0	0.048	32.2	*C.neoformans/S. cerevisiae*	

Query id[a]	Subject id[b]	% Identity[c]	Alignment length[d]	Mismatches[e]	Gap opens[f]	Evalue [g]	Bit score[h]	Fungi vesicle[i]	Reference[j]
dme-miR-4968-3p*	Supercontig_2.10	95	20	1	0	0.048	32.2	*C.neoformans/S. cerevisiae*	
dme-miR-4968-3p*	Supercontig_2.10	95	20	1	0	0.048	32.2	*C.neoformans/S. cerevisiae*	
dme-miR-4968-3p*	Supercontig_2.10	95.24	21	1	0	0.012	34.2	*C.neoformans/S. cerevisiae*	
dme-miR-4968-3p*	Supercontig_2.10	95.24	21	1	0	0.012	34.2	*C.neoformans/S. cerevisiae*	
dme-miR-4968-3p*	Supercontig_2.10	95.24	21	1	0	0.012	34.2	*C.neoformans/S. cerevisiae*	
dme-miR-4968-3p*	Supercontig_2.10	95.24	21	1	0	0.012	34.2	*C.neoformans/S. cerevisiae*	
dme-miR-4968-3p*	Supercontig_2.10	95.24	21	1	0	0.012	34.2	*C.neoformans/S. cerevisiae*	
dme-miR-4968-3p*	Supercontig_2.10	95.24	21	1	0	0.012	34.2	*C.neoformans/S. cerevisiae*	
dme-miR-4968-3p*	Supercontig_2.10	95.24	21	1	0	0.012	34.2	*C.neoformans/S. cerevisiae*	
dme-miR-4968-3p*	Supercontig_2.10	95.24	21	1	0	0.012	34.2	*C.neoformans/S. cerevisiae*	
dme-miR-4968-3p*	Supercontig_2.10	95.24	21	1	0	0.012	34.2	*C.neoformans/S. cerevisiae*	
dme-miR-4968-3p*	Supercontig_2.10	95.24	21	1	0	0.012	34.2	*C.neoformans/S. cerevisiae*	
dme-miR-4968-3p*	Supercontig_2.10	95.24	21	1	0	0.012	34.2	*C.neoformans/S. cerevisiae*	
dme-miR-4968-3p*	Supercontig_2.10	95.24	21	1	0	0.012	34.2	*C.neoformans/S. cerevisiae*	
dme-miR-4968-3p*	Supercontig_2.10	95.24	21	1	0	0.012	34.2	*C.neoformans/S. cerevisiae*	
dme-miR-4968-3p*	Supercontig_2.10	95.24	21	1	0	0.012	34.2	*C.neoformans/S. cerevisiae*	
dme-miR-4968-3p*	Supercontig_2.10	95.24	21	1	0	0.012	34.2	*C.neoformans/S. cerevisiae*	
dme-miR-4968-3p*	Supercontig_2.1	100	16	0	0	0.048	32.2	*C.neoformans/S. cerevisiae*	
dme-miR-4968-3p*	Supercontig_2.1	100	16	0	0	0.048	32.2	*C.neoformans/S. cerevisiae*	
dme-miR-4968-3p*	Supercontig_2.1	100	16	0	0	0.048	32.2	*C.neoformans/S. cerevisiae*	
dme-miR-4968-3p*	Supercontig_2.1	100	16	0	0	0.048	32.2	*C.neoformans/S. cerevisiae*	
dme-miR-4968-3p*	Supercontig_2.1	100	17	0	0	0.012	34.2	*C.neoformans/S. cerevisiae*	
dme-miR-4968-3p*	Supercontig_2.1	100	17	0	0	0.012	34.2	*C.neoformans/S. cerevisiae*	
dme-miR-4968-3p*	Supercontig_2.1	100	17	0	0	0.012	34.2	*C.neoformans/S. cerevisiae*	
dme-miR-4968-3p*	Supercontig_2.1	100	17	0	0	0.012	34.2	*C.neoformans/S. cerevisiae*	
dme-miR-4968-3p*	Supercontig_2.1	100	18	0	0	0.003	36.2	*C.neoformans/S. cerevisiae*	
dme-miR-4968-3p*	Supercontig_2.1	100	18	0	0	0.003	36.2	*C.neoformans/S. cerevisiae*	
dme-miR-4968-3p*	Supercontig_2.1	100	18	0	0	0.003	36.2	*C.neoformans/S. cerevisiae*	
dme-miR-4968-3p*	Supercontig_2.11	100	16	0	0	0.048	32.2	*C.neoformans/S. cerevisiae*	
dme-miR-4968-3p*	Supercontig_2.11	100	17	0	0	0.012	34.2	*C.neoformans/S. cerevisiae*	
dme-miR-4968-3p*	Supercontig_2.11	100	18	0	0	0.003	36.2	*C.neoformans/S. cerevisiae*	
dme-miR-4968-3p*	Supercontig_2.11	95	20	1	0	0.048	32.2	*C.neoformans/S. cerevisiae*	
dme-miR-4968-3p*	Supercontig_2.11	95	20	1	0	0.048	32.2	*C.neoformans/S. cerevisiae*	
dme-miR-4968-3p*	Supercontig_2.11	95	20	1	0	0.048	32.2	*C.neoformans/S. cerevisiae*	
dme-miR-4968-3p*	Supercontig_2.12	100	16	0	0	0.048	32.2	*C.neoformans/S. cerevisiae*	
dme-miR-4968-3p*	Supercontig_2.12	100	17	0	0	0.012	34.2	*C.neoformans/S. cerevisiae*	
dme-miR-4968-3p*	Supercontig_2.12	100	17	0	0	0.012	34.2	*C.neoformans/S. cerevisiae*	
dme-miR-4968-3p*	Supercontig_2.12	100	17	0	0	0.012	34.2	*C.neoformans/S. cerevisiae*	
dme-miR-4968-3p*	Supercontig_2.12	100	17	0	0	0.012	34.2	*C.neoformans/S. cerevisiae*	
dme-miR-4968-3p*	Supercontig_2.12	95	20	1	0	0.048	32.2	*C.neoformans/S. cerevisiae*	
dme-miR-4968-3p*	Supercontig_2.12	95	20	1	0	0.048	32.2	*C.neoformans/S. cerevisiae*	
dme-miR-4968-3p*	Supercontig_2.12	95	20	1	0	0.048	32.2	*C.neoformans/S. cerevisiae*	
dme-miR-4968-3p*	Supercontig_2.12	95	20	1	0	0.048	32.2	*C.neoformans/S. cerevisiae*	
dme-miR-4968-3p*	Supercontig_2.12	95.24	21	1	0	0.012	34.2	*C.neoformans/S. cerevisiae*	
dme-miR-4968-3p*	Supercontig_2.12	95.24	21	1	0	0.012	34.2	*C.neoformans/S. cerevisiae*	
dme-miR-4968-3p*	Supercontig_2.12	95.24	21	1	0	0.012	34.2	*C.neoformans/S. cerevisiae*	

Query id[a]	Subject id[b]	% Identity[c]	Alignment length[d]	Mismatches[e]	Gap opens[f]	Evalue [g]	Bit score[h]	Fungi vesicle[i]	Reference[j]
dme-miR-4968-3p*	Supercontig_2.12	95.24	21	1	0	0.012	34.2	*C.neoformans/S. cerevisiae*	
dme-miR-4968-3p*	Supercontig_2.12	95.24	21	1	0	0.012	34.2	*C.neoformans/S. cerevisiae*	
dme-miR-4968-3p*	Supercontig_2.12	95.24	21	1	0	0.012	34.2	*C.neoformans/S. cerevisiae*	
dme-miR-4968-3p*	Supercontig_2.12	95.24	21	1	0	0.012	34.2	*C.neoformans/S. cerevisiae*	
dme-miR-4968-3p*	Supercontig_2.12	95.24	21	1	0	0.012	34.2	*C.neoformans/S. cerevisiae*	
dme-miR-4968-3p*	Supercontig_2.12	95.24	21	1	0	0.012	34.2	*C.neoformans/S. cerevisiae*	
dme-miR-4968-3p*	Supercontig_2.12	95.24	21	1	0	0.012	34.2	*C.neoformans/S. cerevisiae*	
dme-miR-4968-3p*	Supercontig_2.12	95.24	21	1	0	0.012	34.2	*C.neoformans/S. cerevisiae*	
dme-miR-4968-3p*	Supercontig_2.12	95.24	21	1	0	0.012	34.2	*C.neoformans/S. cerevisiae*	
dme-miR-4968-3p*	Supercontig_2.12	95.24	21	1	0	0.012	34.2	*C.neoformans/S. cerevisiae*	
dme-miR-4968-3p*	Supercontig_2.12	95.24	21	1	0	0.012	34.2	*C.neoformans/S. cerevisiae*	
dme-miR-4968-3p*	Supercontig_2.12	95.24	21	1	0	0.012	34.2	*C.neoformans/S. cerevisiae*	
dme-miR-4968-3p*	Supercontig_2.12	95.24	21	1	0	0.012	34.2	*C.neoformans/S. cerevisiae*	
dme-miR-4968-3p*	Supercontig_2.12	95.24	21	1	0	0.012	34.2	*C.neoformans/S. cerevisiae*	
dme-miR-4968-3p*	Supercontig_2.12	95.24	21	1	0	1.20E-02	34.2	*C.neoformans/S. cerevisiae*	
dme-miR-4968-3p*	Supercontig_2.12	95.24	21	1	0	1.20E-02	34.2	*C.neoformans/S. cerevisiae*	
dme-miR-4968-3p*	Supercontig_2.12	95.24	21	1	0	1.20E-02	34.2	*C.neoformans/S. cerevisiae*	
dme-miR-4968-3p*	Supercontig_2.12	95.24	21	1	0	1.20E-02	34.2	*C.neoformans/S. cerevisiae*	
dme-miR-4968-3p*	Supercontig_2.12	95.24	21	1	0	1.20E-02	34.2	*C.neoformans/S. cerevisiae*	
dme-miR-4968-3p*	Supercontig_2.12	95.24	21	1	0	1.20E-02	34.2	*C.neoformans/S. cerevisiae*	
dme-miR-4968-3p*	Supercontig_2.13	100	16	0	0	4.80E-02	32.2	*C.neoformans/S. cerevisiae*	
dme-miR-4968-3p*	Supercontig_2.13	100	16	0	0	4.80E-02	32.2	*C.neoformans/S. cerevisiae*	
dme-miR-4968-3p*	Supercontig_2.13	100	16	0	0	0.048	32.2	*C.neoformans/S. cerevisiae*	
dme-miR-4968-3p*	Supercontig_2.13	100	16	0	0	0.048	32.2	*C.neoformans/S. cerevisiae*	
dme-miR-4968-3p*	Supercontig_2.13	100	16	0	0	0.048	32.2	*C.neoformans/S. cerevisiae*	
dme-miR-4968-3p*	Supercontig_2.13	100	17	0	0	0.012	34.2	*C.neoformans/S. cerevisiae*	
dme-miR-4968-3p*	Supercontig_2.13	100	18	0	0	0.003	36.2	*C.neoformans/S. cerevisiae*	
dme-miR-4968-3p*	Supercontig_2.13	100	18	0	0	0.003	36.2	*C.neoformans/S. cerevisiae*	
dme-miR-4968-3p*	Supercontig_2.13	100	19	0	0	8.00E-04	38.2	*C.neoformans/S. cerevisiae*	
dme-miR-4968-3p*	Supercontig_2.13	95	20	1	0	0.048	32.2	*C.neoformans/S. cerevisiae*	
dme-miR-4968-3p*	Supercontig_2.13	95	20	1	0	0.048	32.2	*C.neoformans/S. cerevisiae*	
dme-miR-4968-3p*	Supercontig_2.13	95	20	1	0	0.048	32.2	*C.neoformans/S. cerevisiae*	
dme-miR-4968-3p*	Supercontig_2.13	95	20	1	0	0.048	32.2	*C.neoformans/S. cerevisiae*	
dme-miR-4968-3p*	Supercontig_2.13	95	20	1	0	0.048	32.2	*C.neoformans/S. cerevisiae*	
dme-miR-4968-3p*	Supercontig_2.13	95.24	21	1	0	0.012	34.2	*C.neoformans/S. cerevisiae*	
dme-miR-4968-3p*	Supercontig_2.13	95.24	21	1	0	0.012	34.2	*C.neoformans/S. cerevisiae*	
dme-miR-4968-3p*	Supercontig_2.13	95.24	21	1	0	0.012	34.2	*C.neoformans/S. cerevisiae*	
dme-miR-4968-3p*	Supercontig_2.13	95.24	21	1	0	0.012	34.2	*C.neoformans/S. cerevisiae*	
dme-miR-4968-3p*	Supercontig_2.13	95.24	21	1	0	0.012	34.2	*C.neoformans/S. cerevisiae*	
dme-miR-4968-3p*	Supercontig_2.13	95.24	21	1	0	0.012	34.2	*C.neoformans/S. cerevisiae*	
dme-miR-4968-3p*	Supercontig_2.13	95.24	21	1	0	0.012	34.2	*C.neoformans/S. cerevisiae*	
dme-miR-4968-3p*	Supercontig_2.13	95.24	21	1	0	0.012	34.2	*C.neoformans/S. cerevisiae*	
dme-miR-4968-3p*	Supercontig_2.13	95.24	21	1	0	0.012	34.2	*C.neoformans/S. cerevisiae*	
dme-miR-4968-3p*	Supercontig_2.14	100	17	0	0	0.012	34.2	*C.neoformans/S. cerevisiae*	
dme-miR-4968-3p*	Supercontig_2.14	95	20	1	0	0.048	32.2	*C.neoformans/S. cerevisiae*	
dme-miR-4968-3p*	Supercontig_2.15	100	16	0	0	0.048	32.2	*C.neoformans/S. cerevisiae*	

Query id[a]	Subject id[b]	% Identity[c]	Alignment length[d]	Mismatches[e]	Gap opens[f]	Evalue [g]	Bit score[h]	Fungi vesicle[i]	Reference[j]
dme-miR-4968-3p*	Supercontig_2.17	100	17	0	0	0.012	34.2	*C.neoformans/S. cerevisiae*	
dme-miR-4968-3p*	Supercontig_2.17	100	17	0	0	0.012	34.2	*C.neoformans/S. cerevisiae*	
dme-miR-4968-3p*	Supercontig_2.17	100	18	0	0	0.003	36.2	*C.neoformans/S. cerevisiae*	
dme-miR-4968-3p*	Supercontig_2.17	95	20	1	0	0.048	32.2	*C.neoformans/S. cerevisiae*	
dme-miR-4968-3p*	Supercontig_2.17	95	20	1	0	0.048	32.2	*C.neoformans/S. cerevisiae*	
dme-miR-4968-3p*	Supercontig_2.17	95	20	1	0	0.048	32.2	*C.neoformans/S. cerevisiae*	
dme-miR-4968-3p*	Supercontig_2.17	95.24	21	1	0	0.012	34.2	*C.neoformans/S. cerevisiae*	
dme-miR-4968-3p*	Supercontig_2.17	95.24	21	1	0	0.012	34.2	*C.neoformans/S. cerevisiae*	
dme-miR-4968-3p*	Supercontig_2.18	100	18	0	0	0.003	36.2	*C.neoformans/S. cerevisiae*	
dme-miR-4968-3p*	Supercontig_2.18	100	18	0	0	0.003	36.2	*C.neoformans/S. cerevisiae*	
dme-miR-4968-3p*	Supercontig_2.1	95	20	1	0	0.048	32.2	*C.neoformans/S. cerevisiae*	
dme-miR-4968-3p*	Supercontig_2.1	95	20	1	0	0.048	32.2	*C.neoformans/S. cerevisiae*	
dme-miR-4968-3p*	Supercontig_2.1	95	20	1	0	0.048	32.2	*C.neoformans/S. cerevisiae*	
dme-miR-4968-3p*	Supercontig_2.1	95	20	1	0	0.048	32.2	*C.neoformans/S. cerevisiae*	
dme-miR-4968-3p*	Supercontig_2.1	95	20	1	0	0.048	32.2	*C.neoformans/S. cerevisiae*	
dme-miR-4968-3p*	Supercontig_2.1	95	20	1	0	0.048	32.2	*C.neoformans/S. cerevisiae*	
dme-miR-4968-3p*	Supercontig_2.1	95	20	1	0	4.80E-02	32.2	*C.neoformans/S. cerevisiae*	
dme-miR-4968-3p*	Supercontig_2.1	95	20	1	0	4.80E-02	32.2	*C.neoformans/S. cerevisiae*	
dme-miR-4968-3p*	Supercontig_2.1	95	20	1	0	0.048	32.2	*C.neoformans/S. cerevisiae*	
dme-miR-4968-3p*	Supercontig_2.1	95.24	21	1	0	0.012	34.2	*C.neoformans/S. cerevisiae*	
dme-miR-4968-3p*	Supercontig_2.1	95.24	21	1	0	0.012	34.2	*C.neoformans/S. cerevisiae*	
dme-miR-4968-3p*	Supercontig_2.1	95.24	21	1	0	0.012	34.2	*C.neoformans/S. cerevisiae*	
dme-miR-4968-3p*	Supercontig_2.1	95.24	21	1	0	0.012	34.2	*C.neoformans/S. cerevisiae*	
dme-miR-4968-3p*	Supercontig_2.1	95.24	21	1	0	0.012	34.2	*C.neoformans/S. cerevisiae*	
dme-miR-4968-3p*	Supercontig_2.1	95.24	21	1	0	0.012	34.2	*C.neoformans/S. cerevisiae*	
dme-miR-4968-3p*	Supercontig_2.1	95.24	21	1	0	0.012	34.2	*C.neoformans/S. cerevisiae*	
dme-miR-4968-3p*	Supercontig_2.1	95.24	21	1	0	0.012	34.2	*C.neoformans/S. cerevisiae*	
dme-miR-4968-3p*	Supercontig_2.1	95.24	21	1	0	0.012	34.2	*C.neoformans/S. cerevisiae*	
dme-miR-4968-3p*	Supercontig_2.1	95.24	21	1	0	0.012	34.2	*C.neoformans/S. cerevisiae*	
dme-miR-4968-3p*	Supercontig_2.1	95.24	21	1	0	0.012	34.2	*C.neoformans/S. cerevisiae*	
dme-miR-4968-3p*	Supercontig_2.1	95.24	21	1	0	0.012	34.2	*C.neoformans/S. cerevisiae*	
dme-miR-4968-3p*	Supercontig_2.1	95.24	21	1	0	0.012	34.2	*C.neoformans/S. cerevisiae*	
dme-miR-4968-3p*	Supercontig_2.1	95.24	21	1	0	0.012	34.2	*C.neoformans/S. cerevisiae*	
dme-miR-4968-3p*	Supercontig_2.1	95.24	21	1	0	0.012	34.2	*C.neoformans/S. cerevisiae*	
dme-miR-4968-3p*	Supercontig_2.1	95.24	21	1	0	0.012	34.2	*C.neoformans/S. cerevisiae*	
dme-miR-4968-3p*	Supercontig_2.1	95.24	21	1	0	0.012	34.2	*C.neoformans/S. cerevisiae*	
dme-miR-4968-3p*	Supercontig_2.1	95.24	21	1	0	0.012	34.2	*C.neoformans/S. cerevisiae*	
dme-miR-4968-3p*	Supercontig_2.1	95.24	21	1	0	0.012	34.2	*C.neoformans/S. cerevisiae*	
dme-miR-4968-3p*	Supercontig_2.1	95.24	21	1	0	0.012	34.2	*C.neoformans/S. cerevisiae*	
dme-miR-4968-3p*	Supercontig_2.1	95.24	21	1	0	0.012	34.2	*C.neoformans/S. cerevisiae*	
dme-miR-4968-3p*	Supercontig_2.1	95.24	21	1	0	0.012	34.2	*C.neoformans/S. cerevisiae*	
dme-miR-4968-3p*	Supercontig_2.1	95.24	21	1	0	0.012	34.2	*C.neoformans/S. cerevisiae*	
dme-miR-4968-3p*	Supercontig_2.1	95.45	22	1	0	0.003	36.2	*C.neoformans/S. cerevisiae*	
dme-miR-4968-3p*	Supercontig_2.19	95	20	1	0	0.048	32.2	*C.neoformans/S. cerevisiae*	
dme-miR-4968-3p*	Supercontig_2.2	100	16	0	0	0.048	32.2	*C.neoformans/S. cerevisiae*	
dme-miR-4968-3p*	Supercontig_2.2	100	16	0	0	4.80E-02	32.2	*C.neoformans/S. cerevisiae*	

Query id[a]	Subject id[b]	% Identity[c]	Alignment length[d]	Mismatches[e]	Gap opens[f]	Evalue [g]	Bit score[h]	Fungi vesicle[i]	Reference[j]
dme-miR-4968-3p*	Supercontig_2.2	100	16	0	0	4.80E-02	32.2	*C.neoformans/S. cerevisiae*	
dme-miR-4968-3p*	Supercontig_2.2	100	16	0	0	4.80E-02	32.2	*C.neoformans/S. cerevisiae*	
dme-miR-4968-3p*	Supercontig_2.2	100	16	0	0	0.048	32.2	*C.neoformans/S. cerevisiae*	
dme-miR-4968-3p*	Supercontig_2.2	100	16	0	0	0.048	32.2	*C.neoformans/S. cerevisiae*	
dme-miR-4968-3p*	Supercontig_2.2	100	16	0	0	0.048	32.2	*C.neoformans/S. cerevisiae*	
dme-miR-4968-3p*	Supercontig_2.2	100	16	0	0	0.048	32.2	*C.neoformans/S. cerevisiae*	
dme-miR-4968-3p*	Supercontig_2.2	100	16	0	0	0.048	32.2	*C.neoformans/S. cerevisiae*	
dme-miR-4968-3p*	Supercontig_2.2	100	16	0	0	0.048	32.2	*C.neoformans/S. cerevisiae*	
dme-miR-4968-3p*	Supercontig_2.2	100	16	0	0	0.048	32.2	*C.neoformans/S. cerevisiae*	
dme-miR-4968-3p*	Supercontig_2.2	100	16	0	0	0.048	32.2	*C.neoformans/S. cerevisiae*	
dme-miR-4968-3p*	Supercontig_2.2	100	16	0	0	0.048	32.2	*C.neoformans/S. cerevisiae*	
dme-miR-4968-3p*	Supercontig_2.2	100	17	0	0	0.012	34.2	*C.neoformans/S. cerevisiae*	
dme-miR-4968-3p*	Supercontig_2.2	100	18	0	0	0.003	36.2	*C.neoformans/S. cerevisiae*	
dme-miR-4968-3p*	Supercontig_2.2	100	18	0	0	0.003	36.2	*C.neoformans/S. cerevisiae*	
dme-miR-4968-3p*	Supercontig_2.2	100	18	0	0	0.003	36.2	*C.neoformans/S. cerevisiae*	
dme-miR-4968-3p*	Supercontig_2.2	100	18	0	0	0.003	36.2	*C.neoformans/S. cerevisiae*	
dme-miR-4968-3p*	Supercontig_2.2	100	18	0	0	0.003	36.2	*C.neoformans/S. cerevisiae*	
dpe-miR-13a	Supercontig_2.11	100	16	0	0	0.048	32.2	*C.neoformans*	
dps-miR-2565-5p	Supercontig_2.3	100	16	0	0	0.048	32.2	*S. cerevisiae*	
dps-miR-968-3p*	Supercontig_2.3	100	16	0	0	0.06	32.2	*C. albicans*	
dre-miR-216a	Supercontig_2.18	100	17	0	0	0.012	34.2	*C. albicans*	
dwi-miR-13a	Supercontig_2.11	100	16	0	0	0.048	32.2	*C. albicans*	
gga-miR-1457	Supercontig_2.2	100	16	0	0	0.048	32.2	*C. albicans*	
gga-miR-1458	Supercontig_2.1	100	16	0	0	0.042	32.2	*S. cerevisiae*	
gga-miR-1692	Supercontig_2.1	100	17	0	0	0.009	34.2	*C. albicans/S. cerevisiae/C.neoformans*	
gga-miR-1692	Supercontig_2.2	100	17	0	0	0.009	34.2	*C. albicans/S. cerevisiae/C.neoformans*	
gga-miR-1814	Supercontig_2.12	100	16	0	0	0.036	32.2	*C.neoformans*	
gga-miR-29a-5p	Supercontig_2.4	100	17	0	0	0.012	34.2	*C. albicans*	
gga-miR-3540	Supercontig_2.5	100	16	0	0	0.048	32.2	*S. cerevisiae*	
gga-miR-466	Supercontig_2.1	100	17	0	0	0.014	34.2	*C.neoformans*	
gga-miR-466	Supercontig_2.11	100	16	0	0	0.054	32.2	*C.neoformans*	
gga-miR-466	Supercontig_2.17	100	16	0	0	0.054	32.2	*C.neoformans*	
gga-miR-466	Supercontig_2.3	100	16	0	0	0.054	32.2	*C.neoformans*	
gga-miR-466	Supercontig_2.8	100	16	0	0	0.054	32.2	*C.neoformans*	
gra-miR482	Supercontig_2.6	95	20	1	0	0.048	32.2	*C.neoformans*	
hsa-miR-320a	Supercontig_2.9	100	16	0	0	0.048	32.2	*C. albicans*	
hsa-miR-3620-5p	Supercontig_2.10	100	18	0	0	0.003	36.2	*C.neoformans/S. cerevisiae*	
hsa-miR-3620-5p	Supercontig_2.10	100	19	0	0	8.00E-04	38.2	*C.neoformans/S. cerevisiae*	
hsa-miR-3620-5p	Supercontig_2.10	100	20	0	0	2.00E-04	40.1	*C.neoformans/S. cerevisiae*	
hsa-miR-3620-5p	Supercontig_2.1	100	17	0	0	0.012	34.2	*C.neoformans/S. cerevisiae*	
hsa-miR-3620-5p	Supercontig_2.3	100	16	0	0	0.048	32.2	*C.neoformans/S. cerevisiae*	
hsa-miR-3620-5p	Supercontig_2.4	100	17	0	0	0.012	34.2	*C.neoformans/S. cerevisiae*	
hsa-miR-3620-5p	Supercontig_2.4	95.24	21	1	0	0.012	34.2	*C.neoformans/S. cerevisiae*	
hsa-miR-3620-5p	Supercontig_2.6	100	18	0	0	0.003	36.2	*C.neoformans/S. cerevisiae*	
hsa-miR-3620-5p	Supercontig_2.6	100	19	0	0	8.00E-04	38.2	*C.neoformans/S. cerevisiae*	
hsa-miR-3925-5p	Supercontig_2.2	100	16	0	0	0.048	32.2	*C. albicans*	

Query id[a]	Subject id[b]	% Identity[c]	Alignment length[d]	Mismatches[e]	Gap opens[f]	Evalue [g]	Bit score[h]	Fungi vesicle[i]	Reference[j]
hsa-miR-3976	Supercontig_2.31	100	16	0	0	0.06	32.2	*C.neoformans*	
hsa-miR-4507	Supercontig_2.10	95	20	1	0	0.036	32.2	*C.neoformans/S. cerevisiae*	
hsa-miR-4507	Supercontig_2.10	95	20	1	0	0.036	32.2	*C.neoformans/S. cerevisiae*	
hsa-miR-4507	Supercontig_2.3	100	16	0	0	0.036	32.2	*C.neoformans/S. cerevisiae*	
hsa-miR-4507	Supercontig_2.4	100	17	0	0	0.009	34.2	*C.neoformans/S. cerevisiae*	
hsa-miR-4507	Supercontig_2.6	95	20	1	0	0.036	32.2	*C.neoformans/S. cerevisiae*	
hsa-miR-4539	Supercontig_2.6	100	16	0	0	0.048	32.2	*C. albicans*	
hsa-miR-5194	Supercontig_2.11	100	16	0	0	0.048	32.2	*S. cerevisiae*	
hsa-miR-5195-3p*	Supercontig_2.4	100	17	0	0	0.011	34.2	*S. cerevisiae*	
hsv2-miR-H13	Supercontig_2.12	100	16	0	0	0.048	32.2	*S. cerevisiae*	
mml-miR-938	Supercontig_2.31	100	16	0	0	0.048	32.2	*C.neoformans*	
mml-miR-938	Supercontig_2.31	100	16	0	0	0.048	32.2	*C.neoformans*	
mmu-let-7e-3p*	Supercontig_2.2	100	16	0	0	0.048	32.2	*C. albicans/C.neoformans*	
mmu-miR-1895	Supercontig_2.10	95.24	21	1	0	0.012	34.2	*S. cerevisiae*	
mmu-miR-1903	Supercontig_2.12	100	16	0	0	0.048	32.2	*S. cerevisiae*	
mmu-miR-1903	Supercontig_2.2	100	16	0	0	0.048	32.2	*S. cerevisiae*	
mmu-miR-1903	Supercontig_2.3	100	16	0	0	0.048	32.2	*S. cerevisiae*	
mmu-miR-1903	Supercontig_2.3	100	16	0	0	0.048	32.2	*S. cerevisiae*	
mmu-miR-1903	Supercontig_2.4	100	17	0	0	0.012	34.2	*S. cerevisiae*	
mmu-miR-1903	Supercontig_2.7	100	16	0	0	0.048	32.2	*S. cerevisiae*	
mmu-miR-297a-5p	Supercontig_2.2	95.24	21	1	0	0.012	34.2	*C. albicans*	
mmu-miR-297a-5p	Supercontig_2.3	100	16	0	0	4.80E-02	32.2	*C. albicans*	
mmu-miR-297a-5p	Supercontig_2.3	100	17	0	0	1.20E-02	34.2	*C. albicans*	
mmu-miR-339-5p	Supercontig_2.3	100	16	0	0	5.40E-02	32.2	*C. albicans*	
mmu-miR-383-3p*	Supercontig_2.32	100	16	0	0	4.20E-02	32.2	*S. cerevisiae*	
mmu-miR-383-3p*	Supercontig_2.32	100	16	0	0	4.20E-02	32.2	*S. cerevisiae*	
mmu-miR-383-3p*	Supercontig_2.32	100	16	0	0	4.20E-02	32.2	*S. cerevisiae*	
mmu-miR-383-3p*	Supercontig_2.32	100	16	0	0	4.20E-02	32.2	*S. cerevisiae*	
mmu-miR-383-3p*	Supercontig_2.32	100	16	0	0	4.20E-02	32.2	*S. cerevisiae*	
mmu-miR-383-3p*	Supercontig_2.32	100	16	0	0	0.042	32.2	*S. cerevisiae*	
mmu-miR-383-3p*	Supercontig_2.8	100	16	0	0	0.042	32.2	*S. cerevisiae*	
mmu-miR-383-3p*	Supercontig_2.8	100	16	0	0	0.042	32.2	*S. cerevisiae*	
mmu-miR-383-3p*	Supercontig_2.8	100	16	0	0	0.042	32.2	*S. cerevisiae*	
mmu-miR-466f-5p	Supercontig_2.3	100	18	0	0	0.003	36.2	*C. albicans*	
mmu-miR-466h-3p*	Supercontig_2.1	100	17	0	0	0.008	34.2	*C.neoformans*	
mmu-miR-466h-3p*	Supercontig_2.11	100	18	0	0	0.002	36.2	*C.neoformans*	
mmu-miR-466h-3p*	Supercontig_2.2	100	16	0	0	0.03	32.2	*C.neoformans*	
mmu-miR-466h-3p*	Supercontig_2.3	100	16	0	0	0.03	32.2	*C.neoformans*	
mmu-miR-466h-3p*	Supercontig_2.3	100	18	0	0	0.002	36.2	*C.neoformans*	
mmu-miR-466h-3p*	Supercontig_2.6	100	17	0	0	0.008	34.2	*C.neoformans*	
mmu-miR-466h-5p	Supercontig_2.3	95	20	1	0	0.048	32.2	*C.neoformans*	
mmu-miR-466h-5p	Supercontig_2.6	100	16	0	0	0.048	32.2	*C.neoformans*	
mmu-miR-466j	Supercontig_2.1	100	19	0	0	9.00E-04	38.2	*S. cerevisiae*	
mmu-miR-466j	Supercontig_2.11	100	16	0	0	0.054	32.2	*S. cerevisiae*	
mmu-miR-466j	Supercontig_2.11	100	16	0	0	0.054	32.2	*S. cerevisiae*	
mmu-miR-466j	Supercontig_2.2	100	17	0	0	0.014	34.2	*S. cerevisiae*	

Query id[a]	Subject id[b]	% Identity[c]	Alignment length[d]	Mismatches[e]	Gap opens[f]	Evalue [g]	Bit score[h]	Fungi vesicle[i]	Reference[j]
mmu-miR-466j	Supercontig_2.3	100	16	0	0	0.054	32.2	*S. cerevisiae*	
mmu-miR-466j	Supercontig_2.3	100	16	0	0	0.054	32.2	*S. cerevisiae*	
mmu-miR-466j	Supercontig_2.3	100	16	0	0	0.054	32.2	*S. cerevisiae*	
mmu-miR-466j	Supercontig_2.3	100	16	0	0	0.054	32.2	*S. cerevisiae*	
mmu-miR-466j	Supercontig_2.3	100	17	0	0	0.014	34.2	*S. cerevisiae*	
mmu-miR-466j	Supercontig_2.3	100	18	0	0	0.003	36.2	*S. cerevisiae*	
mmu-miR-466j	Supercontig_2.3	100	20	0	0	2.00E-04	40.1	*S. cerevisiae*	
mmu-miR-466j	Supercontig_2.3	95.24	21	1	0	0.014	34.2	*S. cerevisiae*	
mmu-miR-466k	Supercontig_2.6	100	16	0	0	0.054	32.2	*C.neoformans/S. cerevisiae*	
mmu-miR-466k	Supercontig_2.8	100	16	0	0	0.054	32.2	*C.neoformans/S. cerevisiae*	
mmu-miR-466k	Supercontig_2.18	100	16	0	0	0.054	32.2	*C.neoformans/S. cerevisiae*	
mmu-miR-466k	Supercontig_2.3	100	16	0	0	0.054	32.2	*C.neoformans/S. cerevisiae*	
mmu-miR-466k	Supercontig_2.3	100	16	0	0	0.054	32.2	*C.neoformans/S. cerevisiae*	
mmu-miR-466k	Supercontig_2.3	100	16	0	0	0.054	32.2	*C.neoformans/S. cerevisiae*	
mmu-miR-466k	Supercontig_2.4	100	16	0	0	0.054	32.2	*C.neoformans/S. cerevisiae*	
mmu-miR-466k	Supercontig_2.4	100	16	0	0	0.054	32.2	*C.neoformans/S. cerevisiae*	
mmu-miR-466k	Supercontig_2.5	100	16	0	0	0.054	32.2	*C.neoformans/S. cerevisiae*	
mmu-miR-467a-3p*	Supercontig_2.22	100	16	0	0	0.048	32.2	*S. cerevisiae*	
mmu-miR-467a-3p*	Supercontig_2.4	100	16	0	0	0.048	32.2	*S. cerevisiae*	
mmu-miR-467a-3p*	Supercontig_2.9	100	16	0	0	0.048	32.2	*S. cerevisiae*	
mmu-miR-467g	Supercontig_2.10	100	18	0	0	0.003	36.2	*C. albicans/ P. brasiliensis*	
mmu-miR-467g	Supercontig_2.12	100	17	0	0	1.10E-02	34.2	*C. albicans/ P. brasiliensis*	
mmu-miR-467g	Supercontig_2.16	100	16	0	0	4.20E-02	32.2	*C. albicans/ P. brasiliensis*	
mmu-miR-467g	Supercontig_2.27	100	16	0	0	0.042	32.2	*C. albicans/ P. brasiliensis*	
mmu-miR-467g	Supercontig_2.3	95	20	1	0	0.042	32.2	*C. albicans/ P. brasiliensis*	
mmu-miR-467g	Supercontig_2.4	100	16	0	0	0.042	32.2	*C. albicans/ P. brasiliensis*	
mmu-miR-467g	Supercontig_2.5	100	18	0	0	0.003	36.2	*C. albicans/ P. brasiliensis*	
mmu-miR-467g	Supercontig_2.7	100	17	0	0	0.011	34.2	*C. albicans/ P. brasiliensis*	
mmu-miR-669f-3p*	Supercontig_2.10	100	16	0	0	0.054	32.2	*S. cerevisiae*	
mmu-miR-669f-3p*	Supercontig_2.13	100	16	0	0	0.054	32.2	*S. cerevisiae*	
mmu-miR-669f-3p*	Supercontig_2.16	100	17	0	0	0.014	34.2	*S. cerevisiae*	
mmu-miR-669f-3p*	Supercontig_2.2	100	16	0	0	0.054	32.2	*S. cerevisiae*	
mmu-miR-669f-3p*	Supercontig_2.22	100	16	0	0	0.054	32.2	*S. cerevisiae*	
mmu-miR-669f-3p*	Supercontig_2.3	95	20	1	0	0.054	32.2	*S. cerevisiae*	
mmu-miR-669f-3p*	Supercontig_2.4	100	16	0	0	0.054	32.2	*S. cerevisiae*	
mmu-miR-669f-3p*	Supercontig_2.4	100	18	0	0	0.003	36.2	*S. cerevisiae*	
mmu-miR-669f-3p*	Supercontig_2.7	100	16	0	0	0.054	32.2	*S. cerevisiae*	
mmu-miR-669f-3p*	Supercontig_2.9	100	16	0	0	0.054	32.2	*S. cerevisiae*	
mmu-miR-669f-3p*	Supercontig_2.9	100	16	0	0	0.054	32.2	*S. cerevisiae*	
mmu-miR-669f-5p	Supercontig_2.11	100	16	0	0	0.06	32.2	*S. cerevisiae*	
mmu-miR-669f-5p	Supercontig_2.2	100	17	0	0	0.015	34.2	*S. cerevisiae*	
mmu-miR-669f-5p	Supercontig_2.3	100	16	0	0	0.06	32.2	*S. cerevisiae*	
mmu-miR-669f-5p	Supercontig_2.3	100	16	0	0	0.06	32.2	*S. cerevisiae*	
mmu-miR-669f-5p	Supercontig_2.3	100	16	0	0	0.06	32.2	*S. cerevisiae*	
mmu-miR-669f-5p	Supercontig_2.3	100	16	0	0	0.06	32.2	*S. cerevisiae*	
mmu-miR-669f-5p	Supercontig_2.3	100	17	0	0	0.015	34.2	*S. cerevisiae*	

Query id[a]	Subject id[b]	% Identity[c]	Alignment length[d]	Mismatches[e]	Gap opens[f]	Evalue [g]	Bit score[h]	Fungi vesicle[i]	Reference[j]
mmu-miR-669f-5p	Supercontig_2.3	100	17	0	0	0.015	34.2	*S. cerevisiae*	
mmu-miR-669f-5p	Supercontig_2.3	100	20	0	0	2.00E-04	40.1	*S. cerevisiae*	
mmu-miR-669f-5p	Supercontig_2.6	100	16	0	0	6.00E-02	32.2	*S. cerevisiae*	
mtr-miR5227	Supercontig_2.1	100	16	0	0	0.048	32.2	*C. albicans*	
oan-miR-1346	Supercontig_2.10	100	16	0	0	0.03	32.2	*C.neoformans*	
oan-miR-1351-3p*	Supercontig_2.1	100	16	0	0	0.042	32.2	*S. cerevisiae/C.albicans/C.neoformans*	
oan-miR-1418-5p	Supercontig_2.3	100	16	0	0	0.042	32.2	*C.neoformans*	
oan-miR-1420e-5p	Supercontig_2.3	100	17	0	0	0.014	34.2	*P. brasiliensis*	
oan-miR-1421n-2-5p	Supercontig_2.1	100	17	0	0	0.012	34.2	*P. brasiliensis*	
oan-miR-1422q-5p	Supercontig_2.7	100	16	0	0	0.048	32.2	*S. cerevisiae*	
osa-miR1438	Supercontig_2.1	100	16	0	0	4.80E-02	32.2	*C. albicans*	
osa-miR1438	Supercontig_2.1	100	16	0	0	4.80E-02	32.2	*C. albicans*	
osa-miR1438	Supercontig_2.5	100	16	0	0	4.80E-02	32.2	*C. albicans*	
osa-miR1438	Supercontig_2.9	100	16	0	0	4.80E-02	32.2	*C. albicans*	
osa-miR1438	Supercontig_2.9	100	17	0	0	0.012	34.2	*C. albicans*	
osa-miR156k	Supercontig_2.1	100	16	0	0	0.042	32.2	*S. cerevisiae*	
osa-miR156k	Supercontig_2.8	100	16	0	0	0.042	32.2	*S. cerevisiae*	
osa-miR2921	Supercontig_2.14	100	16	0	0	0.06	32.2	*C.neoformans*	
osa-miR2921	Supercontig_2.46	100	16	0	0	0.06	32.2	*C.neoformans*	
osa-miR399c	Supercontig_2.3	100	16	0	0	0.042	32.2	*C.neoformans*	
osa-miR5144-5p	Supercontig_2.17	100	16	0	0	0.042	32.2	*C.neoformans/S. cerevisiae*	
osa-miR821c	Supercontig_2.12	95	20	1	0	0.06	32.2	*P. brasiliensis*	
osa-miR821c	Supercontig_2.4	100	16	0	0	0.06	32.2	*P. brasiliensis*	
pab-miR482d	Supercontig_2.1	100	16	0	0	0.042	32.2	*S. cerevisiae*	
pma-miR-130e	Supercontig_2.3	100	16	0	0	0.054	32.2	*P.brasiliensis*	
pma-miR-20a-5p	Supercontig_2.3	100	17	0	0	0.014	34.2	*C. albicans/C.neoformans*	
ppt-miR1047-5p	Supercontig_2.10	100	16	0	0	0.042	32.2	*P. brasiliensis*	
ppy-miR-1228	Supercontig_2.7	100	16	0	0	0.036	32.2	*S. cerevisiae*	
rno-let-7e-3p*	Supercontig_2.2	100	16	0	0	0.048	32.2	*C. albicans/C.neoformans*	
rno-miR-483-5p	Supercontig_2.13	100	16	0	0	0.048	32.2	*C.neoformans*	
rno-miR-483-5p	Supercontig_2.2	100	16	0	0	0.048	32.2	*C.neoformans*	
rno-miR-483-5p	Supercontig_2.6	95.24	21	1	0	0.012	34.2	*C.neoformans*	
rno-miR-487b-5p	Supercontig_2.3	100	16	0	0	0.054	32.2	*S. cerevisiae*	
sbi-miR821e	Supercontig_2.4	100	18	0	0	0.003	36.2	*C.neoformans*	
sha-miR-23b	Supercontig_2.11	100	16	0	0	0.03	32.2	*C. albicans*	
sme-miR-2149-5p	Supercontig_2.2	100	16	0	0	0.042	32.2	*C.neoformans*	
smo-miR1097	Supercontig_2.2	100	16	0	0	0.042	32.2	*C. albicans*	
ssc-miR-664-5p	Supercontig_2.2	100	16	0	0	0.054	32.2	*P. brasiliensis*	
tgu-miR-200a-5p	Supercontig_2.26	95	20	1	0	0.042	32.2	*C.albicans/S. cerevisiae*	
tgu-miR-2976	Supercontig_2.11	100	16	0	0	0.042	32.2	*C. albicans/C.neoformans/ P.brasiliensis*	
vvi-miR3631c	Supercontig_2.7	100	17	0	0	0.012	34.2	*C neoformans/S. cerevisiae*	
vvi-miR3631d	Supercontig_2.7	100	17	0	0	0.012	34.2	*S. cerevisiae/ P. brasiliensis*	

a)Identifier of the microRNAs;
b) Sequence;
c)Percent of the identity between the sequences;
d) Size of alignment between the sequences of microRNAs and the genome region of *P. brasiliensis* (*Pb*18);
e)Number of different bases between the aligned sequences;
f) Absence of complementary bases between the sequences;
g) Likelihood sequence analyzed to align randomly to other sequences in the database. false positive rate;

h) Normalized score obtained using the following formula: S'= (I S – ln K)/ln 2;
i) Microorganism which microRNAs have been described;
j) Article describing microRNAs in fungi vesicles; (*) microRNA star.
